# Quantitative proteomic analysis of the tizoxanide effect in vero cells

**DOI:** 10.1038/s41598-020-71634-2

**Published:** 2020-09-07

**Authors:** K. A. Yamamoto, K. Blackburn, E. Migowski, M. B. Goshe, D. T. Brown, D. F. Ferreira, M. R. Soares

**Affiliations:** 1 Department of Biochemistry, Institute of Chemistry, Federal University of Rio de Janeiro, Caixa Postal 68563, Rio de Janeiro, RJ 21941-909 Brazil; 2 Department of Molecular and Structural Biochemistry, North Carolina State University, Raleigh, NC USA; 3 Institute of Pediatrics and Puericulture Martagão Gesteira, Federal University of Rio de Janeiro, Rio de Janeiro, Brazil; 4 Department of Virology, Paulo de Góes Microbiology Institute, Federal University of Rio de Janeiro, Rio de Janeiro, Brazil

**Keywords:** Proteomics, Biotechnology

## Abstract

Nitazoxanide (NTZ) is effective against helminths and numerous microorganisms, including bacteria and viruses. In vivo, NTZ is metabolized into Tizoxanide (TIZ), which is the active circulating metabolite. With the emergence of SARS-Cov-2 as a Pandemic agent, NTZ became one of the molecules already approved for human use to engage clinical trials, due to results in vitro showing that NTZ was highly effective against the SARS-Cov-2, agent of COVID-19. There are currently several ongoing clinical trials mainly in the USA and Brazil involving NTZ due not only to the in vitro results, but also for its long-known safety. Here, we study the response of Vero cells to TIZ treatment and unveil possible mechanisms for its antimicrobial effect, using a label-free proteomic approach (LC/MS/MS) analysis to compare the proteomic profile between untreated- and TIZ-treated cells. Fifteen differentially expressed proteins were observed related to various biological processes, including translation, intracellular trafficking, RNA processing and modification, and signal transduction. The broad antimicrobial range of TIZ points towards its overall effect in lowering cell metabolism and RNA processing and modification. The decreased levels of FASN, HNRNPH and HNRNPK with the treatment appear to be important for antiviral activity.

## Introduction

**Figure 1:**
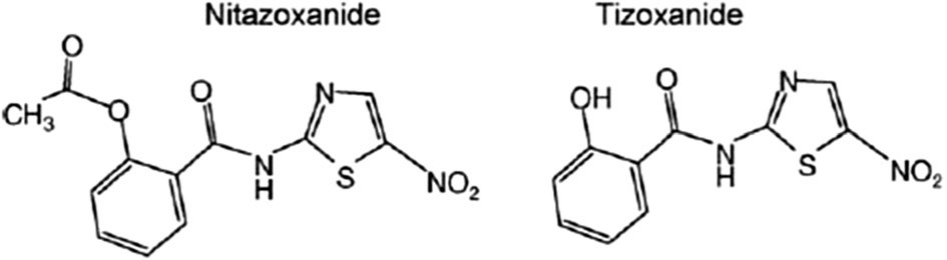
Chemical structure of nitazoxanide and tizoxanide (Korba et al.^18^).

Tizoxanide (TIZ) is the active form of nitazoxanide (NTZ), a benzamido-nitrothiazole compound, first developed as a veterinary anthelmintic with activity against intestinal nematodes and cestodes^1^. In 2002, the US Food and Drug Administration (FDA) approved oral suspension of NTZ for the treatment of intestinal protozoa infections such as *Crypstosporidium parvum* and *Giardia lamblia.* Currently, NTZ is indicated for the treatment of children and adults for a variety of protozoa, helminths and viral gastroenteritis (Alinia®, Romark Laboratories; Annita®, Farmoquímica S/A). NTZ is partially absorbed by the gastrointestinal tract and is rapidly hydrolysed by plasma esterases to its active form, TIZ^2^. NTZ and TIZ are equally effective in vitro^3^ (Fig. 1).

NTZ and TIZ have been thoroughly studied worldwide and have also been demonstrated to have activity in vitro and in vivo against a variety of anaerobic gram-positive and gram-negative bacteria as well as aerobic gram-positive bacteria^4,5^. These molecules have also been shown to inhibit *Mycobacterium tuberculosis* proliferation^6,7^. Furthermore, NTZ’s effectiveness has been reported against a wide range of parasites, such as *Ascaris lumbricoides, Balantidium coli, Blastocystit hominis, Clostridium difficile, Cyclospora cayetanensis, Echinococcus granulosus, Encephalitozoon intestinalis, Entamoeba histolytica, Enterocytozoon bieneusi, Fasciola hepatica, Hymenolepis nana, Isospora belli, Taenia saginata, Trichomonas vaginalis, Trichuris trichura,* and *Vittaforma corneae*^8–15^. In anaerobic organisms, NTZ activity is believed to be due to interference with the pyruvate:ferredoxin oxidoreductase (PFOR), an enzyme-dependent electron transfer reaction which is essential to anaerobic energy metabolism, which is not conserved in mammals^16^.

In recent years, NTZ and TIZ have been also reported to be effective against numerous RNA and DNA viruses such as rotavirus, norovirus, hepatitis B virus, hepatitis C virus, Japanese encephalitis virus, influenza virus and HIV^17–21^. It has been reported that these substances can inhibit different viruses in distinct manners. Rossignol et al.^22^ showed in 2009 that NTZ was able to block maturation of the influenza hemagglutinin at the post-translational stage. In the same year, Elazar et al.^23^ demonstrated that NTZ treatment of hepatitis C infected cells induced eukaryotic initiation factor-2α (eIF2α) phosphorylation via activation of its kinase, protein kinase activated by double-stranded RNA (PKR), which regulated the cell’s innate antiviral response. La Frazia et al.^17^ reported later that NTZ and TIZ inhibit rotavirus replication by interfering with viral morphogenesis.

The broad-spectrum nature of NTZ/TIZ also suggests that its ability to inhibit different viruses could be a consequence of its action on the host-regulated processes rather than its direct effect on the virus processes themselves. It seems plausible then, that the effect of these molecules could not only be a result of the direct action of the drug upon the virus or its proteins but might also stem from its influence on the way that the cell responds to the virus.

In December of 2019, China reported the outbreak of a new disease, caused by a new Coronavirus that was later named Severe Acute Respiratory Syndrome Coronavirus 2 (SARS-CoV-2). The disease was named COVID-19. On March 11, the World Health Organization declared COVID-19 a pandemic. Since then and to this date, SARS-CoV-2 has over 3.7 million confirmed cases and has killed over 260 thousand people. Although there are speculations about a vaccine on a fast track, most specialists are not very optimistic about a quick vaccine solution for COVID-19.

In vitro studies with nitazoxanide and tizoxanide were performed with canine coronavirus strain S-378, with a murine strain of coronavirus (MHV-A59) and a bovine coronavirus strain (BCoV-L9) with promising antiviral activity^24,25^. NTZ and TIZ had also its antiviral activity tested in vitro against the Middle East Respiratory Syndrome CoV (MERS-CoV) showing also effectivity^26^. Furthermore, Nitazoxanide was tested in China against SARS-CoV-2 (COVID-19 agent) using Vero E-6 cells and showed high antiviral activity^27^.

As the clinical trial results utilizing Nitazoxanide against SARS-Cov-2 infections start to build up, the more we know about the mode of action of this molecule, the better we can approach and rationalize about its use not only for COVId-19 but also for the other viruses being studied with this molecule.

In this work, we aimed to examine the response of Vero cells to TIZ treatment and unveil possible mechanisms for its antimicrobial effect. To this end, a label-free proteomic approach using liquid chromatography-tandem mass spectrometry (LC/MS/MS) was used as strategy. We performed a comparative proteomic analysis between untreated- or TIZ-treated Vero cells, highlighting differential abundance of proteins involved in cell processes. TIZ-treated Vero cells caused a decrease in several protein abundance levels related to different biological processes. It is our understanding that the way TIZ influences a broad range of cells and pathogens can bring new insights in the development of molecules with broad anti-pathogen action.

## Results

### Cell viability assay and TEM

**Figure 2:**
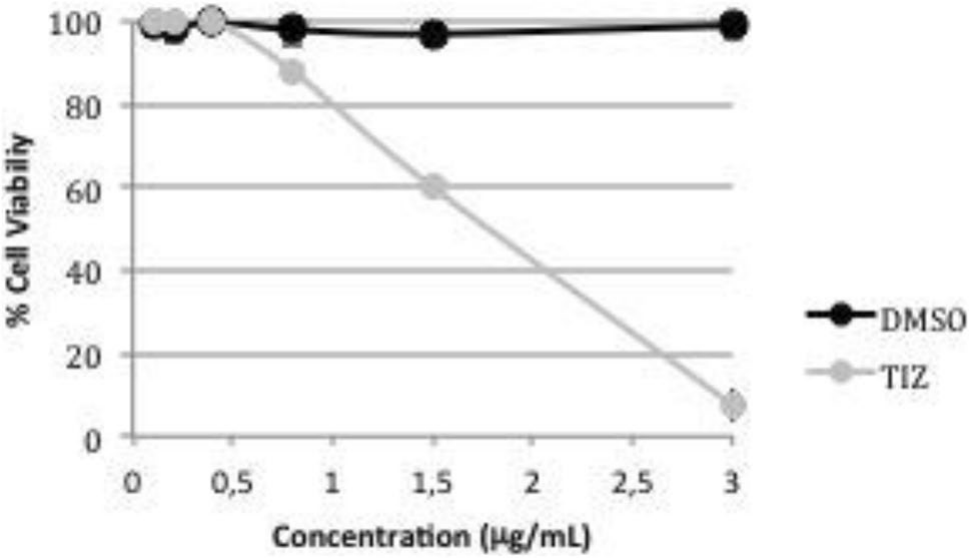
Viability of TIZ-treated Vero cells (72-h treatment) by neutral red dye uptake method. Data are presented as mean % cell viability of six replicates, compared to non-treated cell controls ± SD. The SD bars are obscured by the dot.

**Figure 3:**
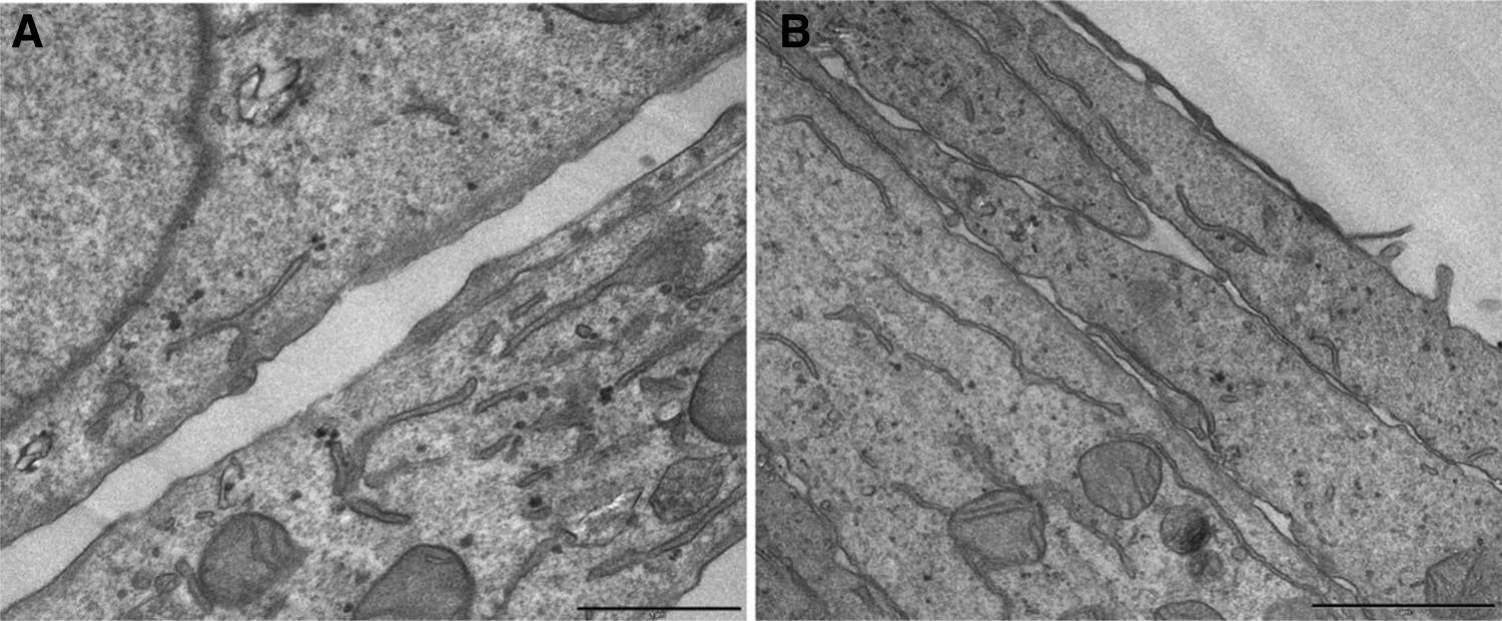
TEM of ultrathin sections of Vero cells (**A**) and Vero cells treated with 0.5 μg/mL TIZ for 24 h (**B**).

In order to evaluate the toxicity of TIZ in Vero cells, neutral red dye-uptake method was performed. The cytotoxicity assay showed no interference of the DMSO at the concentrations used in the samples in Vero cells and the TIZ influenced cell viability in a dose-dependent manner as shown in Fig. 2. The calculated CC_50%_ of TIZ was 1.77 μg/mL. A non-toxic concentration of TIZ, 0.5 μg/mL, was selected as a concentration for downstream experiments. TEM of ultrathin sections of Vero cells were also performed in an attempt to observe morphological changes or other phenomena such as inclusion bodies, alterations in cell membrane, or increases in the number of cytoplasmic vesicles the TIZ treatment could cause in comparison to untreated cells. However, no significant disturbances were observed (Fig. 3).

### 1D electrophoresis

**Figure 4:**
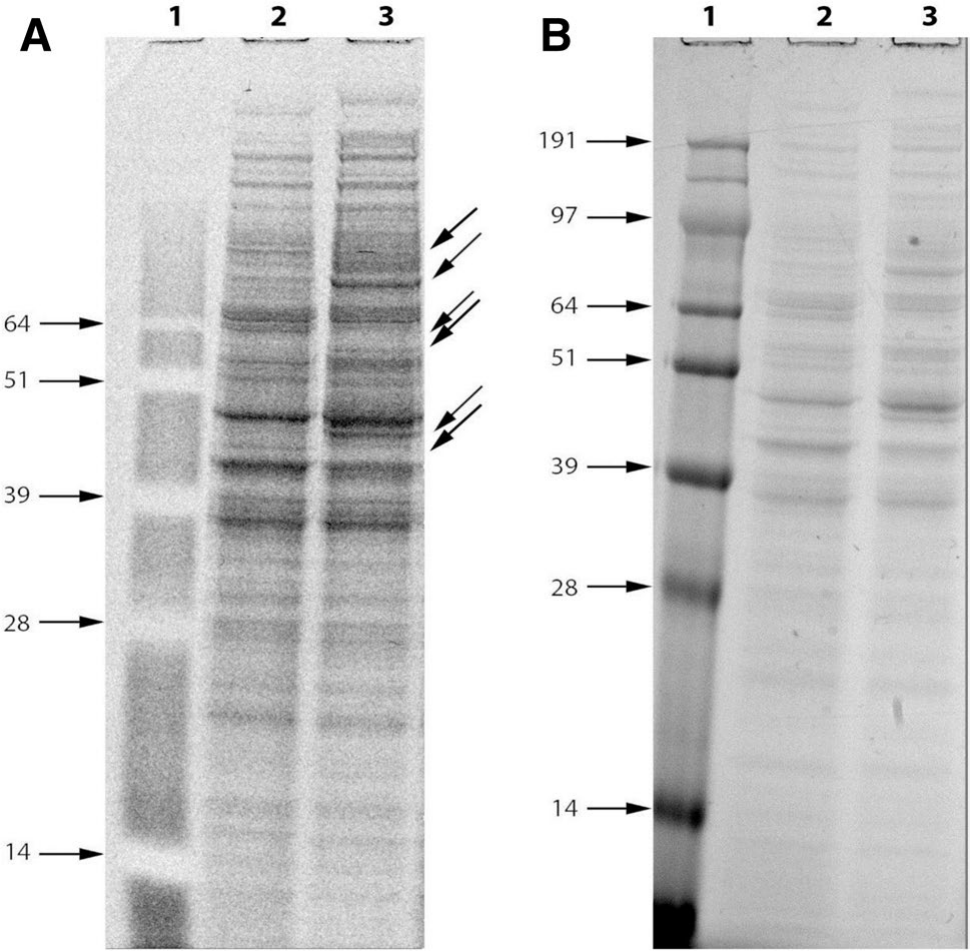
Gel image of total cell lysate proteins with fluorescent (**A**) and Coomassie blue (**B**) staining. A protein molecular weight standard ladder (kDa) is shown in lane 1 while mock- and TIZ-treated Vero cells for 24 h are shown in lane 2 and 3, respectively. Arrows show examples of bands that are differentially visible in the gel.

To visualize the changes in protein profile of Vero cell in response to TIZ treatment, total protein of confluent Vero cells untreated and TIZ-treated were extracted, dosed and loaded for SDS-PAGE. Fluorescent and Coomassie blue staining showed that the proteins were successfully extracted (Fig. 4). As indicated in Fig. 4, it is possible to observe several bands in the SDS-PAGE gel becoming highly intense or almost disappearing with TIZ treatment compared to the untreated control. These differences in band intensity slightly changed the protein profile of Vero cells mainly above 28 kDa and were similar in all replicates.

### Protein identification and functional protein identification and functional categorization

**Figure 5:**
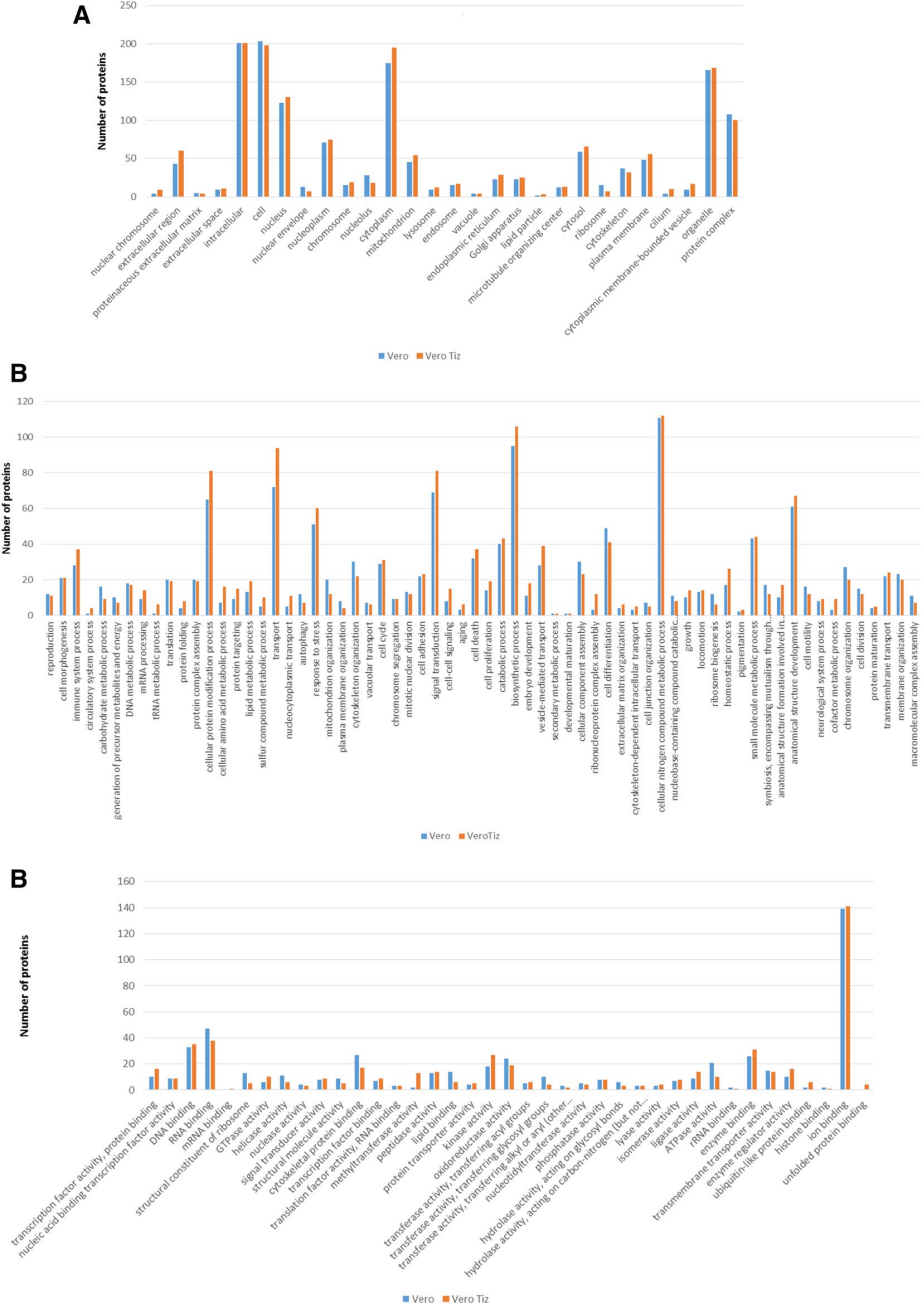
Gene Ontology analysis of proteins by AgBase (www.agbase.msstate.edu) in three categories: cellular component (**A**), biological process (**B**), and molecular function (**C**).

In order to concentrate the sample and quantify the differences in overall protein abundance levels in untreated- and TIZ-treated Vero cells, FASP digestion using a 30 kDa cut-off filter was performed. A total of 1,303 proteins were identified (Supplementary Table 1). Gene Ontology analysis identified 133 functional subcategories of proteins (www.agbase.msstate.edu), and three primary categories: cellular component (28), molecular function (39), and biological process (66) (Fig. 5).

The greatest differences were observed in the biological process subcategory: ribonucleoprotein complex assembly, nucleocytoplasmic transport, tRNa metabolic, circulatory system, cofactor metabolic and cellular amino acid metabolic process. They presented more than a 100% increase in the number of proteins in treated cells compared to the untreated cells.

For cellular component, TIZ-treated cells had an increase in the number of proteins associated with cilium, nuclear chromosome and cytoplasmic membrane-bounded vesicle while ribosome proteins were decreased. In the molecular function category, more proteins were associated with methyltransferase activity, ubiquitin-like protein binding and GTPase activity and fewer proteins associated with structural constituent ribosome, transferase activity, transferring glycosyl groups, lipid binding and ATPase activity in TIZ-treated cells.

### Quantitative analysis

MaxQuant/Andromeda-Perseus identified 1,303 proteins and validated 850 proteins in both samples, using the parameters described in the Methods section. According to the statistical analysis, FASP method was able to identify 15 differentially expressed proteins at the *p* < 0.05 level, 12 of them with higher abundance in mock-treated samples and 3 in TIZ-treated samples, involved in different biological process such as translation, ribosomal structure and biogenesis, intracellular trafficking, secretion and vesicular transport, signal transduction, chromatin structure and dynamics, RNA processing and modification and cytoskeleton (Table 1).

**Table 1:** Differential protein abundance levels in untreated and TIZ-treated Vero cells (24-h treatment) using FASP LC/MS/MS analysis. Samples in which the proteins presented statistically significant higher expression are marked (x).

Protein ID	Protein description	Peptides	Fold change	*t* test *p* value	Biologic process	Higher expression	
						Control	TIZ
gi|635092401	Peptidyl-prolyl cis–trans isomerase FKBP10	8	0.94	0.020	Posttranslational modification, protein turnover, and chaperones	x	
gi|635013418	FACT complex subunit SSRP1	5	0.93	0.034	Chromatin structure and dynamics; Transcription, replication, recombination and repair	x	
gi|635096172	Spliceosome RNA helicase DDX39B	12	0.93	0.049	RNA processing and modification	x	
gi|635125783	Heterogeneous nuclear ribonucleoprotein H	8	0.92	0.010	RNA processing and modification	x	
gi|51863477	Glutamate dehydrogenase	9	0.88	0.009	Metabolism; energy production and conversion	x	
gi|635109727	Tyrosine–tRNA ligase, cytoplasmic	7	0.86	0.029	Metabolism; translation, ribosomal structure and biogenesis	x	
gi|635093656	Fatty acid synthase	59	0.85	0.012	Metabolism	x	
gi|635057694	Aspartate–tRNA ligase, cytoplasmic	8	0.82	0.039	Metabolism; translation, ribosomal structure and biogenesis	x	
gi|635091792	Glial fibrillary acidic protein	9	0.73	0.039	Other function	x	
gi|635042304	Nucleobindin-1	3	0.54	0.014	Other function	x	
gi|635031591	Eukaryotic translation initiation factor 3 subunit C	4	0.24	0.012	Translation, ribosomal structure and biogenesis	x	
gi|635105802	Ras-related protein Rap-1A	3	0.10	0.002	Intracellular trafficking, secretion, and vesicular transport; signal transduction	x	
gi|635066390	Dynactin subunit 2	3	1.31	0.021	Intracellular trafficking, secretion, and vesicular transport; cell cycle control and division		x
gi|635067826	Cytoskeleton-associated protein 4	14	1.19	0.049	Cytoskeleton		x
gi|635022540	Eukaryotic translation initiation factor 6	7	1.08	0.029	Translation, ribosomal structure and biogenesis		x

## Discussion

NTZ is a safe clinical approved antiparasitic agent indicated for the treatment of a variety of gastrointestinal infections and, along with its active form, TIZ, has been shown to inhibit a broad range of viruses and microorganisms. With the ongoing COVID-19 pandemic, clinical studies with Nitazoxanide and its efficacy against SARS-CoV-2 infections (COVID-19) are currently underway. In this context, more information on how the host cell responds to TIZ would be interesting to unravel other intracellular mechanisms responsible for its antiviral and antimicrobial activity. In 2015, MS-based proteomic approaches were used as a new and promising tool to help understanding cellular changes in metabolism due to a treatment exposure^28^. In this study, a label free MS-based proteomic approach was used to identify proteins whose abundance is altered by TIZ treatment and to show possible cell processes disturbed or enhanced by it. A filter-aid sample preparation (FASP) method was adopted for protein digestion strategy. Cell viability assay was performed to assess the Vero cells response to TIZ treatment and to help us set our treatment protocol while TEM was performed for visualization of cell morphology and 1D electrophoresis for visualizations of proteome profile.

It has been reported that the group of proteins with altered abundance in proteomic studies of virus-infected cells or cells exposed to some level of stress, such as drug action, is small. Besides, the changes can be subtle and, sometimes, with fold change less than 2^29^. Even using different approaches as isotopic labelling or Differential Gel Electrophoresis (DIGE), the number of detected proteins with altered abundance does not increase dramatically^30,31^. When we considered a *p* value < 0.05, as demonstrated in Table 1, the FASP label-free LC/MS/MS methodology was able to identify 15 cellular proteins with different abundance levels when treated with TIZ (3 had increased levels and 12 had decreased levels). This indicates a subtle effect of TIZ on the cell.

Among the decreased proteins in metabolism, we highlight fatty acid synthase (FASN), a protein responsible to synthesize palmitate from acetyl-CoA and malonyl-CoA in a reaction that requires NADPH. Palmitate, the core of fatty acid production, can be further metabolized into a variety of long chain fatty acids that will be used in lipid production for membrane biosynthesis and lipid droplet formation^32^. Importantly, many viruses induce and require fatty acid synthesis at some stage of their replication cycle and the interference in this pathway could affect the virus production. The use of critical inhibitors of enzymes for fatty acid synthesis such as acetyl-CoA carboxylase and fatty acid synthase led to a significant decrease in the production of infectious cytomegalovirus, influenza A, hepatitis C and dengue viruses^33^. In the case of dengue virus, non-structural protein NS3 seems to be responsible for FASN recruitment, and lipidomics of mosquito infected cells showed that some sphingolipids and phospholipids were upregulated by dengue infection^34^. Therefore, FASN inhibition caused by TIZ treatment could explain the wide antiviral action of this substance. However, the specific mechanism by which inhibition of fatty acid biosynthesis interferes with the replication of these viruses has not yet been determined. Studies suggest that possible changes in membrane composition required for viral replication, assembly or budding, decreases in envelope phospholipid synthesis or delays in the modification of fatty acids from proteins would be among the possible targets caused by inhibition of FASN in infected cells^33,35^.

Ras-related protein Rap-1a, a signal transduction-related protein, was detected in lower levels with the TIZ treatment. Signal transduction processes are, in many aspects, protein-driven events. Changes in protein level, activity, localization or interactions allow cells to react to a specific event and also to vary the sensitivity, duration and dynamics of the response^36^. Ras-related protein Rap-1a have been described to interact with several different pathways, namely MAPK, ERK, GRK2, (JAK)/STAT, and NF-kB^37–39^. In addition, they have been implicated in other biological events, including Intracellular trafficking, secretion and vesicular transport, Transcription, replication, recombination and repair, and defense mechanisms. Thus, more information is needed about these complex interactions at both the cellular and whole organism levels for a better understanding of the TIZ antimicrobial activity.

The abundance levels of two proteins related to RNA processing and modification were also altered with the TIZ treatment. Newly synthesized RNA molecules undergo different modifications before their translation in the cytoplasm. These processing steps include specific modifications of RNA nucleotides at the ends of the primary transcript or at internal positions, and removal of internal extra RNA sequences^40^. Levels of spliceosome RNA helicase DDX39B and heterogeneous nuclear ribonucleoprotein H (HNRNPH) were decreased with the treatment. These numbers are in accordance to the overall number of ribosome proteins, decreased with the treatment.

HNRNPs are a large Family of RNA-binding proteins involved in alternative splicing, mRNA stabilization, and transcriptional and translational regulation [reviewed in Ref.^41^], and they have been reported to interact with several virus proteins and RNA. Studies demonstrate that HNRNPH is upregulated with dengue infection in cell culture in addition to interacting with dengue non-structural protein 1, helping the virus to propagate in the cell^42^. HNRNPH also seems to be necessary to bind to specific retroviruses proteins to control the RNA splicing needed to HIV-1 and Rous sarcoma virus life cycle^43^. Therefore, decrease in the amount of HNRNPH account for part of the broad-spectrum antiviral activity of TIZ.

In addition, three proteins with increased levels with the treatment stood out: dynactin subunit 2, eukaryotic translation initiation factor 6 (EIF6) and cytoskeleton associated protein 4 (CKAP4).

Dynactin subunit 2, one of the increased proteins in intracellular trafficking, secretion and vesicular transport, is part of the dynactin complex, acting as cofactor for the dynein, a minus-ended-directed microtubule associated motor responsible for retrograde transport in eukaryotic cells. The dynactin-dynein motor complex has been implicated in several important subcellular functions involving intracellular organelle transport^44^, including the transport of many viruses from cytosol to their site of replication^45^. Due to the great diversity of viruses and their replication mechanisms and requirements, each antiviral activity must be evaluated individually. On the other hand, Harrinson et al.^46^ demonstrated that full maturation of phagosomes of the murine monocyte/macrophage line depends on dynein-dynactin association to acquire its antimicrobial properties required for pathogen elimination.

EIF6 is a protein from the translation initiation group that does not function as an initiation factor and is known to be important in ribosome biogenesis by regulating cellular levels of free 60S subunit^47^. In 2001, Oh, Filler and Cho^48^ demonstrated that overexpression of eIF6 enhanced the production of histamine and IL-2 by murine mast cells. Histamine affects cells of the innate and adaptive immune responses by recruiting NK cells and eosinophils, protecting intestinal epithelial cells from pathogenic bacterial infection, up-regulating dendritic cells antigen-presenting capacity or exerting diverse effects on T-cell polarization and on B-cell immunoglobulin secretion [reviewed in Ref.^49^]. IL-2 plays important roles in immune response, promoting T-cell proliferation, survival, cytolytic activity, NK cell activity, development of Treg cells and activation-induced cell death, among others^50^. Altogether, histamine and IL-2 production indirectly stimulated by TIZ treatment could explain its success against several different pathogens in humans.

CKAP4 also known as cytoskeleton-linking membrane protein 63 (CLIMP-63) or p63, is a stable and abundant type II transmembrane protein^51^ predominantly located in the RER and present in higher eukaryotes^52^. It has multiple functions including maintaining ER structure, ribosome anchoring in the RER, RER anchoring to the cytoskeleton via microtubule interaction, besides acting as a receptor for different ligands. In addition, Li et al.^53^ demonstrated that expression of CKAP4 discouraged cell cycle progression and reduced the proliferation ability of hepatocellular carcinoma cells.

The current results in this work confirm the safe clinical use of TIZ. It presented low toxicity and subtle changes in protein profile were observed. These changes do not seem to be responsible for triggering a specific protein for antimicrobial effect in general, but to act on the cell as a whole. Nevertheless, the decreased levels of FASN, HNRNPH and HNRNPK with the treatment appear to be in part responsible for the antiviral success against several viruses. The biosynthetic pathway of fatty acids has already been suggested as targets for development of therapeutics that inhibit the replication of DENV and other enveloped viruses^58^. However, most of the studies are focused on virus–cell interaction and there is still a lot to understand about how cellular metabolism changes influence on host response to viral infection at the organism level, from the production of hormones to immune responses.

In this work, we report for the first time a differential proteomic analysis between Vero cell cultures mock- and TIZ-treated by label-free LC/MS/MS analysis. This proteomics study provides valuable data for a better understanding of the roles played by host cell proteins during TIZ treatment. The broad antimicrobial range of TIZ points towards its overall effect in cell metabolism and RNA processing and modification. Nevertheless, further functional analysis is necessary. The list of proteins is likely to be further extended by improving the proteomic analysis, i.e. by increasing the number of replicates in the analysis, providing some level of protein fractionation, or performing a longer peptide separation coupled to a more sensitive and faster scanning mass spectrometer. Our data provides evidence that the knowledge of the functional expression of proteins may be of value for therapeutic purposes.

## Methods

### Cell culture and tizoxanide

Vero cells (African Green monkey kidney, ATCC CCL-81) were maintained at 37 °C with 5% CO_2_, in MEM supplemented with 10% FBS, 5% tryptose phosphate broth, 2 mM L-glutamine, 10 mM HEPES (pH 7.4) and nonessential amino acids (1:100 dilution of nonessential amino acids, no. 11140, Gibco/Life Technologies, Carlsbad, CA, USA). To perform the experiments, 3 × 10^5^ cells/mL were cultured in plates or 6 cell culture bottles for 24 h. TIZ was supplied by Farmoquimica S/A (Rio de Janeiro, BRA), diluted in DMSO and kept at − 20 °C.

### Cell viability

The cytotoxicity of TIZ was determined by modified neutral red dye-uptake method from Borenfreund and Puerner^54^. Briefly, 3 × 10^5^ cells/mL were cultured in 96-well microplates until confluence. The cells were treated with different concentrations of TIZ for 72 h and the culture medium was replaced by 50 μg/mL of neutral red solution. After 3 h, at 37 °C, 5% CO_2_, cells were fixed with 20% formaldehyde and the neutral red was extracted with 50% methanol and 1% acetic acid for absorbance measurement at 490 nm. Cell viability percentage was calculated by the formula [100 − (*At*/*Ac* × 100], which *At* and *Ac* refer to the absorbance of test substance and control, respectively. The concentration of the test substance capable of reducing cell viability by 50% in comparison to non-treated cell control (CC_50%_) was calculated by regression analysis. Cells treated with equal amounts of DMSO and non-treated cells were used as controls.

### Transmission electron microscopy (TEM)

Approximately 3 × 10^5^ cells/mL were grown for 24 h in 6-well plates. Confluent cells were washed with PBS and treated or mock-treated for 24 h at 37 °C and 5% CO_2_ with 0.5 μg/mL TIZ. After 24 h, monolayers were fixed in a solution containing 2.5% glutaraldehyde in 0.1 M cacodylate buffer, pH 7.2, post-fixed for 1 h with 1% OsO_4_ in 0.1 M cacodylate buffer, pH 7.2, plus 0.8% potassium ferrocyanide, dehydrated in ethanol, and flat embedded in Polybed (Polysciences®, Warrington, PA, USA). Ultrathin sections were placed in 300 mesh grids and stained with uranyl acetate and lead citrate^55^. Then, samples were observed in a Zeiss 900 transmission electron microscope at 80 kV.

### Protein sample preparation for proteomic analysis

The cells were grown and treated or mock-treated for 24 h as described above. After incubation, cells were washed with PBS and lysed by M-PER™ Mammalian Protein Extraction Reagent (Thermo Scientific, Rockford, IL, USA), according to manufacturer’s instructions. Briefly, culture medium was carefully removed and the cells were washed once in PBS. Then, 300 μL of M-PER® Reagent was added to each well and the plate was agitated gently for 5 min. The lysate was centrifuged at 14,000×*g* for 10 min and the supernatant was collected and kept at − 8 °C for further analysis. The protein concentration was determined by Coomassie plus protein assay reagent (Pierce Biotechnology, Rockford, IL, USA).

### 1D electrophoresis

Fifteen micrograms of protein per sample was loaded on two 1-mm 10-well 12% NuPAGE® Bis–Tris gel (Invitrogen/Life Technologies, Carlsbad, CA, USA) and the proteins were separated for 1 h at 180 V. The protein bands of one SDS-PAGE gel were visualized using fluorescent staining (SYPRO® Ruby Gel Stain, Molecular Probes, Eugene, OR, USA) and the proteins of the other SDS-PAGE gel were visualized using NuPAGE® Colloidal Blue (Invitrogen/Life Technologies) staining overnight at room temperature and destained with Milli-Q water until the background was transparent. The experiments were performed in technical two replicates and all sample gels were electrophoresed under identical conditions.

### Protein digestion

Filter-aided sample preparation (FASP) method was used for the purification and on-filter digestion of proteins, based on Wiśniewski et al.^56^. All buffer exchanges were carried out by centrifugation at 10,000×*g* for 15 min. 200 μg of protein extract was reduced with a final concentration of 5 mM DTT at 56 °C for 30 min and transferred into a 500 μL Sartorius Vivacon 30,000 MWCO centrifugal unit (Fisher Scientific, Rockford, IL, USA). Then, 200 μL of UA buffer (8 M urea, 0.1 M Tris, pH 8.5) was added and the sample was centrifuged (step repeated once). For alkylation of reduced cysteine bonds, 100 μL of UA buffer containing 50 mM iodoacetamide was added and incubated in the dark for 20 min at room temperature followed centrifugation. Three 100 μL UA buffer exchanges were used to remove residual alkylating agent, followed by three buffer exchanges with 100 μL of ABC buffer (50 mM ammonium bicarbonate buffer, pH 8). A volume of 50 μL of ABC buffer containing 1:100 ratio of sequencing-grade modified trypsin (Promega) to protein was added, and the tubes were incubated at 37 °C in a water bath for 18 h. Two rounds of 40 μL of ABC buffer were used to elute the peptide-rich solution, and then the samples were dried down using vacuum centrifugation. Three biological samples for each condition were prepared in this manner and used for sequent LC/MS/MS analysis.

### Liquid chromatography-tandem mass spectrometry (LC/MS/MS) analysis

Tryptic digests of each sample obtained by the FASP method were analyzed by LC/MS/MS using an Easy NanoLC 1,000 (Thermo Scientific) coupled to an Orbitrap Elite mass spectrometer (Thermo Scientific). Digests were desalted and preconcentrated onto a 2 cm × 100 µm i.d. Pepmap C18 (5 µm particle size) (Thermo Scientific), and then eluted onto and separated using a self-packed PicoFrit (New Objective, Woburn, MA, USA) 75 µm id × 25 cm Magic C18 column (3 µm particle size) with a 60 min linear gradient from 2% mobile phase B to 40% mobile phase B (A = 2% acetonitrile in water, 0.1% formic acid; B = acetonitrile, 0.1% formic acid). An electrospray voltage of 2.8 kV was applied to the PicoFrit column to ionise peptides in the nanoelectrospray ion source of the Obitrap Elite with a heated capillary temperature of 275 °C. The data acquisition of MS (scan range of *m/z* 400–2000) and MS/MS (scan range of *m/z* 140–2000) were collected utilizing the Orbitrap analyser. A top 5 method with higher-energy collisional activation (HCD) for product ion generation in the HCD cell was used with normalized collision energy setting of 27 V to induce precursor ion fragmentation (+ 1 charge states were excluded).

### Protein identification and analysis

Raw LC/MS/MS datafiles were processed using MaxQuant software^57^ and database searched using the integrated Andromeda^58^ search engine against the non-redundant database of the National Center of Biotechnology Information (NCBI) containing entries from *Chlorocebus sabaeus* as well as the two protein standards bovine serum albumin and rabbit glycogen phosphorylase (62,148 entries). Trypsin was defined as the digesting enzyme, along with a maximum of two missed tryptic sites, whereas fixed carbamidomethyl Cys modification and variable oxidized Met modification were permitted. A false discovery rate (FDR) of 0.05 was utilized. Otherwise, default MaxQuant and Andromeda parameters were used for processing and searching.

For statistical analysis, the MaxQuant ProteinGroups report was imported into Perseus (www.perseus-framework.org), and protein label free quantification (LFQ) intensities based on extracted ion chromatograms were used to compare expression differences between sample groups. Data preprocessing prior to analysis of variance (ANOVA) included the following steps: Log(2) transformation of protein intensities and replacing missing data with a value approximating the lower limit of detection. Only proteins that were observed in three out of three replicates for at least one treatment group were retained and subjected to ANOVA. Statistically significant proteins were retained at the *p* < 0.1 significance level.

The identified and validated proteins from protein digestion were classified according to gene ontology (GO) terms in cell component, biological process and molecular function by AgBase software (www.agbase.msstate.edu). The uncharacterized proteins were classified according to GO terms and protein function from *Homo sapiens* proteins searched at Universal Protein Resource (Uniprot) catalog (www.uniprot.org) and Kyoto Encyclopedia of Genes and Genomes (KEGG) database (www.genome.jp). To reduce the large number of GO terms, the biological process was divided into 15 terms: Cell cycle control and cell division, chromatin structure and dynamics, cytoskeleton, defense mechanisms, energy production and conversion, Inorganic ion transport and metabolism, intracellular trafficking, secretion, and vesicular transport, membrane organization, metabolism (including carbohydrate, amino acids and lipids transport and metabolism), post-translational modification, protein turnover, and chaperones, RNA processing and modification, secondary metabolites biosynthesis, transport and catabolism, signal transduction, transcription, replication, recombination and repair, translation, ribosomal structure and biogenesis, and other function.

## Supplementary information

Supplementary Table 1.

## Data Availability

All data generated or analyzed during this study are included in this published article and the Supplementary Table and deposited to the online database MassIVE (https://massive.ucsd.edu) as Dataset MSV000085664.
