# Supplementary material for: Quantitative proteomic analysis of the tizoxanide effect in vero cells
Source: Sci Rep. 2020 Sep 7;10:14733. doi: 10.1038/s41598-020-71634-2 (PMC7477200; doi:10.1038/s41598-020-71634-2)
Supplement: Supplementary file 1 — Supplementary Table 1. [file 41598_2020_71634_MOESM1_ESM.pdf]

Quantitative proteomic analysis of the tizoxanide effect in vero cells

Yamamoto KA[a]; Blackburn K[b];Migowski E[c]; Goshe M[b]; Brown DT[b]; Ferreira DF[b,d]; Soares MR[a]

Log(2) iBAQ Protein Abundance

Vero	Vero	Vero	VeroTIZ	VeroTIZ	VeroTIZ	Fold Change VeroTIZ/Vero	p-value	Protein Accession	Protein Description
19.5	19.8	19.5	16.9	17.3	17.2	0.0032	0.0001	gi|635042304	nucleobindin-1
20.6	20.9	20.5	18.7	18.2	18.3	0.0054	0.0002	gi|635145430	nucleobindin-1-like
18.9	19.2	18.2	15.0	15.1	15.4	0.0002	0.0003	gi|635092431	eukaryotic translation initiation factor 1
19.5	19.5	19.5	19.7	19.8	19.7	1.6323	0.0032	gi|635060244	isocitrate dehydrogenase [NADP] cytoplasmic
18.7	18.9	18.5	18.0	17.7	17.9	0.1451	0.0037	gi|635018899	dolichyl-diphosphooligosaccharide--protein glycosyltransferase subunit STT3A
14.4	15.0	14.0	16.0	16.3	16.4	56.1811	0.0048	gi|635040288	trans-1,2-dihydrobenzene-1,2-diol dehydrogenase
16.5	16.5	16.6	16.0	16.3	15.9	0.3502	0.0161	gi|635138256	condensin complex subunit 3
20.9	21.2	21.1	21.3	21.5	21.5	2.1521	0.0218	gi|635072330	endoplasmic reticulum resident protein 44
12.0	13.9	12.2	15.2	14.4	15.8	252.6989	0.0286	gi|635050086	WASH complex subunit strumpellin
20.0	19.9	20.1	20.1	20.2	20.2	1.6383	0.0311	gi|635113027	protein transport protein Sec61 subunit gamma
16.2	15.8	16.1	16.4	17.0	16.9	5.4736	0.0321	gi|635129660	inverted formin-2 isoform X4
24.6	25.0	24.8	24.3	24.4	24.5	0.3747	0.0348	gi|635045164	40S ribosomal protein S3a
21.0	21.2	20.3	19.7	20.1	19.1	0.0605	0.0370	gi|635116140	calumenin isoform X2
18.1	18.1	17.9	17.4	17.6	17.8	0.3961	0.0457	gi|635089815	retinoid-inducible serine carboxypeptidase isoform X2
19.9	19.9	19.6	20.1	20.2	20.5	2.8380	0.0470	gi|635122798	coatomer subunit gamma-1
25.6	25.8	25.5	25.4	25.4	25.4	0.5875	0.0498	gi|635125337	nucleophosmin isoform X1
19.3	19.5	18.9	19.7	19.6	19.8	2.8046	0.0576	gi|635030501	heat shock protein 75 kDa, mitochondrial
18.6	19.7	19.5	20.1	20.2	20.1	7.5649	0.0637	gi|635110205	dnaJ homolog subfamily C member 8 isoform X2
17.9	18.3	18.3	18.5	18.6	19.0	3.2185	0.0648	gi|635060927	aspartyl aminopeptidase isoform X3
20.6	20.6	20.4	20.2	20.3	20.3	0.6023	0.0692	gi|635090268	septin-9 isoform X12
16.5	16.3	15.5	17.1	17.0	16.7	6.4772	0.0702	gi|635022070	ribosome-binding protein 1 isoform X4
25.5	25.6	25.7	26.0	26.0	25.7	2.0886	0.0704	gi|635082673	14-3-3 protein theta
19.1	19.4	18.6	18.3	18.6	18.3	0.2478	0.0705	gi|635146182	sterol-4-alpha-carboxylate 3-dehydrogenase, decarboxylating
22.8	23.1	22.6	22.6	22.4	22.2	0.3295	0.0709	gi|635059421	signal transducer and activator of transcription 1-alpha/beta
22.9	23.4	23.3	22.6	22.0	22.9	0.1943	0.0709	gi|635072639	clathrin light chain A isoform X4
15.6	15.7	16.9	16.9	17.3	17.1	11.0768	0.0710	gi|635061114	cullin-3 isoform X2
18.4	18.8	18.5	17.1	18.0	18.1	0.1420	0.0720	gi|635014920	nucleobindin-2 isoform X1
21.4	22.0	21.5	21.0	21.2	21.0	0.2890	0.0733	gi|635014377	reticulocalbin-1
18.7	18.0	19.4	19.7	20.0	19.6	11.7622	0.0772	gi|635120107	NEDD8-activating enzyme E1 catalytic subunit isoform X2
17.3	14.3	12.9	17.6	17.8	18.1	981.2733	0.0794	gi|635078757	eukaryotic translation initiation factor 5B isoform X2
19.2	20.3	19.0	20.6	20.3	20.6	10.1858	0.0795	gi|635034151	coactosin-like protein isoform X2
20.0	19.5	19.5	20.7	19.9	20.3	4.2761	0.0801	gi|635018123	transgelin

18.6	18.6	18.8	17.9	18.4	18.5	0.3872	0.0823	gi|635086677	nucleoredoxin
16.4	18.0	17.7	18.5	18.6	18.4	12.7464	0.0831	gi|635035330	dual specificity mitogen-activated protein kinase kinase 2
19.8	20.2	19.7	19.7	17.6	17.7	0.0268	0.0841	gi|635054464	putative hexokinase HKDC1 isoform X4
18.2	18.7	18.1	18.7	20.3	19.6	15.0106	0.0854	gi|635075763	60S ribosomal protein L7a-like
15.9	21.8	15.7	22.0	22.0	22.8	27588.1979	0.0921	gi|635034811	ATP synthase subunit delta, mitochondrial
20.0	20.3	20.3	19.9	19.5	19.9	0.4102	0.0937	gi|635042314	protein arginine N-methyltransferase 1, partial
20.8	20.7	20.7	20.2	20.5	20.6	0.5095	0.0957	gi|635139345	eukaryotic translation initiation factor 4H isoform X2
22.4	22.3	22.2	21.9	22.2	22.1	0.5674	0.0995	gi|635053631	annexin A11
22.6	21.3	20.3	22.6	22.9	22.8	23.8933	0.1041	gi|635034872	40S ribosomal protein S15 isoform X2
18.9	19.0	18.6	18.1	18.8	18.1	0.2912	0.1091	gi|635091531	vesicle-fusing ATPase isoform X5
23.4	23.6	23.3	23.6	24.1	23.7	2.2132	0.1100	gi|635134796	60S acidic ribosomal protein P1
18.6	19.1	18.5	19.0	19.2	19.5	3.2212	0.1101	gi|635017047	cysteine and histidine-rich domain-containing protein 1
23.9	24.1	23.9	23.1	23.6	23.8	0.3517	0.1139	gi|635069752	tubulin beta-4B chain
18.1	18.2	17.7	17.1	17.8	17.4	0.2930	0.1180	gi|635083230	serine/threonine-protein kinase OSR1 isoform X1
20.8	21.2	21.1	21.1	21.3	21.4	1.8129	0.1203	gi|635012825	4F2 cell-surface antigen heavy chain isoform X2
18.3	18.1	17.2	16.6	17.2	17.3	0.1505	0.1214	gi|635114870	lanosterol 14-alpha demethylase isoform X2
20.5	17.2	16.6	20.5	20.6	20.5	256.8207	0.1217	gi|635105734	rho-related GTP-binding protein RhoC
24.6	24.8	24.6	24.8	24.8	24.9	1.4366	0.1222	gi|635136214	protein disulfide-isomerase A3
24.3	24.7	24.3	24.2	24.3	24.2	0.5484	0.1263	gi|635129403	heat shock protein HSP 90-alpha
23.7	24.3	24.0	23.8	23.3	23.6	0.3727	0.1291	gi|635012066	ras-related protein Rab-1B isoform X3
20.7	21.6	22.3	22.4	22.3	22.8	8.7802	0.1292	gi|635127763	protein SET-like
15.8	15.5	18.6	17.4	20.3	19.6	324.7556	0.1338	gi|635148575	histone H3
18.1	16.2	20.0	19.9	20.0	20.6	116.9033	0.1380	gi|635034756	basigin isoform X2
22.1	22.7	21.3	23.0	22.5	23.0	6.4805	0.1405	gi|635037010	60S ribosomal protein L18a isoform X2
18.8	17.5	17.8	17.0	17.6	17.0	0.1596	0.1437	gi|635026378	transmembrane 9 superfamily member 2
18.3	17.7	20.3	15.1	14.8	18.6	0.0023	0.1446	gi|635110699	serine/arginine-rich splicing factor 10 isoform X8
17.8	16.0	21.7	21.4	21.5	21.6	1065.7533	0.1468	gi|635097595	tubulin beta-2B chain-like
23.1	23.1	22.9	23.1	23.3	23.3	1.4936	0.1505	gi|635090086	leucine-rich repeat-containing protein 59
19.2	19.3	19.0	18.5	19.1	18.9	0.4622	0.1513	gi|635014302	caprin-1
22.2	22.8	22.7	22.9	23.1	22.8	2.1787	0.1522	gi|635118739	transforming protein RhoA
19.0	19.0	18.7	19.0	19.5	19.3	2.1461	0.1523	gi|635047589	erlin-2 isoform X2
14.2	14.2	13.8	15.6	15.7	13.9	10.6143	0.1526	gi|635050183	protein EFR3 homolog A isoform X4
20.3	21.0	20.4	20.1	20.4	19.8	0.3249	0.1535	gi|635091792	glial fibrillary acidic protein isoform X3
24.8	25.2	24.9	25.1	25.3	25.2	1.6239	0.1575	gi|635117111	protein disulfide-isomerase A4
20.9	21.1	20.6	20.9	21.4	21.7	2.9523	0.1583	gi|635084397	translocon-associated protein subunit gamma
21.1	20.9	20.5	21.2	21.2	21.1	2.0758	0.1619	gi|635013758	rho GTPase-activating protein 1 isoform X3
22.3	22.8	22.3	22.1	22.1	22.2	0.5210	0.1628	gi|635015804	stress-induced-phosphoprotein 1
22.1	22.4	22.0	22.2	22.9	22.6	2.4937	0.1644	gi|635098341	protein ERGIC-53 isoform X2
15.0	20.8	14.2	20.4	20.5	19.9	3712.8437	0.1645	gi|635026932	[Protein ADP-ribosylarginine] hydrolase-like protein 1
16.0	17.4	16.8	17.3	17.5	17.6	5.1127	0.1652	gi|635079776	mannosyl-oligosaccharide glucosidase
18.1	18.6	17.9	17.6	17.7	15.5	0.0533	0.1675	gi|635034083	heat shock factor-binding protein 1

22.0	22.1	22.0	21.6	21.9	22.0	0.6618	0.1685	gi|635052960	heterogeneous nuclear ribonucleoprotein F
17.6	19.0	18.3	19.3	19.2	18.6	5.3779	0.1697	gi|635030885	eukaryotic peptide chain release factor GTP-binding subunit ERF3A isoform X3
21.8	22.7	22.2	22.0	21.8	21.5	0.3159	0.1705	gi|635128197	transmembrane emp24 domain-containing protein 10
21.3	21.3	21.4	21.7	21.3	21.8	1.9244	0.1746	gi|635103547	prefoldin subunit 2
22.9	22.4	22.5	22.1	22.4	22.5	0.5088	0.1793	gi|635014587	copper transport protein ATOX1
20.9	21.2	22.3	20.9	20.2	20.9	0.1577	0.1816	gi|635060556	60S ribosomal protein L37a
21.0	20.8	20.2	20.8	21.4	21.2	2.8329	0.1847	gi|635044167	hydroxyacyl-coenzyme A dehydrogenase, mitochondrial
18.4	18.6	17.8	18.5	18.6	19.0	2.7471	0.1879	gi|635107620	phosphoglucomutase-1 isoform X2
20.3	20.8	20.6	20.2	20.5	20.2	0.5530	0.1885	gi|635044101	inorganic pyrophosphatase 2, mitochondrial isoform X2
19.8	19.7	19.6	19.7	20.1	19.9	1.5928	0.1895	gi|635011423	cysteine--tRNA ligase, cytoplasmic isoform X3
21.7	22.1	21.4	21.9	22.3	22.1	2.2310	0.1929	gi|635147267	acidic leucine-rich nuclear phosphoprotein 32 family member E isoform X3
22.1	22.1	21.8	22.0	22.2	22.2	1.5217	0.1950	gi|635134195	electron transfer flavoprotein subunit alpha, mitochondrial isoform X1
20.6	20.7	20.1	21.0	20.8	20.6	2.0703	0.1972	gi|635066390	dynactin subunit 2 isoform X5
24.1	24.7	24.2	23.0	24.4	23.6	0.2033	0.2003	gi|635040463	60S ribosomal protein L13a isoform X2
21.5	21.9	22.0	22.1	21.9	22.1	1.7095	0.2029	gi|635111099	F-actin-capping protein subunit beta isoform X4
21.7	21.4	21.4	21.1	21.4	21.4	0.6001	0.2065	gi|635106454	calponin-3 isoform X4
20.2	20.6	20.3	20.1	20.3	20.1	0.6175	0.2088	gi|635109727	tyrosine--tRNA ligase, cytoplasmic
23.5	23.8	23.5	23.7	24.0	23.8	1.6398	0.2094	gi|635011037	transaldolase
18.0	18.3	17.6	18.1	18.6	18.4	2.3503	0.2095	gi|635131842	ubiquitin carboxyl-terminal hydrolase isozyme L5 isoform X8
21.0	21.5	21.8	22.3	21.7	21.7	2.8395	0.2098	gi|635118889	guanine nucleotide-binding protein G(i) subunit alpha-2
17.7	17.3	17.0	17.3	18.0	18.2	3.1381	0.2123	gi|635069658	39S ribosomal protein L41, mitochondrial
24.3	23.8	24.2	24.3	24.2	24.4	1.7292	0.2143	gi|635111446	EF-hand domain-containing protein D2
26.1	26.4	25.8	26.3	26.5	26.3	1.8307	0.2153	gi|635074114	annexin A1
15.8	16.3	15.5	15.8	17.2	16.5	4.4677	0.2170	gi|635061470	COP9 signalosome complex subunit 7b isoform X3
20.6	20.9	21.1	20.7	20.6	20.0	0.4100	0.2185	gi|635104778	protein S100-A13
21.5	21.7	21.2	21.2	21.3	21.2	0.6089	0.2192	gi|635036959	6-phosphogluconolactonase isoform X1
21.5	21.5	21.3	21.1	21.4	21.3	0.6967	0.2267	gi|635069550	keratin, type I cytoskeletal 18
23.1	23.5	23.3	23.0	23.1	23.2	0.6294	0.2271	gi|635144600	signal recognition particle 14 kDa protein-like
20.7	20.4	19.9	20.1	21.6	21.7	6.8070	0.2296	gi|635092278	polymerase I and transcript release factor
18.7	18.7	18.3	18.5	19.1	19.0	2.0343	0.2297	gi|635131548	importin-9 isoform X1
17.3	16.2	15.9	17.2	17.9	16.6	5.7934	0.2335	gi|635063151	ribosomal RNA small subunit methyltransferase NEP1
26.6	26.9	26.6	26.8	26.9	27.1	1.5717	0.2345	gi|635087149	profilin-1
18.0	17.7	17.9	17.6	17.8	17.6	0.6545	0.2350	gi|635071600	26S proteasome non-ATPase regulatory subunit 5 isoform X2
18.3	17.5	16.7	18.1	18.6	18.0	5.3258	0.2352	gi|635136416	EH domain-containing protein 4
17.1	17.0	17.0	17.3	18.1	17.0	2.8737	0.2354	gi|635122371	omega-amidase NIT2 isoform X2
18.8	19.3	18.6	19.0	19.4	19.3	2.1642	0.2361	gi|635115571	dihydrolipoyl dehydrogenase, mitochondrial
17.3	18.1	17.9	17.8	16.4	17.1	0.2237	0.2382	gi|635089252	TATA-binding protein-associated factor 2N isoform X2
16.7	16.5	16.0	16.7	17.2	16.5	2.4208	0.2383	gi|635043866	rap1 GTPase-GDP dissociation stimulator 1 isoform X6
17.1	17.1	16.5	16.6	16.7	16.6	0.5032	0.2409	gi|635090518	integrin beta-4 isoform X4
17.9	18.3	18.4	18.3	18.5	18.5	1.6819	0.2418	gi|635112211	leucine-rich repeat-containing protein 47
19.8	19.3	19.1	19.7	19.7	19.9	1.9884	0.2419	gi|635111691	spermidine synthase

16.2	16.2	16.1	15.7	16.3	15.8	0.5801	0.2430	gi|635036075	dynamin-2 isoform X8
17.8	18.3	17.9	17.5	17.9	17.7	0.5643	0.2430	gi|635016095	nuclear mitotic apparatus protein 1 isoform X2
18.0	17.8	17.0	17.4	17.0	17.1	0.3543	0.2431	gi|635048513	COP9 signalosome complex subunit 5
23.5	23.7	23.7	23.8	23.7	23.8	1.3451	0.2446	gi|635127077	galectin-3
19.9	20.1	20.3	20.4	20.4	20.1	1.6693	0.2483	gi|635092051	dual specificity protein phosphatase 3
16.6	15.8	14.0	13.0	15.4	13.6	0.0367	0.2514	gi|635101418	THO complex subunit 5 homolog isoform X2
18.0	18.3	18.3	18.2	18.7	18.3	1.6729	0.2528	gi|635116062	ADP-ribosylation factor 5
23.6	23.2	23.2	23.0	23.3	23.0	0.5799	0.2530	gi|635050673	elongation factor 1-delta isoform X7
18.8	19.5	18.7	17.5	19.2	18.2	0.1843	0.2549	gi|635138937	LETM1 and EF-hand domain-containing protein 1, mitochondrial isoform X4
14.8	17.9	18.4	19.5	17.8	18.9	43.3531	0.2556	gi|635035969	eukaryotic translation initiation factor 3 subunit G
21.5	20.3	20.1	20.9	21.3	21.7	5.0210	0.2557	gi|635108906	solute carrier family 2, facilitated glucose transporter member 1
16.6	16.5	17.0	15.2	16.1	16.9	0.2026	0.2558	gi|635077640	nesprin-1 isoform X17
23.8	24.7	24.3	23.6	24.0	24.1	0.3970	0.2573	gi|635057447	acyl-CoA-binding protein isoform X2
20.5	14.7	20.9	21.3	21.2	21.4	415.3555	0.2577	gi|635143551	proteolipid protein 2
20.2	20.8	20.7	20.2	20.2	20.4	0.5367	0.2596	gi|635132961	4-trimethylaminobutyraldehyde dehydrogenase
21.7	21.8	21.4	21.2	21.5	21.6	0.6145	0.2598	gi|635131556	cysteine and glycine-rich protein 1 isoform X2
22.7	22.2	22.1	21.9	22.3	22.1	0.5207	0.2603	gi|635020096	prefoldin subunit 4
22.5	23.0	22.3	22.1	22.4	22.4	0.5095	0.2607	gi|635058086	D-dopachrome decarboxylase
22.0	22.9	22.9	22.3	22.2	22.1	0.3941	0.2614	gi|635092753	60S ribosomal protein L19
21.6	21.0	20.5	21.4	21.5	21.6	2.6554	0.2626	gi|635104150	NAD(P)H-hydrate epimerase
22.6	22.6	22.4	22.4	22.4	22.5	0.7727	0.2632	gi|635054259	prosaposin isoform X3
24.6	25.0	24.8	24.8	25.2	24.9	1.5241	0.2637	gi|635100200	60S ribosomal protein L12
25.4	25.7	25.4	25.4	26.1	25.9	1.8909	0.2650	gi|635135688	60S ribosomal protein L18
18.5	18.8	18.2	14.9	18.2	18.1	0.0380	0.2665	gi|635089753	28S ribosomal protein S23, mitochondrial
24.2	24.8	24.7	25.0	24.6	24.9	1.7909	0.2672	gi|635041348	40S ribosomal protein S9
15.9	14.6	12.7	11.8	14.4	12.2	0.0261	0.2672	gi|635129357	retrotransposon-like protein 1
21.7	22.9	22.4	22.5	21.4	19.4	0.0584	0.2680	gi|635044058	ubiquitin-conjugating enzyme E2 D3 isoform X1
19.8	20.8	19.7	19.4	16.5	20.1	0.0333	0.2682	gi|635104100	hepatoma-derived growth factor isoform X2
22.2	22.6	22.2	22.1	22.2	22.0	0.5967	0.2684	gi|635072111	UV excision repair protein RAD23 homolog B
19.7	20.1	19.5	19.5	19.7	18.1	0.2061	0.2692	gi|635021686	microtubule-associated protein RP/EB family member 1 isoform X2
21.8	21.2	20.7	21.3	21.8	22.1	3.1362	0.2693	gi|635070315	surfeit locus protein 4
20.3	19.3	15.0	19.9	20.5	20.3	121.2570	0.2712	gi|635114566	NADH dehydrogenase [ubiquinone] 1 alpha subcomplex subunit 4
19.1	20.5	19.7	19.6	19.2	18.8	0.2598	0.2716	gi|635101349	RNA-binding protein EWS isoform X4
18.7	19.3	17.7	18.9	19.5	19.2	4.2529	0.2721	gi|635085400	U8 snoRNA-decapping enzyme
23.0	22.7	23.0	22.5	22.7	22.9	0.5992	0.2724	gi|635014320	CD59 glycoprotein isoform X2
18.4	18.3	18.1	17.9	18.3	17.1	0.3429	0.2726	gi|635135615	E3 ubiquitin-protein ligase NEDD4 isoform X3
19.6	19.9	19.7	19.6	20.2	20.2	1.8636	0.2726	gi|635022518	casein kinase II subunit alpha isoform X1
18.0	18.1	17.8	17.7	18.0	17.8	0.7321	0.2728	gi|635050651	nicotinate phosphoribosyltransferase isoform X4
16.6	16.5	16.4	16.5	17.2	16.7	1.9584	0.2729	REV__gi|63502	multidrug resistance-associated protein 4 isoform X4
19.3	21.2	20.9	19.7	20.3	16.5	0.0220	0.2767	gi|635035808	40S ribosomal protein S28
22.3	21.8	21.8	21.9	21.7	21.7	0.6226	0.2768	gi|635019970	vesicle-associated membrane protein-associated protein B/C isoform X3

17.3	16.8	16.3	16.9	17.3	17.5	2.7873	0.2772	gi|635021303	DDRGK domain-containing protein 1 isoform X2
19.1	18.7	18.5	19.4	18.8	19.1	2.0394	0.2779	gi|635094303	BAG family molecular chaperone regulator 2
22.0	21.9	21.9	21.8	22.1	22.4	1.6214	0.2785	gi|635093115	prohibitin
21.3	21.2	20.8	21.3	21.4	21.2	1.5801	0.2788	gi|635011196	cathepsin D
17.3	17.9	17.1	18.0	17.7	17.6	2.2415	0.2801	gi|635077547	A-kinase anchor protein 12 isoform X3
17.2	16.8	16.7	14.4	15.6	17.4	0.0800	0.2808	gi|635085230	propionyl-CoA carboxylase beta chain, mitochondrial
21.1	21.2	20.5	19.9	20.9	20.6	0.3400	0.2827	gi|635071935	hydroxysteroid dehydrogenase-like protein 2 isoform X2
19.6	20.0	19.7	19.3	19.8	19.3	0.5157	0.2844	gi|635118673	glutamine--tRNA ligase isoform X2
26.0	25.9	25.5	26.1	26.0	25.9	1.6015	0.2846	gi|635031686	fructose-bisphosphate aldolase A isoform X2
20.9	16.7	21.4	21.2	21.7	21.5	65.9331	0.2902	gi|635065838	coatomer subunit zeta-1
24.4	24.5	24.0	24.4	24.6	24.4	1.5074	0.2907	gi|635110599	chloride intracellular channel protein 4
20.1	20.7	20.2	19.8	20.3	20.1	0.5172	0.2933	gi|635065535	eukaryotic translation initiation factor 4B isoform X2
18.8	19.1	18.9	15.4	18.6	18.8	0.0443	0.2947	gi|635108952	peptidyl-prolyl cis-trans isomerase H
20.7	21.1	20.7	20.3	20.8	20.6	0.5535	0.2957	gi|635058271	26S proteasome non-ATPase regulatory subunit 14
19.0	20.8	19.5	20.3	18.1	15.5	0.0166	0.2970	gi|635067409	small nuclear ribonucleoprotein F
19.3	20.5	19.5	14.6	18.8	19.9	0.0101	0.2978	gi|635111930	vesicle-associated membrane protein 3 isoform X2
18.5	18.3	18.0	18.3	18.7	18.4	1.7714	0.2984	gi|635089821	E3 ubiquitin/ISG15 ligase TRIM25
19.0	19.2	18.2	18.3	20.2	20.7	8.9281	0.2992	gi|635040573	nuclear pore glycoprotein p62
19.9	19.8	18.4	18.8	18.7	19.0	0.2637	0.3012	gi|635084003	translocation protein SEC62
21.5	22.2	20.7	22.1	22.3	21.7	3.5939	0.3017	gi|635046265	heat shock protein HSP 90-alpha-like
17.9	16.9	16.2	17.4	18.2	17.5	4.9132	0.3026	gi|635034390	adenine phosphoribosyltransferase isoform X1
19.1	18.4	18.1	18.6	18.9	19.4	2.7237	0.3053	gi|635126957	UPF0568 protein C14orf166 homolog
22.3	22.2	21.8	22.1	22.4	22.5	1.7155	0.3056	gi|635057512	actin-related protein 3 isoform X2
24.1	24.6	24.5	23.7	24.5	24.0	0.4746	0.3069	gi|635098791	60S ribosomal protein L17
23.6	23.3	23.2	23.5	23.6	23.4	1.3960	0.3078	gi|635110833	cell division control protein 42 homolog isoform X1
21.5	22.1	22.1	22.4	21.9	22.3	1.9167	0.3088	gi|635140119	ras-related C3 botulinum toxin substrate 1 isoform X2
22.1	22.2	21.4	22.1	22.3	22.2	1.9392	0.3102	gi|635095793	protein CutA isoform X2
19.4	17.5	18.7	18.8	19.4	19.4	4.8089	0.3109	gi|635141553	DNA-(apurinic or apyrimidinic site) lyase
24.6	24.1	24.8	25.5	25.0	24.4	2.9061	0.3122	gi|635042167	40S ribosomal protein S5
16.7	16.7	16.4	16.5	16.0	16.6	0.5514	0.3123	gi|635077891	cation-independent mannose-6-phosphate receptor
25.0	25.0	24.1	25.0	25.3	24.9	2.4597	0.3128	gi|635069251	GTP-binding nuclear protein Ran isoform X1
16.6	16.2	15.5	16.2	16.7	16.5	2.4114	0.3133	gi|635032399	alpha-ketoglutarate-dependent dioxygenase FTO isoform X2
14.5	14.2	14.3	13.7	14.0	14.4	0.5344	0.3138	gi|635139626	neurosecretory protein VGF
17.2	16.7	17.4	17.6	17.4	17.2	1.8172	0.3155	gi|635112882	procollagen-lysine,2-oxoglutarate 5-dioxygenase 1
23.4	23.9	23.3	23.6	23.9	23.9	1.7767	0.3173	gi|635012352	ubiquitin-like protein fubi and ribosomal protein S30
20.6	19.6	20.3	20.3	18.3	19.6	0.1741	0.3177	gi|635123623	voltage-dependent anion-selective channel protein 1 isoform X2
17.2	16.5	15.9	16.5	17.4	17.3	3.4787	0.3217	gi|635094723	cell division cycle 5-like protein
18.2	15.5	18.5	18.2	18.6	18.8	12.2777	0.3234	gi|635090645	peptidyl-tRNA hydrolase ICT1, mitochondrial
18.1	16.5	17.5	17.7	17.8	18.2	3.4656	0.3239	gi|635017822	dihydrolipoyllysine-residue acetyltransferase component of pyruvate dehydrogenase complex, mitochondrial isoform X3
23.6	23.8	24.1	23.2	23.4	24.0	0.4863	0.3241	gi|635100971	macrophage migration inhibitory factor
22.6	23.4	23.3	23.2	23.6	23.3	1.9657	0.3243	gi|635033325	NAD(P)H dehydrogenase [quinone] 1

16.4	16.8	16.8	16.6	15.7	16.6	0.4421	0.3256	gi|635101518	splicing factor 3A subunit 1
20.0	20.5	19.9	19.8	20.1	19.7	0.5804	0.3281	gi|635079570	macrophage-capping protein
17.9	17.9	17.4	17.8	18.1	17.9	1.6232	0.3284	gi|635048956	carbonic anhydrase 2
18.6	19.3	18.6	18.8	19.5	19.2	2.1398	0.3293	gi|635057688	DNA replication licensing factor MCM6
22.4	22.6	22.4	22.3	22.0	22.6	0.6493	0.3296	gi|635141002	isocitrate dehydrogenase [NADP], mitochondrial isoform X1
25.3	25.5	25.1	25.3	25.7	25.5	1.5445	0.3298	gi|635063102	triosephosphate isomerase
23.0	24.1	23.1	24.1	23.8	23.5	2.6287	0.3299	gi|635085736	60S ribosomal protein L15
21.2	21.7	21.1	20.7	21.6	17.3	0.0340	0.3314	gi|635012608	protein phosphatase 1 regulatory subunit 14B
18.0	18.4	17.6	15.5	18.0	17.7	0.1154	0.3343	gi|635023881	6-phosphofructokinase, liver type
22.1	21.7	21.5	21.3	21.4	21.8	0.5575	0.3346	gi|635093916	cytochrome c oxidase subunit 7A2, mitochondrial
15.8	14.8	14.2	13.9	14.8	14.4	0.2682	0.3358	gi|635017740	serine/threonine-protein phosphatase 2A 65 kDa regulatory subunit A beta isoform isoform X6
17.9	19.1	15.3	19.0	18.4	18.6	17.0817	0.3360	gi|635063793	serine-threonine kinase receptor-associated protein
15.5	19.9	19.1	19.3	20.0	19.7	32.1265	0.3361	gi|635032645	anamorsin
20.0	19.9	19.1	19.7	20.1	20.1	2.2093	0.3374	gi|635073162	cytoplasmic aconitate hydratase
22.1	22.5	22.1	22.4	22.5	22.3	1.4454	0.3388	gi|635067826	cytoskeleton-associated protein 4
22.0	22.2	22.4	21.9	22.3	21.7	0.5904	0.3396	gi|635095400	lactoylglutathione lyase
22.0	21.2	21.5	20.9	21.2	21.6	0.4573	0.3401	gi|635035063	tubulin-specific chaperone A
19.6	19.9	19.5	19.5	20.3	20.1	1.8870	0.3413	gi|635126637	protein transport protein Sec23A isoform X3
15.7	15.9	15.4	17.6	15.9	15.6	4.8211	0.3421	gi|635048161	V-type proton ATPase subunit H isoform X3
18.7	18.1	17.7	18.5	18.2	18.7	2.2160	0.3437	gi|635090597	28S ribosomal protein S7, mitochondrial
20.4	20.7	20.0	20.2	20.4	18.4	0.1999	0.3440	gi|635105817	methylosome protein 50
18.2	18.0	17.1	18.0	18.1	18.4	2.4461	0.3447	gi|635027992	UPF0600 protein C5orf51 homolog isoform X2
20.0	19.9	19.5	19.3	19.8	19.5	0.5764	0.3460	gi|635110721	hydroxymethylglutaryl-CoA lyase, mitochondrial isoform X5
17.4	18.0	17.4	17.5	18.3	17.9	2.1300	0.3482	gi|635097347	transmembrane protein 14C
15.5	20.9	20.5	21.0	20.7	20.8	69.7483	0.3490	gi|635021824	microtubule-associated proteins 1A/1B light chain 3A isoform X2
22.6	23.4	23.1	23.1	23.3	23.6	1.9805	0.3498	gi|635101104	small nuclear ribonucleoprotein Sm D3
17.4	19.7	15.3	17.4	20.9	19.3	52.1427	0.3498	gi|635143942	histone H3.3-like
16.1	16.4	15.5	15.5	15.8	15.9	0.5060	0.3513	gi|635012833	nuclear RNA export factor 1
17.5	18.1	17.5	17.9	18.6	17.6	2.3668	0.3515	gi|635116076	staphylococcal nuclease domain-containing protein 1
15.3	18.3	17.3	17.5	18.2	18.0	8.9543	0.3515	gi|635055157	X-ray repair cross-complementing protein 6-like
19.3	19.2	19.4	19.0	19.4	14.9	0.0309	0.3519	gi|635012404	ADP-ribosylation factor-like protein 2 isoform X2
18.8	18.2	18.3	18.5	18.5	19.0	1.8177	0.3529	gi|635142634	pyruvate dehydrogenase E1 component subunit alpha, somatic form, mitochondrial isoform X1
23.1	23.5	23.1	24.6	23.3	23.2	3.1576	0.3530	gi|635141144	ras GTPase-activating-like protein IQGAP1
23.6	23.9	23.4	23.2	23.7	23.4	0.6121	0.3551	gi|635125783	heterogeneous nuclear ribonucleoprotein H
19.7	20.0	18.7	19.8	20.0	19.8	2.5185	0.3553	gi|635123365	lamin-B1
18.3	18.1	17.6	18.1	18.4	18.2	1.7639	0.3556	gi|635048014	DNA replication licensing factor MCM4
14.4	13.0	12.6	13.8	13.7	14.2	3.9995	0.3581	gi|635089327	HEAT repeat-containing protein 6 isoform X2
18.2	18.3	17.5	15.9	18.2	17.6	0.1709	0.3583	gi|635071031	niban-like protein 1
15.7	17.3	16.5	15.6	16.6	15.6	0.2732	0.3586	gi|635049837	eukaryotic translation initiation factor 3 subunit H
18.3	19.6	18.3	17.7	19.0	17.7	0.2352	0.3595	gi|635144372	zinc finger CCHC domain-containing protein 13
20.1	19.8	19.2	19.5	20.6	20.3	2.7178	0.3607	gi|635098317	cytochrome c-like

20.5	21.4	21.4	15.6	20.9	21.2	0.0124	0.3613	gi|635140670	60S ribosomal protein L36a
17.8	17.7	17.4	16.6	17.4	17.7	0.4507	0.3617	gi|635121790	V-type proton ATPase catalytic subunit A
21.7	21.2	21.0	21.2	22.0	21.8	2.1006	0.3636	gi|635041664	splicing factor U2AF 65 kDa subunit isoform X2
25.0	24.8	25.2	25.0	25.2	25.4	1.4474	0.3660	gi|635077852	superoxide dismutase [Mn], mitochondrial
24.9	25.6	25.3	25.3	25.5	26.1	2.1592	0.3662	gi|635018368	40S ribosomal protein S25
17.4	15.6	17.4	18.1	16.6	18.2	6.4052	0.3666	gi|635117783	prothymosin alpha-like, partial
16.7	17.2	16.0	16.5	17.3	17.5	2.8774	0.3668	gi|635065065	5-AMP-activated protein kinase subunit gamma-1 isoform X3
23.4	24.0	23.6	23.6	23.6	16.0	0.0025	0.3668	gi|635090611	small ubiquitin-related modifier 2
22.0	22.4	21.8	22.0	23.1	22.3	2.2934	0.3684	gi|635054358	GTP-binding protein SAR1a
19.7	20.5	20.2	20.1	19.6	20.0	0.5334	0.3694	gi|635117785	V-type proton ATPase 116 kDa subunit a isoform 4
18.8	19.5	18.7	19.0	18.9	17.6	0.3016	0.3700	gi|635036432	phenylalanine--tRNA ligase alpha subunit
18.7	17.6	18.6	18.6	18.7	18.6	2.2491	0.3734	gi|635119214	twinfilin-2 isoform X2
21.9	22.0	21.1	21.7	21.1	21.3	0.4608	0.3740	gi|635109522	proteasome subunit beta type-2 isoform X1
20.7	21.2	20.6	20.5	20.6	20.8	0.6228	0.3743	gi|635062354	26S proteasome non-ATPase regulatory subunit 1
18.8	18.9	18.1	15.0	18.6	18.6	0.0606	0.3751	gi|635134996	dual specificity mitogen-activated protein kinase kinase 1
18.2	17.9	17.2	17.4	18.3	21.4	17.9562	0.3753	gi|635109236	polyadenylate-binding protein 4 isoform X4
19.4	19.5	18.0	19.2	15.2	18.6	0.0484	0.3766	gi|635049944	multifunctional protein ADE2-like
21.7	22.3	21.7	21.7	21.7	21.7	0.6363	0.3767	gi|635029473	calpastatin isoform X10
15.9	15.7	15.4	15.4	16.2	16.2	1.9865	0.3768	gi|635127215	actin-related protein 10 isoform X2
20.5	15.2	20.7	20.9	20.4	20.5	62.6191	0.3789	gi|635042208	NEDD8-conjugating enzyme Ubc12
19.5	20.2	19.1	20.4	20.2	19.5	2.6257	0.3791	gi|635012854	heterogeneous nuclear ribonucleoprotein U-like protein 2 isoform X2
18.3	19.5	19.2	19.4	19.4	19.3	2.2743	0.3792	gi|635011090	CD151 antigen isoform X3
18.2	17.1	16.8	17.2	18.4	18.4	3.7722	0.3796	gi|635133944	elongation factor Tu GTP-binding domain-containing protein 1 isoform X1
21.4	21.8	21.4	21.4	21.5	21.1	0.6494	0.3805	gi|635081300	serine/arginine-rich splicing factor 7 isoform X2
15.2	18.7	17.7	18.0	18.3	18.5	10.9908	0.3809	gi|635101024	glutathione S-transferase theta-2B-like
18.7	19.4	18.6	19.0	19.3	19.1	1.8607	0.3812	gi|635117878	catenin beta-1 isoform X2
19.5	19.4	19.0	19.2	19.6	19.7	1.5824	0.3839	gi|635057049	xaa-Pro aminopeptidase 2-like
22.1	22.7	21.5	22.4	22.5	22.4	2.2332	0.3845	gi|635097418	thioredoxin domain-containing protein 5
22.7	23.2	22.9	23.1	23.1	23.1	1.3879	0.3846	gi|635080417	malate dehydrogenase, cytoplasmic
23.4	23.6	23.2	23.1	23.4	23.3	0.7132	0.3849	gi|635093656	fatty acid synthase
21.8	22.1	21.5	21.8	22.1	22.0	1.5442	0.3854	gi|635075393	heterogeneous nuclear ribonucleoprotein Q isoform X5
19.8	19.5	19.0	19.5	19.8	19.8	1.7427	0.3864	gi|635063100	ubiquitin carboxyl-terminal hydrolase 5 isoform X2
21.9	21.8	21.6	21.8	15.6	21.8	0.0101	0.3881	gi|635107390	guanine nucleotide-binding protein G(I)/G(S)/G(O) subunit gamma-12
20.1	20.2	20.0	20.0	20.2	20.4	1.3514	0.3885	gi|635019499	tumor protein D54 isoform X7
21.7	22.4	21.8	21.6	21.9	21.8	0.5938	0.3898	gi|635125625	drebrin isoform X2
23.1	23.9	23.4	23.4	23.3	23.0	0.5782	0.3902	gi|635126484	proteasome subunit alpha type-6
18.7	19.6	19.9	19.5	20.1	19.7	2.4007	0.3904	gi|635130185	pyrroline-5-carboxylate reductase 2 isoform X3
15.4	12.9	14.8	18.2	13.1	16.8	44.6468	0.3910	gi|635077375	activating signal cointegrator 1 complex subunit 3 isoform X2
15.9	18.2	18.5	18.6	18.2	18.1	6.1143	0.3917	gi|635021866	glutathione synthetase
17.1	17.1	16.3	16.8	17.2	17.4	2.0214	0.3920	gi|635074168	osteoclast-stimulating factor 1
22.6	22.7	22.5	22.3	22.7	22.5	0.7331	0.3930	gi|635121115	ruvB-like 1 isoform X1

17.2	17.0	16.9	16.5	17.2	16.9	0.6608	0.3934	gi|635094858	exportin-5 isoform X3
21.8	21.6	21.1	21.4	21.9	22.1	1.8953	0.3951	gi|635074488	cathepsin L1
19.6	20.2	19.7	19.4	20.1	19.1	0.4774	0.3959	gi|635091209	probable ATP-dependent RNA helicase DDX5
18.6	18.7	17.8	16.6	18.4	18.3	0.2509	0.3960	gi|635035790	mitochondrial import inner membrane translocase subunit TIM44
21.7	21.1	20.9	21.1	21.9	21.7	2.2168	0.3967	gi|635111902	protein DJ-1
18.6	18.8	18.7	18.3	19.1	15.6	0.0935	0.3970	gi|635136191	huntingtin-interacting protein K isoform X1
21.8	22.1	21.6	21.7	21.7	21.7	0.7381	0.3976	gi|635081068	leucine-rich PPR motif-containing protein, mitochondrial
18.0	19.3	17.6	18.2	19.3	19.0	3.6546	0.3987	gi|635110733	acyl-protein thioesterase 2
17.2	17.6	16.4	16.9	17.9	17.8	2.7915	0.4000	gi|635109176	CAAX prenyl protease 1 homolog isoform X2
19.9	19.9	19.4	19.2	19.7	19.6	0.6187	0.4002	gi|635147818	plastin-3
18.3	18.5	17.7	18.1	19.1	18.4	2.2336	0.4025	gi|635041251	U4/U6 small nuclear ribonucleoprotein Prp31
17.9	17.7	16.6	16.9	17.5	16.1	0.2863	0.4026	gi|635089348	hepatocyte nuclear factor 1-beta isoform X2
20.2	21.8	20.7	21.4	21.3	21.4	2.7974	0.4039	gi|635016307	ras-related protein Rab-6A isoform X2
23.9	23.8	23.6	23.9	23.9	23.8	1.2401	0.4040	gi|635023389	carbonyl reductase [NADPH] 1
13.1	13.5	13.0	12.9	14.0	13.8	2.1958	0.4040	gi|635117779	nuclear pore complex protein Nup205
21.2	21.0	20.5	20.9	21.2	21.3	1.6819	0.4044	gi|635035464	lon protease homolog, mitochondrial isoform X3
18.3	19.2	18.7	18.6	18.3	18.6	0.5659	0.4052	gi|635056707	C-terminal-binding protein 2 isoform X5
18.7	18.7	18.1	18.2	18.3	18.5	0.6289	0.4055	gi|635050750	poly(U)-binding-splicing factor PUF60 isoform X7
17.2	17.1	16.4	16.8	17.7	17.2	2.2320	0.4068	gi|635017207	double-strand break repair protein MRE11A isoform X3
16.7	15.7	15.4	15.9	17.0	16.3	3.0550	0.4076	gi|635111671	F-box only protein 44 isoform X2
23.9	22.9	23.1	23.3	23.9	23.8	2.0925	0.4077	gi|635065092	tubulin alpha-1B chain
17.1	17.0	16.0	16.7	17.3	17.1	2.2469	0.4078	gi|635070014	mitochondrial-processing peptidase subunit alpha isoform X3
20.5	21.0	20.7	21.1	21.8	20.4	2.4577	0.4081	gi|635107236	quinone oxidoreductase isoform X1
18.1	18.9	17.9	17.6	18.1	18.2	0.4705	0.4082	gi|635058740	cytoplasmic dynein 1 intermediate chain 2 isoform X10
24.3	24.5	23.9	24.0	24.2	24.1	0.6670	0.4091	gi|635049314	60S ribosomal protein L30
20.8	20.8	14.7	20.1	20.6	21.4	84.5305	0.4094	gi|635113191	uridine phosphorylase 1 isoform X3
21.1	21.6	21.2	21.0	21.4	20.8	0.6231	0.4099	gi|635104792	protein S100-A16
17.0	17.5	17.6	17.3	17.7	17.9	1.7417	0.4102	gi|635108558	alcohol dehydrogenase [NADP(+)] isoform X2
17.7	16.2	16.3	17.9	16.4	17.8	4.2249	0.4107	gi|635117092	cullin-1
19.8	20.1	19.9	20.0	20.2	19.9	1.3211	0.4108	gi|635120849	transmembrane protein 43
23.8	24.0	23.6	23.7	23.7	23.6	0.7482	0.4109	gi|635093872	ATP-citrate synthase
15.7	16.2	15.2	16.1	16.4	15.6	2.1973	0.4112	gi|635080598	polyribonucleotide nucleotidyltransferase 1, mitochondrial isoform X2
24.8	24.6	24.6	24.7	24.9	24.7	1.2819	0.4114	gi|635083137	40S ribosomal protein SA isoform X2
17.4	17.2	16.8	16.5	17.0	17.2	0.5835	0.4120	gi|635034878	receptor expression-enhancing protein 6 isoform X2
18.8	20.7	20.5	20.3	21.0	20.5	3.7465	0.4120	gi|635022382	small nuclear ribonucleoprotein-associated proteins B and B isoform X2
15.8	16.7	16.7	16.5	16.0	15.7	0.4578	0.4124	gi|635097427	desmoplakin isoform X2
22.0	22.7	22.0	22.3	22.4	22.6	1.7483	0.4125	gi|635043329	heterogeneous nuclear ribonucleoprotein D0 isoform X4
20.9	22.0	21.0	21.5	22.4	21.4	2.7566	0.4126	gi|635114119	chromobox protein homolog 3 isoform X2
18.5	19.0	18.6	19.2	19.0	18.6	1.6660	0.4127	gi|635034283	large neutral amino acids transporter small subunit 1 isoform X2
23.0	23.0	22.8	19.9	22.7	23.3	0.1082	0.4132	gi|635104768	protein S100-A1
18.5	18.4	17.2	18.6	18.8	18.0	2.8619	0.4136	gi|635109861	importin subunit alpha-7

17.5	15.8	15.5	16.8	16.7	17.0	3.8635	0.4140	gi|635023514	proteasome assembly chaperone 1
23.2	23.2	22.4	22.8	22.7	22.4	0.5594	0.4156	gi|635092793	proteasome subunit beta type-3
18.6	19.0	18.8	19.0	18.7	19.2	1.4111	0.4167	gi|635121124	protein transport protein Sec61 subunit alpha isoform 1
19.2	18.8	18.8	18.2	18.5	19.2	0.5015	0.4177	gi|635100049	NADH dehydrogenase [ubiquinone] flavoprotein 2, mitochondrial isoform X2
23.0	23.1	22.8	22.6	22.8	23.1	0.6845	0.4179	gi|635100588	ubiquitin-conjugating enzyme E2 L3 isoform X2
19.3	19.9	19.6	19.4	19.7	18.9	0.5644	0.4180	gi|635103354	adenylosuccinate lyase
25.4	24.7	25.0	26.1	24.8	25.4	2.3609	0.4192	gi|635139491	heat shock protein beta-1
16.8	18.1	16.6	17.5	15.2	16.7	0.1845	0.4195	gi|401316367	MHC class IB protein
25.0	25.7	25.2	25.7	25.3	25.5	1.6101	0.4200	gi|635092135	60S ribosomal protein L27
24.9	25.0	24.3	24.7	25.0	25.1	1.6152	0.4208	gi|635116380	aldo-keto reductase family 1 member B10 isoform X1
19.8	20.5	15.4	19.6	20.2	20.1	27.3598	0.4217	gi|635022032	U2 small nuclear ribonucleoprotein B
21.4	22.1	21.8	21.8	21.1	21.6	0.5395	0.4224	gi|635034209	cytochrome c oxidase subunit 4 isoform 1, mitochondrial isoform X2
16.2	16.5	15.5	15.7	17.3	16.7	3.0130	0.4233	gi|635095034	60S ribosomal protein L7-like 1 isoform X1
18.6	18.4	18.3	18.3	18.8	18.8	1.5287	0.4236	gi|635072486	aldehyde dehydrogenase X, mitochondrial isoform X5
24.8	25.9	25.3	25.4	25.8	25.7	1.9758	0.4238	gi|635128407	60S ribosomal protein L22
19.9	19.2	19.6	19.6	20.0	19.8	1.6069	0.4258	gi|635036371	ATPase ASNA1
20.5	21.0	20.5	19.8	20.9	20.4	0.4712	0.4258	gi|635011561	src substrate cortactin isoform X4
21.7	22.3	21.3	21.8	22.4	22.0	1.9477	0.4270	gi|635043343	heterogeneous nuclear ribonucleoprotein D-like isoform X2
14.5	18.4	17.3	17.6	17.5	18.3	11.1862	0.4274	gi|635093749	ketosamine-3-kinase
21.7	22.0	21.6	21.3	21.8	21.6	0.6688	0.4276	gi|635071555	ras-related protein Rab-14
18.7	19.0	18.8	15.2	19.1	18.8	0.0796	0.4277	gi|635100837	39S ribosomal protein L40, mitochondrial
22.2	22.4	21.7	22.6	22.3	22.1	1.6714	0.4281	gi|635147622	membrane-associated progesterone receptor component 1
15.7	16.8	15.9	15.6	16.0	15.9	0.4755	0.4287	gi|635143085	probable ubiquitin carboxyl-terminal hydrolase FAF-X isoform X4
17.7	17.5	16.7	17.2	17.8	17.9	2.0745	0.4297	gi|635079521	U4/U6.U5 tri-snRNP-associated protein 2 isoform X4
21.9	22.2	21.9	21.9	22.3	22.1	1.3347	0.4297	gi|635141559	purine nucleoside phosphorylase
20.0	19.7	19.0	19.8	15.8	19.7	0.0700	0.4297	gi|635096080	U6 snRNA-associated Sm-like protein LSm2
16.9	20.2	19.2	19.8	19.4	19.7	7.4414	0.4300	gi|635129109	glutaredoxin-related protein 5, mitochondrial
18.3	19.8	15.5	18.9	19.2	18.8	12.6437	0.4317	gi|635062257	thymidylate kinase isoform X1
20.7	21.4	20.8	21.3	15.8	21.0	0.0273	0.4328	gi|635023806	cystatin-B
23.7	23.7	23.5	24.0	23.7	23.5	1.3016	0.4344	gi|635040466	40S ribosomal protein S11
24.5	25.0	24.0	23.9	24.5	24.2	0.5051	0.4361	gi|635030030	40S ribosomal protein S2 isoform X1
20.8	15.1	20.9	20.2	21.3	20.4	47.9156	0.4366	gi|635011354	CD81 antigen isoform X2
19.9	20.4	19.4	19.8	20.4	20.4	2.0304	0.4367	gi|635097465	translocon-associated protein subunit alpha isoform X2
24.0	22.8	23.9	23.6	24.4	23.9	2.4304	0.4376	gi|635092357	40S ribosomal protein S26-like
22.0	22.7	22.8	22.7	22.8	22.8	1.6936	0.4383	gi|635147909	60S ribosomal protein L10
22.9	23.4	22.9	23.1	23.3	23.1	1.3857	0.4383	gi|635022540	eukaryotic translation initiation factor 6
19.7	20.1	19.6	19.7	20.1	20.1	1.4566	0.4399	gi|635128762	26S protease regulatory subunit 4
20.9	21.0	20.3	20.7	21.0	21.2	1.7155	0.4401	gi|635068717	malectin
20.1	20.4	19.6	20.1	20.4	20.3	1.6748	0.4417	gi|635124344	glucosamine-6-phosphate isomerase 1
21.3	23.0	22.9	23.1	22.7	22.9	3.0937	0.4425	gi|635024681	60S ribosomal protein L21 isoform X2
22.3	22.2	21.8	22.3	21.9	21.6	0.6346	0.4454	gi|635116813	single-stranded DNA-binding protein, mitochondrial isoform X2

23.0	22.8	22.2	22.8	23.0	22.8	1.6945	0.4456	gi|635041030	serine/threonine-protein phosphatase 2A 65 kDa regulatory subunit A alpha isoform
16.3	16.6	15.8	16.0	16.3	15.6	0.5384	0.4460	gi|635127008	fermitin family homolog 2 isoform X4
14.2	13.7	13.9	13.7	14.4	14.6	1.8511	0.4467	gi|635084478	probable ATP-dependent RNA helicase DHX36 isoform X1
21.6	22.1	21.0	22.0	21.8	21.8	1.9244	0.4469	gi|635113719	septin-7 isoform X5
24.3	24.4	24.2	24.2	24.3	24.3	0.9019	0.4472	gi|635144319	40S ribosomal protein S4, X isoform
20.0	21.5	21.8	21.9	21.9	21.1	3.3353	0.4473	gi|635105821	ATP synthase F(0) complex subunit B1, mitochondrial isoform X2
19.3	19.3	18.8	19.2	18.8	18.9	0.7031	0.4474	gi|635031741	major vault protein
18.4	18.3	18.0	17.9	18.2	18.2	0.7410	0.4476	gi|635035071	thimet oligopeptidase
23.6	21.4	21.1	18.9	21.4	22.4	0.0789	0.4477	gi|635063458	transcription elongation factor B polypeptide 2
19.9	20.4	20.1	20.1	20.3	20.5	1.4680	0.4487	gi|635038827	calpain-12 isoform X4
20.0	20.1	19.6	19.8	20.3	20.3	1.5661	0.4490	gi|635147869	plastin-3-like
19.8	20.2	20.0	20.2	19.1	19.8	0.4977	0.4499	gi|635146931	adipocyte plasma membrane-associated protein isoform X2
19.5	20.3	19.5	19.9	19.9	20.3	1.7821	0.4500	gi|635100570	mitogen-activated protein kinase 1
19.9	18.9	19.3	19.4	19.7	19.8	1.8625	0.4506	gi|635017875	neural cell adhesion molecule 1 isoform X7
18.5	19.6	19.1	20.3	17.3	16.9	0.1174	0.4509	gi|635083729	fragile X mental retardation syndrome-related protein 1 isoform X7
17.4	19.3	18.9	15.1	18.6	18.7	0.0802	0.4529	gi|635109822	histone deacetylase 1 isoform X2
20.0	20.3	19.5	20.1	20.2	20.2	1.5428	0.4530	gi|635047440	glutathione reductase, mitochondrial
19.0	18.1	18.2	18.7	19.1	18.3	1.9427	0.4530	gi|635131846	60 kDa SS-A/Ro ribonucleoprotein
23.7	24.1	23.5	23.9	24.2	23.7	1.5106	0.4538	gi|635104156	lamin
22.2	22.4	22.2	22.4	22.6	22.2	1.3230	0.4540	gi|635010939	ribonuclease inhibitor
19.1	21.0	14.3	18.8	20.7	20.0	50.5662	0.4541	gi|635069022	transmembrane emp24 domain-containing protein 2
26.5	27.0	26.4	26.7	26.8	26.9	1.4569	0.4542	gi|635071220	78 kDa glucose-regulated protein
22.6	23.2	22.6	22.7	23.2	23.2	1.6783	0.4572	gi|635139493	14-3-3 protein gamma
25.5	26.3	26.0	26.0	26.3	26.1	1.5690	0.4588	gi|635011074	60S acidic ribosomal protein P2
17.1	17.8	16.8	17.1	18.1	17.6	2.2154	0.4589	gi|635041596	isochorismatase domain-containing protein 2, mitochondrial isoform X2
16.6	16.6	16.4	16.4	16.8	15.3	0.4310	0.4590	gi|635110799	ephrin type-B receptor 2 isoform X4
21.0	21.1	20.7	21.1	21.0	21.0	1.2649	0.4595	gi|635091312	26S protease regulatory subunit 8 isoform X2
20.5	20.5	20.2	19.6	20.5	20.4	0.5531	0.4613	gi|635062601	peptidyl-prolyl cis-trans isomerase FKBP4
19.1	19.9	19.7	19.6	20.5	19.5	2.1097	0.4615	gi|635128386	SRA stem-loop-interacting RNA-binding protein, mitochondrial
19.9	20.2	19.6	19.7	20.3	20.4	1.6567	0.4630	gi|635122445	oxygen-dependent coproporphyrinogen-III oxidase, mitochondrial
18.2	18.2	16.9	17.8	18.0	18.8	2.6754	0.4640	gi|635042286	SUMO-activating enzyme subunit 1
22.0	22.3	21.2	21.4	21.8	21.5	0.5016	0.4645	gi|635077868	acetyl-CoA acetyltransferase, cytosolic
21.3	22.3	22.0	21.2	22.0	21.5	0.4908	0.4661	gi|635105802	ras-related protein Rap-1A
20.7	19.6	20.2	20.2	20.7	20.4	1.9127	0.4668	gi|635141900	polyadenylate-binding protein 2
24.4	24.9	24.2	24.2	24.1	24.6	0.6151	0.4669	gi|635022989	superoxide dismutase [Cu-Zn]
21.9	22.5	22.2	21.8	22.2	22.0	0.6979	0.4672	gi|635012684	ubiquitin thioesterase OTUB1
18.8	20.9	20.5	20.9	20.2	20.7	3.5685	0.4692	gi|635123064	receptor expression-enhancing protein 5
18.0	18.2	18.1	17.9	18.2	18.0	0.8162	0.4693	gi|635101391	AP-1 complex subunit beta-1 isoform X8
24.5	25.1	24.6	24.8	25.0	24.8	1.3949	0.4702	gi|635119712	ADP-ribosylation factor 4 isoform X1
15.9	16.9	15.3	15.5	17.3	17.0	3.7302	0.4709	gi|635134118	WD repeat-containing protein 61 isoform X4
17.9	18.2	17.6	17.7	18.4	18.4	1.7350	0.4725	gi|635054161	prolyl 4-hydroxylase subunit alpha-1 isoform X2

20.5	21.2	20.1	20.3	20.6	20.2	0.5424	0.4729	gi|635040292	apoptosis regulator BAX isoform X2
19.7	19.3	14.1	19.3	19.3	18.8	26.7126	0.4730	gi|635084636	profilin-2
22.8	23.2	22.9	23.1	23.0	22.1	0.5299	0.4737	gi|635097855	60S ribosomal protein L23
20.1	20.6	20.0	19.5	20.4	20.0	0.5469	0.4749	gi|635033096	splicing factor 3B subunit 3
22.0	23.8	23.1	22.2	23.3	21.7	0.2666	0.4760	gi|635088778	60S ribosomal protein L23a
20.9	21.5	20.9	21.2	21.2	21.3	1.4140	0.4762	gi|635124842	annexin A6
25.5	26.4	25.8	25.6	26.1	25.1	0.5009	0.4765	gi|635137765	ubiquitin carboxyl-terminal hydrolase isozyme L1
24.7	25.3	24.7	24.8	24.6	24.8	0.6816	0.4765	gi|635103783	transgelin-2
21.3	22.1	20.2	21.6	21.7	21.6	2.7512	0.4769	gi|635138284	dihydropteridine reductase
18.3	18.3	18.8	18.2	17.4	18.8	0.4355	0.4775	gi|635120639	proline-rich transmembrane protein 3 isoform X7
20.2	21.9	21.8	20.9	21.1	20.5	0.3548	0.4791	gi|635071304	proteasome subunit beta type-7 isoform X2
19.9	22.6	19.8	19.1	20.7	20.1	0.1543	0.4805	gi|635125723	heterogeneous nuclear ribonucleoprotein A/B
18.3	18.0	16.8	17.8	18.2	18.2	2.3919	0.4814	gi|635062002	NADH dehydrogenase [ubiquinone] 1 alpha subcomplex subunit 10, mitochondrial isoform X2
18.7	18.6	18.0	18.4	18.7	18.7	1.5413	0.4827	gi|635142414	pirin isoform X2
24.4	24.5	23.9	24.7	24.6	24.1	1.6246	0.4831	gi|635104919	protein S100-A11
20.4	20.0	19.7	19.8	19.9	19.8	0.6664	0.4832	gi|635143818	ragulator complex protein LAMTOR3
14.3	16.8	15.8	15.7	16.7	16.3	3.9382	0.4852	gi|635043129	nucleoporin p54 isoform X2
20.3	20.0	19.8	19.9	20.2	19.3	0.5766	0.4855	gi|635085677	ras-related protein Rab-5A
22.0	22.5	22.4	22.4	22.6	22.3	1.3616	0.4857	gi|635074250	phosphoserine aminotransferase
24.7	25.1	24.5	24.4	24.7	24.7	0.7037	0.4858	gi|635074371	heterogeneous nuclear ribonucleoprotein K
23.4	23.6	23.4	23.4	23.7	23.7	1.2539	0.4868	gi|635126083	proteasome activator complex subunit 1
20.1	19.8	19.7	19.7	19.6	19.9	0.7754	0.4870	gi|635112427	guanine nucleotide-binding protein G(I)/G(S)/G(T) subunit beta-1
17.6	18.0	14.9	17.1	17.8	17.8	5.6935	0.4872	gi|635062795	fructose-2,6-bisphosphatase TIGAR
21.5	22.6	22.1	20.8	22.3	21.9	0.3793	0.4876	gi|635048018	ubiquitin-conjugating enzyme E2 variant 2 isoform X2
20.9	20.1	19.7	20.8	21.2	19.9	2.4258	0.4878	gi|635112856	heterogeneous nuclear ribonucleoprotein R
18.6	18.7	19.0	17.7	16.9	19.8	0.2185	0.4885	gi|635081045	prolyl endopeptidase-like isoform X3
20.0	20.4	19.7	19.9	20.6	20.2	1.5896	0.4890	gi|635042913	MOB kinase activator 1B isoform X5
18.7	20.1	19.5	19.9	19.7	14.5	0.0420	0.4896	gi|635105075	26S proteasome non-ATPase regulatory subunit 4
16.5	16.6	15.6	16.4	16.2	17.1	2.0457	0.4897	gi|635056671	phospholysine phosphohistidine inorganic pyrophosphate phosphatase isoform X3
21.6	21.8	21.4	21.5	21.6	21.3	0.7832	0.4908	gi|635092401	peptidyl-prolyl cis-trans isomerase FKBP10 isoform X2
22.3	24.2	24.0	23.9	23.9	24.2	2.8744	0.4916	gi|635087773	60S ribosomal protein L26
17.9	19.4	18.9	19.3	19.6	18.5	2.6092	0.4922	gi|635116977	zyxin isoform X3
22.4	22.5	22.3	21.8	22.5	22.4	0.6653	0.4923	gi|635089088	26S proteasome non-ATPase regulatory subunit 11
17.9	17.9	17.5	17.5	18.1	18.5	1.6993	0.4930	gi|635123387	protein PRRC1
26.4	27.1	26.8	26.5	26.6	26.6	0.6965	0.4935	gi|635094763	heat shock protein HSP 90-beta
17.1	17.3	16.5	16.9	17.7	17.0	1.8056	0.4935	gi|635086178	lipoma-preferred partner
15.3	16.3	15.5	15.0	15.2	15.9	0.4799	0.4945	gi|635035258	hepatoma-derived growth factor-related protein 2 isoform X2
21.7	21.9	21.5	21.7	21.8	22.0	1.3004	0.4946	gi|635107295	cystathionine gamma-lyase isoform X1
18.2	18.2	17.7	18.0	18.2	18.2	1.3887	0.4963	gi|635081726	BRCA1-A complex subunit BRE isoform X4
21.0	21.3	20.4	21.2	21.2	20.9	1.5728	0.4963	gi|401316342	MHC class IA protein, partial
18.5	18.9	18.6	17.9	18.8	18.6	0.6087	0.4963	gi|635064742	twinfilin-1 isoform X2

19.2	19.2	19.0	19.0	19.4	19.2	1.2810	0.4968	gi|635101743	developmentally-regulated GTP-binding protein 1
25.9	26.5	26.4	26.4	26.3	26.6	1.4321	0.4969	gi|635093945	elongation factor 1-alpha 1
20.8	20.1	19.8	20.7	20.4	20.3	1.7000	0.4983	gi|635032841	aspartate aminotransferase, mitochondrial
15.6	14.9	13.1	14.5	16.2	14.9	4.5269	0.4986	gi|635036337	lysosomal alpha-mannosidase isoform X2
23.0	22.9	22.1	22.8	23.0	22.8	1.6236	0.4995	gi|635113514	proteasome subunit alpha type-2
20.3	20.8	19.8	19.9	21.1	21.0	2.2650	0.4995	gi|635033351	cytochrome b5 type B
18.7	18.1	16.9	17.8	18.1	15.9	0.2291	0.5003	gi|635073145	dnaJ homolog subfamily A member 1
17.5	18.0	17.4	17.4	18.0	18.1	1.6305	0.5015	gi|635032166	dnaJ homolog subfamily A member 2
18.8	18.9	18.1	18.4	18.0	18.8	0.5774	0.5028	gi|635028735	28S ribosomal protein S27, mitochondrial isoform X2
18.5	18.9	18.6	18.6	19.0	18.7	1.2924	0.5029	gi|635108193	thioredoxin domain-containing protein 12
18.1	17.7	17.6	17.5	17.5	17.9	0.7039	0.5036	gi|635058269	T-box brain protein 1
16.3	16.6	15.4	15.7	17.1	16.7	2.5136	0.5051	gi|635116176	transportin-3 isoform X2
19.0	19.4	19.0	18.4	19.2	19.0	0.6082	0.5059	gi|635031591	eukaryotic translation initiation factor 3 subunit C
24.4	25.0	24.5	25.1	24.8	24.6	1.5592	0.5062	gi|635099823	myosin regulatory light chain 12A isoform X2
23.1	23.1	23.5	22.9	23.2	23.1	0.7770	0.5075	gi|635080453	T-complex protein 1 subunit delta
16.8	16.8	16.3	16.1	16.6	16.6	0.6742	0.5076	gi|635084309	elongation factor G, mitochondrial
19.1	19.0	18.9	18.9	19.2	19.1	1.1884	0.5083	gi|635059456	unconventional myosin-Ib isoform X8
19.8	19.8	19.7	19.5	20.1	20.2	1.4378	0.5085	gi|635049029	2,4-dienoyl-CoA reductase, mitochondrial
20.2	20.7	19.6	20.1	20.1	19.6	0.5553	0.5090	gi|635115016	sorcin isoform X3
15.5	15.3	14.5	15.0	15.8	15.3	1.9001	0.5095	gi|635044718	heat shock 70 kDa protein 4L
18.1	20.3	19.4	19.4	19.7	15.3	0.0750	0.5100	gi|635047181	serine/threonine-protein phosphatase 2A 55 kDa regulatory subunit B alpha isoform
19.6	19.1	19.5	19.3	19.5	20.0	1.4942	0.5101	gi|635100580	stromal cell-derived factor 2-like protein 1
19.1	18.6	17.7	18.2	19.1	19.1	2.2693	0.5112	gi|635021116	long-chain-fatty-acid--CoA ligase 4-like
20.3	21.6	20.7	21.2	21.9	20.7	2.3444	0.5120	gi|635060667	actin-related protein 2/3 complex subunit 2 isoform X4
21.6	22.1	21.7	21.5	22.1	21.2	0.6034	0.5121	gi|635068565	phosphatidylethanolamine-binding protein 1 isoform X2
23.5	23.5	23.1	23.2	23.2	23.4	0.7860	0.5123	gi|635102530	aconitate hydratase, mitochondrial isoform X2
21.3	21.3	20.6	20.9	21.6	21.4	1.6183	0.5146	gi|635137115	enoyl-CoA hydratase, mitochondrial
19.8	20.4	21.1	20.3	19.3	20.6	0.4088	0.5150	gi|635053864	voltage-dependent anion-selective channel protein 2 isoform X2
11.4	14.1	11.2	11.5	14.5	13.4	7.9281	0.5158	gi|635133362	HEAT repeat-containing protein 1
19.8	19.9	18.2	19.5	16.7	19.5	0.1733	0.5166	gi|635059722	peptidyl-prolyl cis-trans isomerase-like 3
21.4	21.6	21.2	21.5	21.6	21.4	1.2151	0.5179	gi|635098466	asparagine--tRNA ligase, cytoplasmic
25.1	24.8	24.1	23.9	24.8	24.5	0.5334	0.5188	gi|635107875	cofilin-1
22.1	22.2	21.7	21.7	21.8	22.0	0.7424	0.5195	gi|635113469	biliverdin reductase A isoform X2
21.1	19.8	21.3	20.9	20.9	21.6	2.3806	0.5199	gi|635014254	CD44 antigen isoform X19
17.2	17.4	17.4	17.3	15.5	17.7	0.3267	0.5207	gi|635143828	melanoma-associated antigen D2
24.6	24.4	24.0	24.4	24.6	24.4	1.3457	0.5207	gi|635137470	pyruvate kinase PKM
17.3	20.9	18.4	17.8	21.6	20.5	12.4037	0.5217	gi|635096862	histone H3.1
23.6	24.1	23.6	23.9	23.9	23.9	1.2711	0.5224	gi|635023078	T-complex protein 1 subunit theta
19.8	19.1	16.2	18.5	19.3	19.7	6.4093	0.5236	gi|635130161	poly [ADP-ribose] polymerase 1
24.3	24.6	24.1	24.4	24.7	24.4	1.3363	0.5246	gi|635123914	stress-70 protein, mitochondrial
23.4	23.4	23.5	23.4	23.6	23.5	1.0977	0.5250	gi|635080147	poly(rC)-binding protein 1

21.0	21.6	21.1	21.2	21.4	21.4	1.3610	0.5253	gi|635040861	electron transfer flavoprotein subunit beta
22.0	23.5	22.7	23.0	23.3	22.9	2.0364	0.5263	gi|635057348	60S ribosomal protein L34
21.3	22.0	21.6	21.4	21.6	21.4	0.6944	0.5268	gi|635039002	26S protease regulatory subunit 6B
18.6	18.3	18.2	17.9	18.3	18.5	0.7167	0.5280	gi|635146583	vesicle-associated membrane protein 7 isoform X2
19.0	19.8	18.6	18.6	19.4	18.6	0.5001	0.5293	gi|635060496	fibronectin isoform X16
26.0	26.7	26.4	26.4	26.5	26.5	1.3991	0.5293	gi|635116390	aldo-keto reductase family 1 member B10-like isoform X3
15.5	15.4	14.3	15.1	15.3	15.6	1.8851	0.5294	gi|635090328	ubiquitin-conjugating enzyme E2 O isoform X1
20.8	20.8	21.9	21.5	21.1	21.7	1.9057	0.5309	gi|635128109	epididymal secretory protein E1
15.0	16.5	15.3	15.0	16.5	16.7	2.9476	0.5312	gi|635121922	mycophenolic acid acyl-glucuronide esterase, mitochondrial
22.6	23.4	22.8	22.7	24.2	23.1	2.2105	0.5318	gi|635139904	cytochrome P450 3A5 isoform X1
19.2	19.3	19.3	18.8	19.4	19.2	0.7568	0.5323	gi|635116226	ubiquitin-conjugating enzyme E2 H isoform X2
19.9	20.3	19.8	19.4	20.2	19.8	0.6245	0.5324	gi|635070795	protein SET
20.6	21.5	20.2	19.5	20.4	21.1	0.3881	0.5324	gi|635029061	dihydrofolate reductase isoform X2
22.0	22.2	19.5	21.8	21.8	21.8	3.8323	0.5326	gi|635146703	B-cell receptor-associated protein 31
19.9	19.7	19.3	19.9	19.9	19.5	1.3984	0.5337	gi|635066493	elongation factor Ts, mitochondrial isoform X2
16.6	17.1	15.9	16.7	17.0	16.7	1.7602	0.5338	gi|635129880	serine palmitoyltransferase 2
23.2	24.4	23.7	23.4	23.6	23.4	0.5701	0.5344	gi|635135319	tropomyosin alpha-1 chain isoform X6
22.6	22.9	22.4	22.4	22.6	22.5	0.7959	0.5348	gi|635096172	spliceosome RNA helicase DDX39B isoform X1
21.7	21.8	21.5	21.5	22.3	21.8	1.5015	0.5349	gi|635063148	prohibitin-2
15.9	16.4	15.2	15.6	16.2	16.7	2.0546	0.5351	gi|635013032	cleavage and polyadenylation specificity factor subunit 7 isoform X4
18.4	18.6	18.4	18.3	18.6	18.3	0.8165	0.5362	gi|635030178	enoyl-CoA delta isomerase 1, mitochondrial
14.5	16.2	15.3	15.4	17.1	15.1	3.3977	0.5363	gi|635057035	delta-1-pyrroline-5-carboxylate synthase
21.3	21.6	21.0	21.3	21.7	21.4	1.3913	0.5365	gi|635072681	talin-1 isoform X2
16.8	16.8	16.7	16.6	17.0	16.2	0.6860	0.5367	gi|635088772	ras-related protein Rab-34 isoform X6
26.8	27.5	27.1	27.1	27.4	27.4	1.4307	0.5382	gi|635116374	aldose reductase
20.1	20.9	20.5	20.2	20.2	20.6	0.6691	0.5406	gi|635108546	nuclear autoantigenic sperm protein isoform X2
15.6	15.9	14.1	15.0	16.3	15.7	2.7757	0.5407	gi|635073860	pumilio domain-containing protein KIAA0020 homolog
15.3	16.2	14.6	15.2	16.4	15.7	2.3608	0.5408	gi|635092121	interferon-induced 35 kDa protein
20.7	21.0	19.8	19.8	20.4	20.4	0.5151	0.5414	gi|635123134	transmembrane emp24 domain-containing protein 7
18.8	19.5	19.0	18.3	19.2	19.1	0.5774	0.5425	gi|635015806	neuroblast differentiation-associated protein AHNAK
23.1	23.7	23.3	24.0	23.6	23.2	1.5503	0.5425	gi|635035458	60S ribosomal protein L36
18.2	17.5	16.6	17.3	18.1	17.8	2.2492	0.5431	gi|635039167	heterogeneous nuclear ribonucleoprotein U-like protein 1 isoform X3
20.1	22.3	20.6	21.6	22.1	20.8	3.2795	0.5435	gi|635078256	proteasome subunit beta type-1
21.1	21.6	20.9	21.0	21.4	20.5	0.6071	0.5455	gi|635086547	transferrin receptor protein 1 isoform X3
14.8	14.6	14.0	14.3	14.6	13.8	0.5956	0.5464	gi|635066271	prolow-density lipoprotein receptor-related protein 1
24.9	25.2	25.0	25.3	25.4	24.8	1.3642	0.5470	gi|635048663	60S ribosomal protein L7
20.3	18.5	18.2	19.7	19.5	19.2	2.8014	0.5473	gi|635140201	fascin
15.2	15.9	15.3	15.0	16.2	16.1	2.0265	0.5474	gi|635024086	formimidoyltransferase-cyclodeaminase isoform X2
20.2	22.0	21.9	21.8	21.9	21.5	2.4082	0.5477	gi|635038369	calpain small subunit 1
17.8	19.2	17.2	18.0	19.1	18.5	2.7713	0.5496	gi|635055363	transmembrane 9 superfamily member 3
19.1	18.7	18.0	18.7	16.5	18.9	0.2932	0.5504	gi|635018164	sodium/potassium-transporting ATPase subunit gamma isoform X3

26.5	27.0	27.0	27.1	26.9	26.9	1.3146	0.5505	gi|635108560	peroxiredoxin-1
20.0	19.9	19.5	19.9	20.1	19.7	1.3155	0.5510	gi|635068288	aldehyde dehydrogenase, mitochondrial isoform X2
18.0	17.7	17.3	17.3	17.6	17.6	0.7077	0.5512	gi|635049530	V-type proton ATPase subunit C 1
22.5	22.9	22.1	22.6	22.9	22.4	1.4827	0.5519	gi|635060532	X-ray repair cross-complementing protein 5
20.0	20.0	18.3	20.2	19.8	19.5	2.4388	0.5521	gi|635111222	succinate dehydrogenase [ubiquinone] iron-sulfur subunit, mitochondrial
13.5	15.3	12.9	13.1	16.0	14.7	5.1043	0.5537	gi|635081531	protein FAM98A
21.7	21.8	21.0	21.3	21.6	20.9	0.6294	0.5540	gi|635101098	gamma-glutamyltranspeptidase 1 isoform X2
27.3	27.5	27.1	27.3	27.7	27.3	1.2961	0.5546	gi|635111848	alpha-enolase
20.6	21.1	20.7	20.6	20.5	20.9	0.7500	0.5556	gi|635068162	sarcoplasmic/endoplasmic reticulum calcium ATPase 2 isoform X3
20.4	20.5	20.2	20.1	20.7	20.0	0.7225	0.5556	gi|635028747	transportin-1 isoform X2
21.8	22.0	21.7	21.7	21.9	21.6	0.8140	0.5576	gi|635018272	coatomer subunit delta isoform X1
24.9	25.4	25.0	24.9	26.5	25.0	2.1740	0.5582	gi|635120083	40S ribosomal protein S20
20.6	20.6	20.3	19.9	19.1	21.2	0.4018	0.5583	gi|635092779	LIM and SH3 domain protein 1 isoform X1
21.9	22.1	22.1	22.0	22.1	22.3	1.2027	0.5585	gi|635139733	guanine nucleotide-binding protein G(I)/G(S)/G(T) subunit beta-2
21.3	22.0	21.6	21.6	21.6	21.2	0.7125	0.5602	gi|635117452	GTP-binding protein Rheb isoform X2
20.6	20.3	19.9	19.9	20.3	20.2	0.7034	0.5629	gi|635098769	3-ketoacyl-CoA thiolase, mitochondrial
22.3	22.5	21.7	22.0	22.1	21.9	0.6910	0.5641	gi|635033323	26S proteasome non-ATPase regulatory subunit 7
17.8	18.4	17.4	17.7	18.4	18.1	1.6473	0.5642	gi|635086757	pre-mRNA-processing-splicing factor 8
21.3	21.7	21.2	21.4	21.4	21.1	0.7835	0.5648	gi|635127484	C-1-tetrahydrofolate synthase, cytoplasmic isoform X1
21.0	22.2	21.8	21.8	21.9	22.0	1.6624	0.5651	gi|635132036	actin-related protein 2/3 complex subunit 5
17.4	17.3	17.4	18.0	17.4	15.0	0.2669	0.5664	gi|635030003	hydroxyacylglutathione hydrolase, mitochondrial isoform X3
19.3	20.9	19.8	20.5	20.0	20.5	2.1054	0.5666	gi|635032481	cleavage and polyadenylation specificity factor subunit 5
13.9	14.3	13.6	13.6	14.8	14.1	1.7888	0.5668	gi|635088898	ARF GTPase-activating protein GIT1 isoform X1
19.1	19.0	20.8	20.0	19.5	20.9	2.8571	0.5677	gi|635052355	ras-related protein Rab-18 isoform X3
20.6	21.6	20.9	21.0	21.2	21.6	1.5815	0.5679	gi|635038809	eukaryotic translation initiation factor 3 subunit K isoform X1
19.5	17.7	17.9	18.9	18.4	18.8	2.2737	0.5682	gi|635079986	sepiapterin reductase
17.3	17.6	16.7	16.9	17.9	17.6	1.7526	0.5683	gi|635090444	signal recognition particle subunit SRP68 isoform X1
18.1	18.6	18.1	18.1	18.0	18.3	0.7556	0.5696	gi|635138602	wolframin
20.0	21.3	20.5	20.8	20.7	21.1	1.7387	0.5698	gi|635143925	ubiquilin-2
21.4	22.2	21.9	21.6	21.6	21.9	0.7038	0.5701	gi|635134829	acidic leucine-rich nuclear phosphoprotein 32 family member A
21.6	20.9	20.9	21.1	21.5	21.3	1.4272	0.5704	gi|635014368	eukaryotic translation initiation factor 3 subunit M
21.7	21.5	21.2	21.3	21.9	21.6	1.3889	0.5705	gi|635046283	alcohol dehydrogenase class-3
24.6	25.1	24.8	24.8	25.0	25.1	1.2995	0.5714	gi|635016430	40S ribosomal protein S3
19.8	19.9	19.8	19.7	19.9	20.0	1.1435	0.5722	gi|635043400	protein transport protein Sec31A isoform X17
17.9	19.0	18.4	18.2	18.9	19.0	1.8005	0.5732	gi|635094971	inactive tyrosine-protein kinase 7 isoform X3
18.4	17.9	18.0	18.7	17.8	18.3	1.5559	0.5739	gi|635066035	extended synaptotagmin-1 isoform X2
18.7	18.2	17.3	18.4	18.4	18.1	1.7691	0.5742	gi|635060726	cell differentiation protein RCD1 homolog isoform X2
19.4	19.0	18.8	19.0	19.2	19.4	1.3434	0.5749	gi|635078586	cytochrome c oxidase subunit 5B, mitochondrial
23.4	23.9	23.1	23.4	23.8	23.6	1.4548	0.5758	gi|635082905	40S ribosomal protein S7
25.1	25.0	25.0	24.7	25.1	25.0	0.8390	0.5763	gi|635076368	40S ribosomal protein S12
21.7	22.6	22.1	21.4	22.2	22.1	0.6009	0.5766	gi|635142479	histone-binding protein RBBP7 isoform X2

20.9	21.7	21.6	21.3	21.2	21.3	0.6922	0.5768	gi|635127684	eukaryotic translation initiation factor 2 subunit 1
21.1	21.6	21.4	20.9	21.8	22.1	1.6874	0.5771	gi|635115790	caveolin-1 isoform X2
19.4	20.1	19.5	19.6	20.0	19.9	1.4258	0.5799	gi|635017590	acetyl-CoA acetyltransferase, mitochondrial
18.4	17.4	16.8	18.5	18.2	17.1	2.3821	0.5799	gi|635051365	prostaglandin F synthase 1-like
22.3	22.8	22.0	22.3	22.9	22.3	1.5352	0.5804	gi|635127211	proteasome subunit alpha type-3
22.0	22.5	22.0	22.0	22.1	22.2	0.8126	0.5805	gi|635036777	tropomyosin alpha-4 chain isoform X2
15.9	17.2	15.3	15.9	17.3	16.5	2.5950	0.5812	gi|635077491	E3 UFM1-protein ligase 1 isoform X2
14.7	15.5	14.2	14.4	15.5	15.5	2.0615	0.5816	gi|635101879	F-box only protein 7 isoform X3
21.6	22.6	21.5	22.1	22.5	21.9	1.7625	0.5823	gi|635120395	ADP-ribosylation factor-like protein 8B
24.2	24.5	23.9	24.1	24.5	24.4	1.3338	0.5833	gi|635134960	60S ribosomal protein L4
21.7	22.2	20.1	21.5	21.7	21.9	2.3899	0.5833	gi|51104726	cytochrome oxidase subunit II, partial (mitochondrion)
13.8	14.6	14.2	13.6	14.3	14.1	0.6632	0.5835	gi|635127473	nesprin-2 isoform X9
24.7	24.9	24.4	24.7	24.9	24.8	1.2175	0.5836	gi|635119400	transketolase
16.9	15.6	14.1	15.4	17.5	15.7	4.2153	0.5836	gi|635110233	replication protein A 32 kDa subunit
17.7	17.3	16.9	17.5	16.6	17.2	0.6217	0.5848	gi|635139851	DNA replication licensing factor MCM7 isoform X1
23.9	24.3	23.8	23.9	23.9	23.9	0.8257	0.5850	gi|635066769	T-complex protein 1 subunit beta isoform X1
20.3	20.4	19.8	20.0	20.5	20.4	1.4109	0.5850	gi|635036020	hsp90 co-chaperone Cdc37
22.4	22.6	22.0	22.2	22.8	22.4	1.4179	0.5852	gi|635026294	importin-5
21.6	22.5	24.0	22.8	24.8	22.3	4.1144	0.5863	gi|635147201	histone H2B type 2-F isoform X2
20.6	22.0	21.9	20.3	21.8	21.3	0.4144	0.5868	gi|635088740	fructose-bisphosphate aldolase C isoform X2
18.6	19.9	19.3	19.3	19.6	19.6	1.7156	0.5876	gi|635084230	importin subunit alpha-3
24.4	25.0	24.9	24.6	24.9	25.3	1.4221	0.5880	gi|635086799	14-3-3 protein epsilon isoform X1
20.8	21.2	20.3	20.6	21.0	20.1	0.6200	0.5882	gi|635126915	glycogen phosphorylase, liver form isoform X2
17.2	17.5	16.2	16.9	17.7	17.2	1.8433	0.5890	gi|635015830	nuclear pore complex protein Nup160
15.6	16.1	15.1	15.3	16.5	15.9	1.8245	0.5896	gi|635070647	torsin-1A
24.0	23.9	23.4	23.6	23.9	24.2	1.3994	0.5902	gi|635053733	40S ribosomal protein S24 isoform X10
19.1	20.0	19.8	19.3	19.7	19.4	0.6688	0.5902	gi|635073970	cAMP-dependent protein kinase catalytic subunit gamma
17.0	16.9	16.7	16.6	16.9	16.9	0.8230	0.5904	gi|635145965	fragile X mental retardation protein 1 isoform X9
19.5	21.8	19.4	20.1	19.6	19.6	0.3395	0.5907	gi|635113388	synaptobrevin homolog YKT6 isoform X2
20.7	19.6	19.0	19.9	20.4	19.9	2.0004	0.5912	gi|635116405	bisphosphoglycerate mutase
26.3	27.0	26.5	26.6	26.4	26.3	0.7202	0.5927	gi|635104814	protein S100-A6
19.8	21.2	20.7	20.6	20.5	19.8	0.5165	0.5940	gi|635125681	transmembrane emp24 domain-containing protein 9
22.8	23.1	20.0	22.4	23.3	22.1	4.1303	0.5945	gi|635125079	60S ribosomal protein L3-like
17.9	17.5	16.3	17.8	18.5	16.6	2.6416	0.5954	gi|635051852	ras suppressor protein 1 isoform X4
24.0	23.8	23.5	23.5	23.6	24.0	0.7679	0.5956	gi|635072402	acidic leucine-rich nuclear phosphoprotein 32 family member B isoform X2
22.2	22.8	22.4	22.3	22.5	22.2	0.7728	0.5962	gi|635135038	ras-related protein Rab-11A
24.4	25.1	24.7	24.9	24.9	24.8	1.3189	0.5966	gi|635067688	endoplasmin
20.3	21.7	20.2	20.4	21.0	19.6	0.4424	0.5971	gi|635093564	THO complex subunit 4
20.9	21.4	21.5	21.2	21.4	21.6	1.3739	0.5974	gi|635087097	proteasome subunit beta type-6 isoform X1
20.3	20.4	18.8	19.1	19.6	19.8	0.4848	0.5977	gi|635103362	NADH-cytochrome b5 reductase 3
21.2	19.0	19.0	19.3	19.3	19.2	0.3777	0.5992	gi|635079368	copine-2-like

20.3	20.3	19.3	19.1	19.9	20.1	0.5488	0.5993	gi|635124885	ras GTPase-activating protein-binding protein 1 isoform X2
20.4	20.2	18.8	19.4	20.3	20.8	2.3268	0.6008	gi|635081584	protein dpy-30 homolog isoform X3
21.8	22.3	22.0	21.7	22.1	22.0	0.7855	0.6013	gi|635040300	ruvB-like 2 isoform X1
21.8	22.1	21.7	21.6	21.9	21.9	0.8122	0.6018	gi|635062203	septin-2
22.3	22.6	22.2	22.3	22.4	22.6	1.2374	0.6022	gi|635122198	60S ribosomal protein L24
17.9	18.6	18.2	18.2	18.4	18.3	1.2790	0.6023	gi|635052760	presequence protease, mitochondrial
21.1	22.0	20.5	21.0	21.8	21.7	1.9492	0.6026	gi|635048336	ras-related protein Rab-2A isoform X1
20.5	20.1	19.7	20.0	20.5	20.2	1.4408	0.6029	gi|635013418	FACT complex subunit SSRP1
21.4	22.0	21.5	21.6	21.8	21.8	1.2585	0.6033	gi|635054135	annexin A7 isoform X2
21.3	22.1	20.5	20.9	21.7	20.0	0.4142	0.6042	gi|635109874	KH domain-containing, RNA-binding, signal transduction-associated protein 1
18.7	18.7	17.9	18.6	18.7	18.6	1.4300	0.6046	gi|635083921	neutral cholesterol ester hydrolase 1
22.7	23.8	23.0	23.0	23.5	23.6	1.6288	0.6049	gi|635099704	small nuclear ribonucleoprotein Sm D1
18.7	19.6	18.9	19.1	19.4	19.2	1.4516	0.6050	gi|635082031	trifunctional enzyme subunit alpha, mitochondrial
24.2	24.3	23.8	24.1	24.2	24.2	1.2292	0.6054	gi|635038961	40S ribosomal protein S16
22.4	21.8	21.7	22.1	22.4	21.9	1.3732	0.6059	gi|635120567	actin-related protein 2/3 complex subunit 4 isoform X3
25.7	25.8	25.4	25.6	25.9	25.7	1.1845	0.6066	gi|635139449	malate dehydrogenase, mitochondrial isoform X1
20.7	19.9	19.6	20.0	20.7	20.1	1.6508	0.6082	gi|635080395	UTP--glucose-1-phosphate uridylyltransferase isoform X3
20.9	21.3	20.8	20.9	21.0	20.8	0.8052	0.6083	gi|635086028	programmed cell death 6-interacting protein isoform X2
19.4	20.3	19.9	20.5	19.9	19.7	1.5552	0.6085	gi|635067417	leukotriene A-4 hydrolase isoform X3
24.0	24.3	23.9	23.9	24.0	24.1	0.8272	0.6090	gi|635061448	nucleolin
23.2	22.8	22.2	23.0	22.9	22.7	1.4610	0.6091	gi|635056476	thioredoxin-dependent peroxide reductase, mitochondrial
14.1	15.2	14.4	13.9	15.3	15.4	2.0540	0.6098	gi|635106586	glomulin isoform X3
15.3	17.8	18.3	18.4	17.1	17.5	3.5018	0.6111	gi|635092387	dnaJ homolog subfamily C member 7 isoform X3
19.3	20.0	19.0	19.0	19.7	19.0	0.6243	0.6130	gi|635072392	sialic acid synthase
20.2	19.8	19.4	20.1	19.5	20.2	1.5074	0.6132	gi|635100421	BH3-interacting domain death agonist isoform X3
21.0	21.9	21.4	22.0	22.0	21.0	1.6914	0.6139	gi|635109711	adenylate kinase 2, mitochondrial isoform X3
22.7	22.9	22.4	22.7	22.5	23.1	1.3247	0.6146	gi|635086597	60S ribosomal protein L35a isoform X2
25.6	25.8	25.6	25.6	25.8	25.8	1.1220	0.6149	gi|635014753	L-lactate dehydrogenase A chain isoform X1
18.5	17.6	17.2	18.0	17.9	18.0	1.6154	0.6154	gi|635054551	phenazine biosynthesis-like domain-containing protein isoform X3
23.1	23.9	23.7	23.2	23.3	23.6	0.7018	0.6167	gi|635078830	60S ribosomal protein L31
13.0	13.7	10.8	12.4	13.9	12.7	3.3549	0.6170	gi|635081111	thyroid adenoma-associated protein isoform X6
19.0	18.7	17.9	18.4	19.0	18.8	1.6074	0.6192	gi|635072850	stomatin-like protein 2, mitochondrial
17.6	18.7	18.1	18.3	18.1	17.5	0.6123	0.6194	gi|635082029	trifunctional enzyme subunit beta, mitochondrial
20.5	22.4	20.5	21.9	21.8	20.8	2.4503	0.6197	gi|635038851	heterogeneous nuclear ribonucleoprotein L isoform X4
19.7	20.4	18.1	19.4	20.2	19.7	2.4097	0.6202	gi|635030879	ribosomal L1 domain-containing protein 1
23.1	24.1	23.6	23.1	23.9	23.2	0.6307	0.6209	gi|635106063	proteasome subunit alpha type-5 isoform X1
19.5	22.8	22.0	21.5	22.3	22.0	3.3964	0.6222	gi|635022058	destrin
15.6	15.3	12.9	12.2	15.6	13.9	0.2018	0.6223	gi|635027852	nuclear pore complex protein Nup155
21.4	21.8	21.3	21.1	21.4	21.6	0.7858	0.6225	gi|635065615	prefoldin subunit 5
21.4	21.8	21.7	21.1	21.7	21.7	0.7409	0.6227	gi|635119955	26S proteasome non-ATPase regulatory subunit 6 isoform X1
17.3	17.6	15.6	16.1	18.2	17.6	2.9344	0.6228	gi|635043215	annexin A3

19.3	19.8	18.9	19.2	19.1	19.2	0.7128	0.6237	gi|635011135	AP-2 complex subunit alpha-2 isoform X3
19.9	19.7	19.4	19.4	19.8	19.5	0.8145	0.6245	gi|635145500	apoptosis-inducing factor 1, mitochondrial isoform X2
14.4	14.6	13.6	13.9	14.5	14.8	1.6284	0.6245	gi|635141099	protein unc-45 homolog A isoform X4
19.1	19.4	18.8	19.3	19.4	18.9	1.3192	0.6256	gi|635039000	rRNA 2-O-methyltransferase fibrillarin
17.7	19.7	20.8	19.8	19.0	17.7	0.2643	0.6262	gi|635119169	aminoacylase-1 isoform X4
19.5	20.3	19.7	20.1	19.0	19.7	0.6219	0.6268	gi|635090143	integrin alpha-3 isoform X1
20.3	21.4	19.8	20.6	20.8	20.9	1.7649	0.6278	gi|635045558	translation machinery-associated protein 16 isoform X2
20.9	20.5	20.1	20.2	20.6	20.2	0.7238	0.6279	gi|635147704	lysosome-associated membrane glycoprotein 2 isoform X3
19.8	19.6	19.2	19.4	19.8	19.8	1.3180	0.6285	gi|635060903	tubulin alpha-4A chain isoform X3
21.7	20.3	20.4	20.7	21.0	21.4	1.7967	0.6290	gi|635011413	nucleosome assembly protein 1-like 4 isoform X5
21.7	21.8	21.2	21.4	21.6	21.6	0.7860	0.6297	gi|635145809	RNA-binding motif protein, X chromosome isoform X2
26.3	26.2	26.0	26.3	26.1	26.1	0.8666	0.6299	gi|635102142	galectin-1
22.8	23.0	22.6	22.7	23.1	22.7	1.2298	0.6301	gi|635051439	rab GDP dissociation inhibitor beta
18.8	18.4	18.6	18.8	17.5	18.7	0.6020	0.6320	gi|635123720	GTP-binding protein SAR1b isoform X2
21.2	22.2	21.9	21.7	21.9	21.1	0.6215	0.6327	gi|635015024	proteasome subunit alpha type-1 isoform X2
24.3	24.8	24.4	24.1	24.7	24.4	0.7639	0.6333	gi|635131696	phosphoglycerate mutase 1
14.3	14.3	13.3	13.9	14.4	14.2	1.5359	0.6345	gi|635073737	ran-binding protein 6 isoform X2
21.7	23.0	22.9	21.6	24.0	23.3	2.5900	0.6350	gi|635096734	histone H2A type 1-H
14.8	15.6	14.4	14.8	15.3	15.3	1.6211	0.6353	gi|635014300	N-acetyltransferase 10
17.5	17.6	16.9	16.8	17.3	17.4	0.7029	0.6360	gi|635066600	N-acetylglucosamine-6-sulfatase
22.1	22.6	22.6	22.3	22.4	22.2	0.8019	0.6365	gi|635142711	eukaryotic translation initiation factor 1A, X-chromosomal
17.8	19.2	20.0	19.6	18.3	18.0	0.3897	0.6379	gi|635129566	eukaryotic translation initiation factor 5 isoform X2
23.1	23.9	23.2	23.5	23.8	23.4	1.3971	0.6381	gi|635130058	ADP-ribosylation factor 1 isoform X3
22.8	22.9	22.6	22.5	22.8	22.9	0.8313	0.6395	gi|635058664	lupus La protein
19.4	20.2	19.5	20.2	20.0	19.4	1.4982	0.6404	gi|635033049	alanine--tRNA ligase, cytoplasmic
21.0	21.7	21.2	21.3	20.6	21.5	0.6861	0.6408	gi|635103350	eukaryotic translation initiation factor 3 subunit L
20.4	20.1	19.7	20.0	20.3	20.3	1.2918	0.6409	gi|635065975	ras-related protein Rab-5B
19.3	19.9	21.0	19.6	20.1	19.7	0.5536	0.6420	gi|635092175	proteasome activator complex subunit 3 isoform X2
27.1	27.1	27.0	27.1	27.4	27.0	1.1489	0.6431	gi|635065096	tubulin alpha-1A chain isoform X2
20.1	19.9	19.7	19.6	20.3	20.2	1.3220	0.6432	gi|635016929	phosphatidylinositol-binding clathrin assembly protein isoform X13
15.2	15.2	14.1	14.6	15.3	15.2	1.6452	0.6448	gi|635139031	cyclin-G-associated kinase isoform X4
22.3	22.8	22.5	22.4	22.7	22.1	0.7859	0.6450	gi|635027616	activated RNA polymerase II transcriptional coactivator p15
19.7	19.9	19.3	19.6	19.8	19.9	1.2583	0.6456	gi|635082464	ATP-dependent RNA helicase DDX1
16.1	16.0	15.7	15.2	15.8	16.3	0.6850	0.6473	gi|635015184	eukaryotic translation initiation factor 4 gamma 2
18.5	18.4	18.4	18.1	18.5	18.4	0.8500	0.6474	gi|635058942	alkyldihydroxyacetonephosphate synthase, peroxisomal isoform X3
19.0	18.9	18.6	18.7	19.3	18.8	1.3001	0.6483	gi|635081793	protein phosphatase 1G
21.0	22.1	21.3	21.4	21.0	21.4	0.6782	0.6490	gi|635116647	putative RNA-binding protein Luc7-like 2 isoform X5
19.8	19.2	18.6	19.2	19.4	19.6	1.4877	0.6495	gi|635028098	succinate dehydrogenase [ubiquinone] flavoprotein subunit, mitochondrial
21.7	22.0	22.3	22.4	22.5	21.6	1.4293	0.6499	gi|635033554	nuclear transport factor 2 isoform X2
20.6	22.2	21.2	22.3	21.9	20.8	2.0232	0.6504	gi|635065947	CD63 antigen
22.9	23.0	22.8	22.4	22.7	23.2	0.7793	0.6510	gi|635100745	ran-specific GTPase-activating protein isoform X2

24.8	24.5	24.3	24.4	24.6	24.3	0.8461	0.6518	gi|635059577	60 kDa heat shock protein, mitochondrial
18.7	18.9	18.3	18.6	18.5	19.2	1.3504	0.6529	gi|635044862	N-alpha-acetyltransferase 15, NatA auxiliary subunit isoform X3
19.2	19.6	19.1	19.4	19.4	19.2	1.2152	0.6530	gi|635028725	microtubule-associated protein 1B isoform X3
20.1	20.7	20.0	20.2	20.6	20.4	1.3332	0.6532	gi|635088315	serine hydroxymethyltransferase, cytosolic isoform X1
25.2	25.3	25.1	25.0	25.4	25.2	1.1514	0.6549	gi|635117835	60S ribosomal protein L14 isoform X2
19.7	21.3	20.4	20.3	20.3	20.0	0.5924	0.6555	gi|635027630	threonine--tRNA ligase, cytoplasmic isoform X1
18.7	20.8	20.2	19.9	20.5	20.2	1.9988	0.6556	gi|635147631	ADP/ATP translocase 2
20.2	20.8	20.6	20.4	20.8	20.7	1.2447	0.6559	gi|635091037	cAMP-dependent protein kinase type I-alpha regulatory subunit
26.2	26.4	26.0	26.1	26.7	26.2	1.2927	0.6568	gi|635018648	heat shock cognate 71 kDa protein isoform X3
17.5	20.8	18.4	18.0	18.5	18.8	0.3257	0.6571	gi|635092393	2,3-cyclic-nucleotide 3-phosphodiesterase isoform X2
22.6	23.2	22.7	22.8	22.7	22.7	0.8209	0.6587	gi|635052592	integrin beta-1
17.8	18.4	17.5	17.3	18.1	17.8	0.6829	0.6597	gi|635036576	ATP-dependent RNA helicase DDX39A
21.0	20.8	20.1	20.4	21.0	20.9	1.4133	0.6598	gi|635069578	actin-related protein 2/3 complex subunit 3
15.4	16.2	15.3	15.5	16.2	15.8	1.4755	0.6599	gi|635087024	myb-binding protein 1A isoform X1
22.8	23.8	23.4	23.5	23.0	22.9	0.6762	0.6601	gi|635071246	60S ribosomal protein L35
22.5	22.9	21.3	22.4	22.8	22.2	1.7395	0.6602	gi|635133612	heterogeneous nuclear ribonucleoprotein U isoform X2
20.6	19.9	19.6	20.0	20.3	20.2	1.4195	0.6606	gi|635044770	membrane-associated progesterone receptor component 2
18.4	18.2	17.9	17.8	18.3	18.1	0.7938	0.6608	gi|635043595	estradiol 17-beta-dehydrogenase 11 isoform X2
24.8	25.1	24.8	24.7	24.9	24.9	0.8760	0.6612	gi|635035336	elongation factor 2
17.7	17.8	17.4	17.8	18.0	17.4	1.2644	0.6615	gi|635132241	torsin-1A-interacting protein 1 isoform X4
20.2	20.5	19.8	20.7	20.0	20.1	1.3767	0.6618	gi|635123660	serine/threonine-protein phosphatase 2A catalytic subunit alpha isoform
15.7	16.4	15.8	15.6	16.7	16.0	1.5248	0.6619	gi|635124954	la-related protein 1 isoform X4
20.3	20.9	21.4	20.7	21.6	20.9	1.5889	0.6621	gi|635020823	serine/arginine-rich splicing factor 6
17.9	18.2	17.8	17.5	17.9	18.1	0.8019	0.6628	gi|635062945	condensin complex subunit 1 isoform X2
22.8	23.4	23.3	22.6	23.1	23.4	0.7053	0.6629	gi|635120791	60S ribosomal protein L32
19.5	21.3	21.0	20.6	21.0	19.2	0.4386	0.6631	gi|635110729	UDP-glucose 4-epimerase isoform X2
15.5	16.0	15.2	15.1	16.5	15.7	1.6406	0.6633	gi|635124202	histidine--tRNA ligase, cytoplasmic
19.7	19.5	19.1	19.3	19.6	19.8	1.2758	0.6644	gi|635055592	aspartate aminotransferase, cytoplasmic
18.5	19.2	18.0	18.3	19.1	17.6	0.5609	0.6656	gi|635027677	ribosome biogenesis protein BRX1 homolog
15.4	15.6	14.6	14.8	15.8	15.5	1.5864	0.6674	gi|635028426	importin-11
18.7	18.3	18.7	19.0	18.2	19.0	1.3666	0.6693	gi|635126053	phosphoenolpyruvate carboxykinase [GTP], mitochondrial isoform X4
21.8	22.2	22.0	22.2	22.2	21.9	1.1754	0.6698	gi|635105509	sodium/potassium-transporting ATPase subunit alpha-1 isoform X2
23.6	24.2	23.5	23.3	23.9	23.7	0.7426	0.6699	gi|635098964	ATP synthase subunit alpha, mitochondrial
23.3	23.2	22.7	22.8	23.1	22.9	0.8035	0.6706	gi|635105760	F-actin-capping protein subunit alpha-1
22.4	22.2	21.4	21.7	22.1	21.8	0.7055	0.6710	gi|635129312	tryptophan--tRNA ligase, cytoplasmic isoform X2
17.0	16.7	15.9	16.3	16.9	16.9	1.4574	0.6724	gi|635030710	4-aminobutyrate aminotransferase, mitochondrial isoform X5
24.2	25.4	24.2	24.2	24.5	24.5	0.6506	0.6755	gi|635096112	chloride intracellular channel protein 1
24.0	24.7	24.1	24.4	24.4	24.2	1.2504	0.6765	gi|635125937	guanine nucleotide-binding protein subunit beta-2-like 1
17.5	19.0	14.7	18.4	16.2	18.7	4.6756	0.6777	gi|635054706	cyclin-dependent kinase 1
19.9	20.0	19.4	19.0	19.7	20.1	0.6849	0.6778	gi|635086850	platelet-activating factor acetylhydrolase IB subunit alpha
23.6	23.4	23.7	23.4	23.8	23.6	1.1443	0.6785	gi|635104202	T-complex protein 1 subunit gamma

23.6	23.4	23.3	23.2	23.4	23.5	0.8678	0.6786	gi|635140723	40S ribosomal protein S17 isoform X2
18.2	18.2	17.7	18.0	18.5	17.9	1.3119	0.6787	gi|635147552	extended synaptotagmin-2-like
21.9	22.8	22.4	22.3	22.8	22.5	1.3773	0.6793	gi|635025422	translationally-controlled tumor protein
20.7	20.8	19.9	19.0	20.8	20.8	0.5057	0.6806	gi|635126443	cofilin-2 isoform X3
23.8	23.9	23.4	23.6	23.8	23.9	1.1971	0.6809	gi|635066506	40S ribosomal protein S13
20.0	19.9	18.1	19.6	20.2	19.1	2.0126	0.6811	gi|635023773	splicing factor U2AF 35 kDa subunit isoform X1
22.8	23.4	23.1	23.0	23.3	23.3	1.2134	0.6815	gi|635145639	hypoxanthine-guanine phosphoribosyltransferase
20.0	20.6	20.0	20.3	20.5	20.1	1.2854	0.6832	gi|635091770	glycylpeptide N-tetradecanoyltransferase 1
23.9	24.3	23.6	23.9	24.1	24.1	1.2349	0.6837	gi|635072864	transitional endoplasmic reticulum ATPase isoform X1
21.6	20.3	20.7	20.7	21.0	21.4	1.5430	0.6842	gi|635044198	oligosaccharyltransferase complex subunit OSTC isoform X2
19.4	20.0	19.8	19.8	20.2	18.6	0.5976	0.6858	gi|635069530	vacuolar protein sorting-associated protein 29-like
20.3	21.0	20.3	20.6	20.6	20.7	1.2539	0.6862	gi|635140070	aminoacyl tRNA synthase complex-interacting multifunctional protein 2 isoform X1
14.9	16.0	13.7	13.5	15.8	14.2	0.3912	0.6867	gi|635081618	EH domain-containing protein 3 isoform X2
15.6	16.2	15.1	15.4	16.1	15.9	1.4765	0.6872	gi|635011689	serine/threonine-protein phosphatase 6 regulatory subunit 3 isoform X13
21.1	21.6	21.5	21.1	21.5	21.4	0.8121	0.6874	gi|635066285	serine hydroxymethyltransferase, mitochondrial isoform X6
18.9	18.4	17.5	18.1	19.0	18.4	1.6057	0.6893	gi|635088561	COP9 signalosome complex subunit 3
13.6	12.8	13.2	12.7	13.4	14.0	1.5160	0.6897	gi|635040622	DNA polymerase delta catalytic subunit isoform X1
22.4	22.6	22.3	22.2	22.6	22.3	0.8660	0.6901	gi|635144038	moesin isoform X2
24.3	24.7	24.5	24.3	24.4	24.6	0.8572	0.6902	gi|635080170	annexin A4
19.5	20.4	19.5	19.7	20.5	19.7	1.5151	0.6912	gi|635034983	lamin-B2 isoform X2
25.3	25.6	25.3	25.3	25.6	25.6	1.1492	0.6913	gi|635144463	phosphoglycerate kinase 1
19.5	20.0	19.9	20.0	19.7	19.5	0.8108	0.6916	gi|635071992	prostaglandin reductase 1 isoform X2
18.4	19.4	18.6	18.9	19.1	18.7	1.3693	0.6917	gi|635032545	nuclear pore complex protein Nup93 isoform X2
18.9	19.7	19.7	19.9	19.3	19.6	1.3592	0.6919	gi|635031803	dCTP pyrophosphatase 1
19.4	19.9	19.3	19.3	19.3	19.7	0.7847	0.6926	gi|635064916	6-phosphofructokinase, muscle type isoform X4
16.3	16.4	15.3	15.9	16.5	16.1	1.4653	0.6931	gi|635020702	serine/threonine-protein kinase 4 isoform X2
20.1	16.1	18.3	19.3	17.0	16.4	0.2472	0.6950	gi|635035126	guanine nucleotide-binding protein subunit alpha-11
21.5	21.5	21.1	21.2	21.3	21.4	0.8666	0.6950	gi|635114607	replication protein A 14 kDa subunit
21.6	21.3	21.2	21.3	21.1	21.5	0.8491	0.6951	gi|635125273	arginine--tRNA ligase, cytoplasmic
18.7	19.3	18.6	18.7	19.1	18.5	0.7758	0.6952	gi|635012005	dipeptidyl peptidase 3
19.0	20.7	18.3	18.8	19.4	18.9	0.4877	0.6967	gi|635083513	DNA-directed RNA polymerases I, II, and III subunit RPABC3
18.7	19.6	19.4	19.5	19.5	18.0	0.5827	0.6974	gi|635018350	probable ATP-dependent RNA helicase DDX6
17.7	16.7	16.1	16.6	17.7	17.0	1.7436	0.6975	gi|635071536	erythrocyte band 7 integral membrane protein
16.2	17.9	17.4	16.6	17.9	17.8	1.8780	0.6976	gi|635141412	U2 small nuclear ribonucleoprotein A
18.5	19.1	18.3	18.3	18.7	18.6	0.7812	0.6978	gi|635100620	crk-like protein
20.1	21.3	21.3	21.1	21.2	20.9	1.4922	0.6979	gi|635112983	phosphoserine phosphatase
19.7	20.0	19.3	19.4	19.8	19.6	0.7875	0.6980	gi|635013048	DNA damage-binding protein 1
19.5	19.7	20.1	20.7	19.7	19.4	1.4949	0.6983	gi|635090305	serine/arginine-rich splicing factor 2
15.8	17.5	16.0	16.3	15.9	16.4	0.5847	0.6987	gi|635139710	thyroid receptor-interacting protein 6 isoform X2
24.2	24.3	23.9	23.7	24.5	23.9	0.7748	0.6995	gi|635066208	nascent polypeptide-associated complex subunit alpha
18.8	20.1	18.4	18.9	19.5	19.6	1.7170	0.7008	gi|635126999	glucosamine 6-phosphate N-acetyltransferase

23.4	23.7	23.2	23.5	23.4	23.6	1.1507	0.7009	gi|635116144	filamin-C isoform X2
21.3	22.1	21.5	21.6	22.2	21.5	1.3640	0.7018	gi|635141834	proteasome subunit beta type-5 isoform X1
16.9	15.3	14.0	16.9	14.3	16.4	3.0421	0.7025	gi|635079850	dynactin subunit 1 isoform X11
18.5	19.7	19.7	19.6	18.7	19.0	0.6401	0.7035	gi|635030720	phosphomannomutase 2 isoform X1
18.9	18.8	18.4	18.6	19.2	18.8	1.2528	0.7035	gi|635125648	PDZ and LIM domain protein 7 isoform X4
20.5	20.4	19.6	19.7	20.7	20.7	1.5298	0.7043	gi|635108339	UMP-CMP kinase
15.8	15.9	14.8	15.3	15.8	16.0	1.4685	0.7050	gi|635055455	MMS19 nucleotide excision repair protein homolog isoform X3
20.0	21.7	20.6	19.8	21.4	20.1	0.5154	0.7054	gi|635029119	40S ribosomal protein S23
20.4	20.3	19.7	19.7	20.3	20.1	0.7730	0.7056	gi|635098407	mucosa-associated lymphoid tissue lymphoma translocation protein 1 isoform X2
17.9	17.6	17.0	17.0	17.6	18.4	1.5601	0.7057	gi|635093624	L-xylulose reductase
19.3	19.9	18.7	19.0	20.0	19.4	1.5329	0.7057	gi|635059570	splicing factor 3B subunit 1 isoform X2
19.5	20.1	19.6	19.9	20.1	19.5	1.3000	0.7059	gi|635015034	ras-related protein R-Ras2
20.7	21.4	20.3	20.0	20.7	21.1	0.6477	0.7068	gi|635037175	coatomer subunit epsilon
24.2	24.8	24.6	24.5	25.1	24.4	1.3061	0.7074	gi|635051073	60S ribosomal protein L8
16.6	16.2	16.1	15.9	16.2	16.5	0.7835	0.7075	gi|635100210	proteasome assembly chaperone 2 isoform X2
15.4	16.1	14.7	15.0	15.6	15.1	0.6695	0.7098	gi|635042400	ubiquitin-like modifier-activating enzyme 6 isoform X2
22.3	22.0	21.8	21.6	22.4	21.8	0.7723	0.7110	gi|635021820	dynein light chain roadblock-type 1
17.6	17.2	16.6	16.5	17.5	16.9	0.6981	0.7112	gi|635147913	deoxyribonuclease-1-like 1
19.1	19.1	17.9	19.2	18.7	18.5	1.4952	0.7137	gi|635113429	drebrin-like protein isoform X3
20.1	20.6	20.6	20.4	20.7	20.5	1.1936	0.7141	gi|635142821	eukaryotic translation initiation factor 2 subunit 3
25.6	25.8	25.7	25.6	25.7	25.9	1.1230	0.7143	gi|635089952	nucleoside diphosphate kinase A
23.7	24.4	23.7	23.6	24.1	24.5	1.3711	0.7143	gi|635110740	60S ribosomal protein L11
20.2	20.8	20.2	20.0	20.5	20.4	0.7963	0.7154	gi|635087747	phosphoribosylformylglycinamidine synthase
16.3	18.3	17.2	16.7	18.5	17.6	2.0081	0.7160	gi|635145159	ribose-phosphate pyrophosphokinase 1
20.4	21.0	20.6	20.7	20.7	20.5	0.8696	0.7181	gi|635056871	glutaredoxin-3
21.1	20.7	20.1	20.1	20.6	20.8	0.7262	0.7187	gi|635036387	peroxiredoxin-2
21.3	21.1	20.6	20.8	21.2	21.2	1.2319	0.7192	gi|635086640	coatomer subunit beta
24.0	24.3	23.9	23.9	24.1	24.3	1.1470	0.7199	gi|635062909	CD9 antigen
19.2	19.7	18.6	18.8	19.1	19.1	0.7388	0.7205	gi|635123774	core histone macro-H2A.1 isoform X4
20.0	20.7	20.3	20.6	20.5	20.2	1.2190	0.7211	gi|635037700	cytochrome b-c1 complex subunit Rieske, mitochondrial
23.0	23.9	22.5	22.9	23.8	23.3	1.5612	0.7211	gi|635116486	myotrophin
22.1	22.6	21.6	22.0	22.2	21.8	0.7540	0.7213	gi|635066098	citrate synthase, mitochondrial isoform X2
17.6	18.3	16.7	17.0	18.1	18.2	1.7046	0.7216	gi|635093572	ethanolamine-phosphate cytidylyltransferase isoform X3
21.9	22.5	22.1	22.2	22.6	22.0	1.2402	0.7223	gi|635143237	ubiquitin-like modifier-activating enzyme 1 isoform X3
18.6	17.4	16.5	17.2	17.5	18.7	1.9810	0.7224	gi|635055114	insulin-degrading enzyme isoform X3
15.8	16.7	15.2	13.9	16.5	16.2	0.4365	0.7229	gi|635057139	UDP-glucose:glycoprotein glucosyltransferase 1
20.7	21.3	20.7	20.6	20.7	21.1	0.8057	0.7230	gi|635115796	F-actin-capping protein subunit alpha-2
21.2	22.2	20.9	21.8	21.8	21.2	1.4912	0.7231	gi|635102550	NHP2-like protein 1 isoform X2
20.2	20.0	19.7	19.8	20.4	20.1	1.2073	0.7233	gi|635084904	sodium/potassium-transporting ATPase subunit beta-3 isoform X3
23.8	24.0	23.4	23.5	24.2	23.9	1.3043	0.7251	gi|635137880	60S ribosomal protein L9
19.1	19.3	18.5	18.3	19.1	19.0	0.7478	0.7254	gi|635098242	apoptosis regulator Bcl-2

19.5	20.7	20.2	20.5	20.5	19.9	1.4401	0.7254	gi|635130443	3(2),5-bisphosphate nucleotidase 1
21.5	21.8	21.1	21.3	21.8	20.9	0.7480	0.7257	gi|635024825	heat shock protein 105 kDa isoform X2
17.1	17.5	17.4	16.7	17.6	17.4	0.7748	0.7276	gi|635126107	magnesium-dependent phosphatase 1 isoform X5
24.2	23.8	23.8	24.2	24.0	23.3	0.7712	0.7278	gi|635095836	40S ribosomal protein S18
21.4	21.1	21.0	21.0	21.5	21.3	1.1666	0.7278	gi|635037870	programmed cell death protein 5 isoform X2
23.0	23.3	22.9	23.2	23.1	23.1	1.1231	0.7295	gi|635109198	adenylyl cyclase-associated protein 1 isoform X1
17.9	19.3	19.2	19.0	18.7	19.2	1.4991	0.7298	gi|635118578	cytochrome b-c1 complex subunit 1, mitochondrial
21.3	20.9	20.2	20.7	21.1	21.2	1.3698	0.7298	gi|635106483	glutamate--cysteine ligase regulatory subunit
25.3	25.5	24.8	25.1	25.4	25.4	1.2090	0.7307	gi|635044581	annexin A5
20.5	20.3	19.7	19.8	20.3	20.1	0.7810	0.7320	gi|635080465	exportin-1 isoform X1
23.7	25.0	24.2	24.4	24.6	24.3	1.3701	0.7335	gi|635142241	brain acid soluble protein 1
19.4	20.3	19.6	19.5	20.1	19.9	1.2996	0.7341	gi|635143387	putative RNA-binding protein 3
21.4	21.5	21.3	21.3	21.7	21.4	1.1241	0.7346	gi|635057694	aspartate--tRNA ligase, cytoplasmic
23.8	24.2	23.6	23.7	23.9	23.8	0.8648	0.7354	gi|635146477	glucose-6-phosphate 1-dehydrogenase isoform X7
23.0	23.4	23.0	22.9	23.2	23.1	0.8744	0.7355	gi|635039073	flavin reductase (NADPH) isoform X1
17.3	17.5	16.6	16.5	17.3	17.1	0.7496	0.7364	gi|635037169	regulator of nonsense transcripts 1 isoform X4
20.2	21.1	19.4	20.4	20.9	20.0	1.5753	0.7372	gi|635035551	far upstream element-binding protein 2 isoform X4
19.1	20.9	20.1	18.3	20.6	20.3	0.4856	0.7375	gi|635050269	protein NDRG1 isoform X5
19.6	20.4	20.1	20.0	20.7	19.9	1.3197	0.7377	gi|635045006	ATP-binding cassette sub-family E member 1
19.3	19.8	19.2	19.2	19.6	19.8	1.2491	0.7377	gi|635113345	2-oxoglutarate dehydrogenase, mitochondrial isoform X3
21.4	21.9	21.3	21.2	21.5	21.7	0.8367	0.7381	gi|635125810	sequestosome-1 isoform X2
18.6	18.9	17.6	18.5	18.8	18.4	1.4089	0.7388	gi|635126282	sec1 family domain-containing protein 1 isoform X1
18.3	19.0	17.8	17.3	18.5	18.6	0.6456	0.7402	gi|635039808	vasodilator-stimulated phosphoprotein isoform X3
20.1	20.2	20.2	19.9	20.3	20.4	1.1177	0.7412	gi|635146559	prefoldin subunit 3
21.0	21.5	21.1	21.4	21.2	21.3	1.1560	0.7420	gi|635124121	prefoldin subunit 1 isoform X4
19.3	18.2	18.6	18.6	18.5	18.7	0.7758	0.7422	gi|635088981	bleomycin hydrolase isoform X2
16.1	15.9	15.1	15.6	15.8	15.5	0.7820	0.7422	gi|635063661	probable ATP-dependent RNA helicase DDX47
20.9	20.9	20.4	20.5	20.8	20.8	0.8527	0.7436	gi|635044138	aminoacyl tRNA synthase complex-interacting multifunctional protein 1 isoform X2
19.8	19.6	18.2	19.7	19.8	18.7	1.6235	0.7437	gi|635120681	protein SEC13 homolog isoform X4
21.5	22.3	21.9	22.0	22.1	21.8	1.2249	0.7439	gi|635089744	serine/arginine-rich splicing factor 1
20.4	20.7	20.1	20.8	20.3	19.7	0.7441	0.7442	gi|635126917	thioredoxin-related transmembrane protein 1
21.5	22.2	21.6	21.6	22.0	21.8	1.2152	0.7447	gi|635111000	dolichyl-diphosphooligosaccharide--protein glycosyltransferase 48 kDa subunit isoform X2
23.8	24.2	23.7	23.6	24.1	24.2	1.1823	0.7454	gi|635132557	peroxiredoxin-6
14.4	15.1	14.7	14.2	15.4	15.0	1.3745	0.7461	gi|635032485	2-oxoglutarate and iron-dependent oxygenase domain-containing protein 1 isoform X2
22.7	23.3	23.0	23.4	23.3	22.7	1.2466	0.7466	gi|635123430	histidine triad nucleotide-binding protein 1
14.2	15.6	12.4	13.0	15.1	15.4	2.6072	0.7466	gi|635118439	putative ATP-dependent RNA helicase DHX30 isoform X6
19.4	20.0	20.1	19.7	20.0	19.5	0.8102	0.7468	gi|635137496	eukaryotic translation initiation factor 3 subunit J
18.9	18.2	17.8	18.1	18.7	18.5	1.3123	0.7471	gi|635027119	pachytene checkpoint protein 2 homolog
22.1	22.6	22.2	21.9	22.5	22.2	0.8226	0.7474	gi|635118669	inosine-5-monophosphate dehydrogenase 2 isoform X2
22.8	22.8	22.7	22.6	22.8	22.9	0.9294	0.7479	gi|635068305	endoplasmic reticulum resident protein 29 isoform X1
17.4	18.5	16.9	17.6	18.2	17.6	1.5079	0.7492	gi|635070706	serine/threonine-protein phosphatase 2A activator isoform X4

22.8	24.0	23.2	23.2	23.2	23.2	0.7575	0.7492	gi|635104668	tropomyosin alpha-3 chain isoform X8
19.3	19.7	19.4	19.2	19.8	19.8	1.2026	0.7500	gi|635066109	protein canopy homolog 2
22.2	21.9	21.9	21.8	22.1	22.3	1.1394	0.7513	gi|635109089	CTP synthase 1 isoform X2
19.3	19.9	19.7	19.0	19.1	20.4	0.6832	0.7519	gi|635051381	tubulin alpha chain-like 3
18.7	18.5	17.6	18.2	18.4	17.9	0.7521	0.7519	gi|635120895	28S ribosomal protein S25, mitochondrial
16.2	17.5	16.8	17.3	17.3	16.4	1.4238	0.7522	gi|635084821	procollagen-lysine,2-oxoglutarate 5-dioxygenase 2 isoform X4
23.1	23.4	22.8	23.1	23.1	22.9	0.8648	0.7522	gi|635096072	heat shock 70 kDa protein 1A/1B
18.3	18.7	18.4	18.3	18.6	18.7	1.1345	0.7527	gi|635017660	radixin isoform X1
18.5	19.2	17.9	17.9	19.2	19.1	1.5298	0.7536	gi|635028565	splicing regulatory glutamine/lysine-rich protein 1 isoform X8
23.8	24.9	24.2	24.1	24.6	23.8	0.7408	0.7543	gi|635054210	40S ribosomal protein S15a
16.0	16.3	15.9	15.4	16.5	16.0	0.7613	0.7544	gi|635084840	U2 snRNP-associated SURP motif-containing protein isoform X1
17.0	17.1	16.7	17.4	17.5	16.4	1.3528	0.7552	gi|635080728	spectrin beta chain, non-erythrocytic 1 isoform X3
23.5	23.9	23.8	23.5	23.9	23.7	0.8848	0.7565	gi|635112884	6-phosphogluconate dehydrogenase, decarboxylating
17.5	17.2	17.9	16.2	17.4	18.3	0.6104	0.7566	gi|635055963	cytosolic purine 5-nucleotidase isoform X4
15.3	15.9	14.1	14.6	15.8	15.6	1.6521	0.7574	gi|635036365	transportin-2 isoform X4
25.1	25.1	24.8	24.7	25.1	25.5	1.2172	0.7581	gi|635108638	40S ribosomal protein S8 isoform X1
18.0	18.2	17.1	17.4	18.2	18.1	1.3731	0.7586	gi|635093778	tubulin-specific chaperone D isoform X2
19.9	20.0	19.3	19.5	19.9	19.6	0.8309	0.7611	gi|635052538	kinesin-1 heavy chain
19.4	20.9	19.4	20.0	20.5	19.8	1.5134	0.7617	gi|635107125	far upstream element-binding protein 1 isoform X9
18.1	20.2	19.3	19.6	20.1	18.7	1.7149	0.7621	gi|635014049	apoptosis inhibitor 5
23.2	23.6	23.1	23.1	23.4	23.3	0.8819	0.7623	gi|635092337	ras-related protein Rab-5C isoform X2
23.5	24.6	24.5	24.2	24.3	24.4	1.3066	0.7627	gi|635095598	60S ribosomal protein L10a
17.8	18.4	18.3	17.9	18.1	18.2	0.8591	0.7632	gi|635142725	ribosomal protein S6 kinase alpha-3 isoform X6
17.5	19.6	19.5	19.1	19.5	18.7	1.7099	0.7636	gi|635145627	glypican-4
15.4	16.2	14.9	15.0	15.7	16.3	1.4930	0.7640	gi|635019735	opioid growth factor receptor isoform X2
21.8	23.0	22.4	22.5	22.5	21.7	0.7161	0.7648	gi|635083081	low molecular weight phosphotyrosine protein phosphatase isoform X6
20.1	19.6	19.3	19.3	19.9	20.1	1.2671	0.7655	gi|635023948	pituitary tumor-transforming gene 1 protein-interacting protein isoform X5
19.7	20.2	19.4	19.7	20.0	19.8	1.1806	0.7664	gi|635127102	kinectin isoform X8
22.7	23.6	22.9	23.1	23.3	23.0	1.2369	0.7665	gi|635051361	aldo-keto reductase family 1 member C3-like
17.0	15.8	15.8	16.0	16.6	16.4	1.3581	0.7679	gi|635092308	signal transducer and activator of transcription 3 isoform X10
22.1	23.2	22.5	22.6	22.1	22.6	0.7728	0.7680	gi|635110337	nuclear migration protein nudC isoform X2
21.3	22.1	21.4	21.7	21.7	21.6	1.2141	0.7708	gi|635083372	dnaJ homolog subfamily B member 11
19.4	19.4	19.5	19.1	19.8	19.6	1.1701	0.7709	gi|635121177	DNA replication licensing factor MCM2 isoform X2
24.5	25.1	24.5	24.5	25.0	24.8	1.1882	0.7717	gi|635066200	ATP synthase subunit beta, mitochondrial
20.8	21.3	20.0	20.5	21.1	20.1	0.7234	0.7731	gi|635035797	ELAV-like protein 1
20.5	20.5	20.0	20.0	20.6	20.8	1.2154	0.7734	gi|635140562	uncharacterized protein LOC103246949
22.0	22.6	22.1	22.0	22.6	21.9	0.8219	0.7743	gi|635137858	ubiquitin-conjugating enzyme E2 K isoform X2
19.8	19.6	20.1	19.9	19.7	20.1	1.1305	0.7757	gi|635143125	farnesyl pyrophosphate synthase-like
18.4	18.2	18.0	17.7	18.6	18.7	1.2643	0.7777	gi|635142763	spermine synthase isoform X2
21.5	22.2	21.6	21.8	22.0	21.7	1.1553	0.7782	gi|635053952	vinculin isoform X2
20.7	20.2	20.5	20.6	20.3	20.4	0.8924	0.7784	gi|635071982	guanine nucleotide-binding protein G(I)/G(S)/G(O) subunit gamma-10

20.2	20.6	20.1	20.3	20.4	20.3	1.1157	0.7794	gi|635091100	26S proteasome non-ATPase regulatory subunit 12 isoform X1
21.4	21.6	20.6	20.7	21.5	21.0	0.7743	0.7798	gi|635130328	calpain-2 catalytic subunit isoform X3
18.9	18.9	18.7	18.6	18.9	19.1	1.1132	0.7821	gi|635140454	Golgi to ER traffic protein 4 homolog isoform X2
22.0	22.4	21.6	21.9	22.2	21.7	0.8295	0.7821	gi|635124529	dihydropyrimidinase-related protein 3 isoform X1
15.9	16.1	14.8	15.5	16.2	15.4	1.3844	0.7822	gi|635048008	DNA-dependent protein kinase catalytic subunit isoform X2
24.0	24.2	23.5	23.6	24.1	24.0	1.1865	0.7833	gi|635104925	protein S100-A10 isoform X2
19.3	19.3	19.0	19.0	19.5	19.4	1.1432	0.7837	gi|635021516	minor histocompatibility antigen H13 isoform X2
21.2	22.1	21.7	22.0	21.4	21.3	0.8011	0.7840	gi|635100204	tubulin beta-6 chain
16.2	17.3	16.3	16.1	17.2	16.8	1.3854	0.7847	gi|635072952	dynactin subunit 3 isoform X3
19.0	19.2	18.9	18.9	19.1	19.2	1.0795	0.7862	gi|635081861	CAD protein
22.2	21.5	21.8	22.9	21.1	21.9	1.4599	0.7871	gi|635104692	40S ribosomal protein S27
25.9	25.9	25.5	25.5	26.2	25.9	1.1851	0.7874	gi|635049432	14-3-3 protein zeta/delta
18.0	17.9	17.1	17.6	17.9	17.9	1.2194	0.7874	gi|635095005	serine/threonine-protein phosphatase 2A 56 kDa regulatory subunit delta isoform
17.7	18.3	17.2	17.5	18.3	17.9	1.3043	0.7877	gi|635062184	protein phosphatase 1 regulatory subunit 7 isoform X5
22.6	23.6	22.6	22.6	23.1	22.6	0.7840	0.7878	gi|635132429	calcyclin-binding protein isoform X2
17.3	16.2	16.4	16.6	16.5	17.1	1.2856	0.7880	gi|635057745	kynureninase isoform X2
22.2	22.3	22.0	21.8	22.8	22.2	1.2203	0.7887	gi|635134098	proteasome subunit alpha type-4 isoform X2
21.9	22.1	21.5	21.5	22.3	21.9	1.1878	0.7890	gi|635082041	ras-related protein Rab-10
18.2	18.8	17.8	17.8	18.0	18.7	0.7659	0.7892	gi|635012758	atlastin-3 isoform X2
16.2	16.2	15.6	15.6	16.4	16.2	1.2217	0.7894	gi|635093391	E3 ubiquitin-protein ligase RNF213 isoform X2
19.2	19.9	18.4	19.0	19.0	19.1	0.7492	0.7904	gi|635083533	eukaryotic translation initiation factor 4 gamma 1 isoform X5
25.3	26.0	25.0	25.6	25.4	25.5	1.2048	0.7904	gi|635140804	60S ribosomal protein L7a
21.6	23.1	22.2	22.6	22.2	22.4	1.3411	0.7914	gi|635080331	actin-related protein 2
24.7	25.1	24.7	24.3	24.7	25.9	1.3825	0.7919	gi|635057886	L-lactate dehydrogenase A chain
15.9	17.6	16.6	15.6	17.7	16.1	0.5844	0.7925	gi|635123556	kinesin-like protein KIF3A isoform X10
19.5	18.9	18.0	18.7	19.3	18.7	1.3828	0.7927	gi|635067985	coronin-1C isoform X3
17.6	18.1	17.0	17.5	17.7	17.2	0.8033	0.7936	gi|635026894	cullin-4A isoform X2
18.6	19.9	19.7	19.2	19.3	20.1	1.3607	0.7936	gi|635128132	dihydrolipoyllysine-residue succinyltransferase component of 2-oxoglutarate dehydrogenase complex, mitochondrial
19.2	19.5	19.1	19.2	19.7	18.7	0.8246	0.7940	gi|635061054	phenylalanine--tRNA ligase beta subunit
18.7	19.4	18.2	19.6	18.9	18.2	1.3897	0.7940	gi|635088412	fatty aldehyde dehydrogenase isoform X4
21.9	21.9	20.8	21.5	21.8	21.6	1.2655	0.7942	gi|635060458	bifunctional purine biosynthesis protein PURH isoform X1
20.9	21.4	21.1	20.9	21.2	21.2	0.8932	0.7949	gi|635079545	S-adenosylmethionine synthase isoform type-2
22.1	21.8	21.6	21.8	21.9	21.6	0.8982	0.7958	gi|635049324	ribonuclease UK114
27.8	28.0	27.6	27.8	28.0	27.7	1.1133	0.7959	gi|635093488	actin, cytoplasmic 2
21.8	21.9	21.3	21.4	21.7	21.7	0.8718	0.7963	gi|635083535	26S proteasome non-ATPase regulatory subunit 2
19.7	20.0	18.7	19.2	19.6	20.0	1.3251	0.7964	gi|635042196	transcription intermediary factor 1-beta isoform X2
20.1	20.6	20.3	20.2	20.9	20.1	1.1964	0.7967	gi|635047011	zinc transporter ZIP14 isoform X2
21.3	22.5	22.2	21.5	22.0	22.2	0.7682	0.7972	gi|635034824	cold-inducible RNA-binding protein isoform X2
17.5	18.4	16.5	17.3	17.7	17.0	0.6855	0.7972	gi|635147230	splicing factor 3B subunit 4
19.1	19.6	18.5	18.7	19.7	19.2	1.3188	0.7989	gi|635043347	enolase-phosphatase E1 isoform X2
17.3	17.4	17.0	16.7	17.9	17.4	1.2482	0.7997	gi|635050639	gasdermin-D isoform X3

24.7	24.6	23.3	24.5	24.3	24.1	1.3433	0.8008	gi|635074755	prothymosin alpha
22.1	22.5	22.2	22.1	22.7	22.3	1.1461	0.8025	gi|635049421	polyadenylate-binding protein 1
17.4	17.8	17.2	17.1	17.7	17.5	0.8579	0.8032	gi|635086793	unconventional myosin-Ic
18.1	18.0	17.7	17.9	18.2	17.9	1.0954	0.8055	gi|635103668	nicastrin isoform X2
17.2	19.0	18.8	17.3	19.6	18.7	1.7004	0.8059	gi|635122674	1,4-alpha-glucan-branching enzyme
22.1	22.2	21.7	21.9	22.1	22.0	1.1115	0.8059	gi|635012585	peroxiredoxin-5, mitochondrial isoform X1
19.5	19.8	19.5	19.5	19.8	19.7	1.0954	0.8061	gi|635146912	glycogen phosphorylase, brain form isoform X4
18.7	18.7	16.3	17.7	18.9	17.9	1.6917	0.8078	gi|635025875	paraspeckle component 1
17.9	18.3	17.4	17.3	18.0	18.1	0.8072	0.8080	gi|635059413	glutaminase kidney isoform, mitochondrial isoform X2
19.2	17.7	17.9	18.0	18.3	18.2	0.7503	0.8083	gi|635066592	exportin-T isoform X2
18.9	18.9	18.6	19.3	18.1	18.7	0.8157	0.8085	gi|635046021	caspase-3
22.8	23.7	22.7	22.7	23.1	23.2	0.8058	0.8096	gi|635081686	serine/threonine-protein phosphatase PP1-beta catalytic subunit
25.2	25.5	25.3	25.4	25.4	25.2	1.0674	0.8098	gi|635093546	protein disulfide-isomerase isoform X1
19.7	20.3	19.5	19.9	20.0	19.9	1.1624	0.8098	gi|635077828	dynein light chain Tctex-type 1 isoform X2
21.1	21.0	20.9	20.7	21.2	21.3	1.1145	0.8106	gi|635134123	isocitrate dehydrogenase [NAD] subunit alpha, mitochondrial
17.2	18.4	18.0	18.7	17.5	16.8	0.6742	0.8109	gi|635039348	platelet-activating factor acetylhydrolase IB subunit gamma
20.7	20.1	20.9	20.8	19.7	20.8	0.7713	0.8113	gi|635103716	astrocytic phosphoprotein PEA-15
24.7	25.4	24.5	24.5	25.2	24.7	0.8203	0.8130	gi|635066023	myosin light polypeptide 6
20.2	20.5	19.8	20.0	20.0	20.3	0.8826	0.8132	gi|635141789	protein arginine N-methyltransferase 5 isoform X3
19.6	19.8	18.8	18.8	19.9	19.2	0.7792	0.8137	gi|635077553	UPF0364 protein C6orf211 homolog
14.7	15.6	12.5	12.9	15.8	13.1	0.4663	0.8139	gi|635112820	splicing factor 3A subunit 3
22.5	22.3	22.1	22.2	22.3	22.6	1.1082	0.8141	gi|635148115	ADP/ATP translocase 3
20.4	20.3	19.6	20.0	20.2	20.2	1.1650	0.8152	gi|635142324	ras-related protein Rab-9A
20.2	20.2	19.5	19.3	20.7	20.3	1.3050	0.8175	gi|635115839	LSM8 homolog, U6 small nuclear RNA associated
19.8	21.5	18.1	20.1	20.3	19.8	1.7535	0.8176	gi|635111706	TAR DNA-binding protein 43
20.9	21.0	20.9	20.4	21.6	21.0	1.2211	0.8194	gi|635132761	sodium/potassium-transporting ATPase subunit beta-1
20.9	21.3	21.0	21.1	21.1	21.1	1.0647	0.8195	gi|635103664	coatomer subunit alpha isoform X2
19.6	19.9	19.3	19.4	19.6	19.6	0.8979	0.8195	gi|635076626	ethylmalonyl-CoA decarboxylase isoform X4
24.1	25.0	24.1	24.1	24.6	24.3	0.8332	0.8203	gi|635089946	nucleoside diphosphate kinase B isoform X1
22.7	23.1	22.5	23.0	22.4	22.8	0.8645	0.8205	gi|635016456	serpin H1 isoform X2
18.1	18.5	17.3	17.4	18.4	18.5	1.3183	0.8210	gi|635135860	COP9 signalosome complex subunit 2 isoform X2
20.4	21.8	21.0	21.0	21.4	21.1	1.2625	0.8215	gi|635105192	RNA-binding protein 8A isoform X2
25.8	26.1	25.2	26.0	25.9	25.4	1.2020	0.8216	gi|635128768	calmodulin
18.1	18.6	17.7	18.0	18.5	18.2	1.1767	0.8217	gi|635133894	isoleucine--tRNA ligase, mitochondrial
18.3	19.7	18.5	18.6	19.4	18.8	1.3170	0.8218	gi|635135250	sorting nexin-1 isoform X4
19.2	20.1	20.1	18.9	20.4	19.8	0.7518	0.8219	gi|635024954	serine/threonine-protein kinase DCLK1 isoform X9
18.8	18.3	18.0	18.5	18.3	18.5	1.1438	0.8231	gi|635112764	serine--tRNA ligase, cytoplasmic
22.9	23.3	23.0	22.8	23.2	23.3	1.1165	0.8232	gi|635112985	T-complex protein 1 subunit zeta
27.1	27.0	26.7	26.8	27.1	26.9	1.0776	0.8234	gi|635096313	tubulin beta chain
19.8	19.9	19.5	19.9	20.0	19.4	1.1225	0.8245	gi|635043079	general vesicular transport factor p115 isoform X4
23.3	23.7	23.3	23.4	23.7	23.3	1.1086	0.8250	gi|635102544	X-ray repair cross-complementing protein 6

23.0	24.0	23.2	23.6	23.3	23.1	0.8372	0.8258	gi|635020678	14-3-3 protein beta/alpha
18.6	19.1	18.4	18.6	19.1	18.6	1.1475	0.8267	gi|635055159	myoferlin
19.2	21.7	21.3	20.8	20.9	21.1	1.5142	0.8270	gi|635109853	eukaryotic translation initiation factor 3 subunit I
22.3	22.9	21.2	21.1	22.5	22.3	0.6976	0.8271	gi|635083409	transformer-2 protein homolog beta isoform X2
20.1	20.1	19.7	19.6	20.0	20.1	0.8931	0.8272	gi|635140582	eukaryotic translation initiation factor 3 subunit B
19.0	18.2	17.7	18.1	18.4	18.1	0.8124	0.8278	gi|635091067	importin subunit alpha-1
17.2	17.8	16.8	16.1	17.7	17.6	0.7281	0.8286	gi|635106512	protein Dr1
22.5	23.2	21.2	22.4	22.9	22.0	1.4109	0.8287	gi|635034731	polypyrimidine tract-binding protein 1 isoform X4
19.1	18.9	17.9	17.9	19.0	18.6	0.7721	0.8292	gi|635011139	toll-interacting protein
24.3	25.3	24.5	24.6	24.8	24.5	0.8559	0.8293	gi|635036434	calreticulin
24.6	25.3	24.8	24.5	25.1	24.9	0.8606	0.8305	gi|635087440	eukaryotic translation initiation factor 5A-1 isoform X2
22.2	22.7	22.3	22.4	22.5	22.3	0.9143	0.8306	gi|635051288	6-phosphofructokinase type C
19.5	19.2	18.2	18.5	19.5	19.1	1.2865	0.8312	gi|635014034	estradiol 17-beta-dehydrogenase 12
19.6	19.3	18.5	18.7	19.4	19.6	1.2336	0.8313	gi|635135073	very-long-chain (3R)-3-hydroxyacyl-[acyl-carrier protein] dehydratase 3
17.6	20.7	18.6	18.0	20.4	19.4	1.8257	0.8315	gi|635131321	small nuclear ribonucleoprotein E isoform X2
11.1	11.5	11.0	10.6	11.7	11.6	1.2227	0.8315	gi|635112818	microtubule-actin cross-linking factor 1
17.7	17.7	17.9	16.8	18.5	18.3	1.3217	0.8324	gi|635078401	U5 small nuclear ribonucleoprotein 200 kDa helicase
16.7	18.0	16.3	16.4	17.6	17.4	1.3749	0.8324	gi|635073810	GTP:AMP phosphotransferase AK3, mitochondrial isoform X4
20.9	21.7	20.2	20.8	21.6	20.8	1.3072	0.8325	gi|635125721	heterogeneous nuclear ribonucleoprotein A/B-like
19.9	20.5	20.4	20.1	20.6	20.2	1.1354	0.8330	gi|635126974	ERO1-like protein alpha isoform X2
21.0	22.1	21.1	21.2	21.5	21.1	0.8208	0.8331	gi|635033916	lysine--tRNA ligase isoform X2
19.9	21.9	21.5	20.9	20.6	21.4	0.7165	0.8339	gi|635125502	clathrin light chain B
21.2	21.5	21.1	21.2	21.5	21.2	1.0790	0.8341	gi|635032154	vacuolar protein sorting-associated protein 35 isoform X2
20.5	20.9	20.2	20.2	20.7	20.6	0.8742	0.8342	gi|635137969	phosphoglucomutase-2
19.5	19.5	19.1	19.3	19.6	19.1	0.9027	0.8352	gi|635099726	ubiquitin carboxyl-terminal hydrolase 14 isoform X2
18.9	19.1	18.3	18.1	19.1	18.9	0.8204	0.8354	gi|635107325	serine/arginine-rich splicing factor 11 isoform X4
23.2	23.7	22.5	23.1	23.3	22.7	0.8139	0.8359	gi|635058930	heterogeneous nuclear ribonucleoprotein A3
23.0	23.2	22.7	23.2	23.1	22.7	1.1055	0.8364	gi|635108941	nuclease-sensitive element-binding protein 1 isoform X1
27.1	27.4	27.0	27.1	27.0	27.3	0.9259	0.8376	gi|635135402	annexin A2
17.4	17.4	16.1	16.4	17.8	17.2	1.3503	0.8379	gi|635103407	UDP-N-acetylhexosamine pyrophosphorylase isoform X5
18.5	18.3	18.3	18.2	18.6	18.4	1.0766	0.8383	gi|635094615	CD2-associated protein isoform X2
20.2	19.8	19.5	19.9	19.4	20.0	0.8714	0.8384	gi|635141713	FACT complex subunit SPT16
16.1	16.2	15.1	15.4	16.4	15.9	1.2539	0.8393	gi|635029477	leucyl-cystinyl aminopeptidase
17.5	17.7	17.3	17.3	17.8	17.5	1.1008	0.8397	gi|635088250	developmentally-regulated GTP-binding protein 2
22.2	22.3	22.1	22.1	22.4	22.0	0.9291	0.8404	gi|635057282	translin isoform X1
22.2	22.5	22.2	22.1	22.8	22.2	1.1203	0.8416	gi|635015756	26S proteasome non-ATPase regulatory subunit 13
20.3	22.9	22.1	22.0	22.2	21.5	1.4826	0.8420	gi|635105025	proteasome subunit beta type-4
23.7	24.2	23.7	23.6	24.0	23.8	0.9067	0.8433	gi|635067296	ubiquitin-conjugating enzyme E2 N
20.9	21.4	20.4	20.5	21.3	21.1	1.2018	0.8435	gi|635055905	alpha-centractin
23.5	24.1	23.7	23.7	23.9	23.6	0.9160	0.8441	gi|635012908	elongation factor 1-gamma
18.7	18.6	18.4	18.3	18.6	18.6	0.9396	0.8443	gi|635011864	serine/threonine-protein phosphatase PP1-alpha catalytic subunit

21.3	22.6	21.9	21.4	22.0	22.0	0.8144	0.8444	gi|635036188	glucosidase 2 subunit beta isoform X2
21.6	21.7	20.6	21.3	21.2	21.3	0.8446	0.8458	gi|635018094	platelet-activating factor acetylhydrolase IB subunit beta isoform X2
25.8	25.7	25.2	25.4	25.6	25.5	0.9196	0.8460	gi|635011771	glutathione S-transferase P isoform X2
21.8	22.4	22.0	22.4	21.9	22.1	1.1150	0.8469	gi|635039836	small nuclear ribonucleoprotein Sm D2
24.6	25.7	23.8	24.8	25.5	24.3	1.3609	0.8475	gi|635065817	heterogeneous nuclear ribonucleoprotein A1 isoform X2
17.0	17.9	18.3	17.2	18.1	18.1	1.2543	0.8481	gi|635081066	protein phosphatase 1B isoform X5
16.1	16.6	15.8	16.0	16.3	16.3	1.1216	0.8481	gi|635035597	cdc42-interacting protein 4 isoform X4
19.3	20.9	20.1	19.5	20.3	20.3	0.7837	0.8484	gi|635029829	SUMO-conjugating enzyme UBC9
19.7	20.0	19.8	20.1	19.8	19.4	0.9018	0.8486	gi|635134356	cytochrome c oxidase subunit 5A, mitochondrial
20.7	21.2	19.5	20.2	20.8	20.0	0.7634	0.8490	gi|635026919	lysosome-associated membrane glycoprotein 1
22.3	22.7	22.8	22.7	22.3	22.9	1.1053	0.8500	gi|635105349	D-3-phosphoglycerate dehydrogenase
23.1	23.6	23.0	23.1	23.4	23.3	1.1084	0.8507	gi|635121096	ras-related protein Rab-7a
23.6	23.7	23.3	23.5	23.6	23.5	0.9481	0.8509	gi|635112732	putative protein FAM10A4
21.7	22.6	22.1	22.2	22.3	21.7	0.8615	0.8513	gi|635012770	reticulon-3 isoform X5
22.9	23.4	23.0	23.6	22.8	22.7	0.8592	0.8514	gi|635020930	60S ribosomal protein L6
19.8	19.7	19.0	19.5	19.6	19.5	1.1312	0.8518	gi|635083421	insulin-like growth factor 2 mRNA-binding protein 2 isoform X6
21.1	21.3	20.7	20.7	21.2	21.0	0.8982	0.8520	gi|635022798	trifunctional purine biosynthetic protein adenosine-3
21.0	21.7	21.3	21.5	21.4	21.0	0.8837	0.8521	gi|635092643	26S proteasome non-ATPase regulatory subunit 3
21.3	20.8	20.1	20.4	20.7	21.3	1.2154	0.8528	gi|635065098	tubulin alpha-1C chain
21.1	21.3	20.8	20.8	21.4	21.1	1.1118	0.8531	gi|635015028	coatomer subunit beta
20.8	21.1	20.6	20.5	21.2	20.6	0.8894	0.8537	gi|635038773	26S proteasome non-ATPase regulatory subunit 8
21.2	21.5	20.9	21.1	21.5	21.2	1.0985	0.8543	gi|635040342	U1 small nuclear ribonucleoprotein 70 kDa isoform X2
17.8	18.0	17.0	17.4	17.9	17.7	1.1710	0.8543	gi|635068638	translational activator GCN1
11.5	16.1	12.4	12.5	16.0	12.7	2.2738	0.8544	gi|635029168	ras GTPase-activating protein 1 isoform X2
21.2	21.4	21.0	20.9	21.4	21.3	1.0914	0.8548	gi|635118489	microtubule-associated protein 4 isoform X16
15.5	16.6	16.1	15.8	15.9	16.8	1.2202	0.8551	gi|635084253	structural maintenance of chromosomes protein 4 isoform X2
21.5	22.0	21.1	21.4	21.5	21.5	0.8798	0.8555	gi|635102245	probable ATP-dependent RNA helicase DDX17 isoform X2
19.2	19.5	18.7	19.3	19.3	18.7	0.8747	0.8560	gi|635031255	cytochrome b-c1 complex subunit 2, mitochondrial
20.6	21.5	21.6	21.4	21.5	20.9	1.1809	0.8566	gi|635031953	RNA-binding protein FUS isoform X2
23.0	24.1	23.2	22.6	24.0	23.3	0.7861	0.8581	gi|635041590	60S ribosomal protein L28
21.6	22.6	22.1	22.5	21.9	21.8	0.8490	0.8583	gi|635098498	thioredoxin-like protein 1 isoform X2
12.2	14.6	14.2	12.9	14.3	14.2	1.4653	0.8584	gi|635104237	ras GTPase-activating-like protein IQGAP3 isoform X3
19.2	20.0	18.3	18.9	19.3	19.1	0.8030	0.8584	gi|635093642	COP9 signalosome complex subunit 1 isoform X9
22.1	22.6	21.6	22.0	22.4	22.0	1.1455	0.8619	gi|635132088	ATP-dependent RNA helicase A isoform X1
21.2	21.0	20.7	20.5	21.6	21.1	1.1688	0.8626	gi|635139994	28 kDa heat- and acid-stable phosphoprotein
16.3	19.3	18.2	18.1	18.0	18.2	1.4567	0.8634	gi|635077026	tyrosine-protein kinase Fyn isoform X3
23.2	23.7	23.0	23.1	23.4	23.3	0.9131	0.8639	gi|635054352	inorganic pyrophosphatase
17.4	17.7	17.0	16.7	17.8	17.6	0.8465	0.8640	gi|635132243	torsin-1A-interacting protein 2
20.9	22.1	21.2	20.7	21.6	21.6	0.8192	0.8646	gi|635093341	eukaryotic initiation factor 4A-III
22.7	23.0	22.5	22.3	22.8	23.0	0.9036	0.8649	gi|635021048	dolichyl-diphosphooligosaccharide--protein glycosyltransferase subunit 2 isoform X3
19.9	20.2	19.5	19.4	20.2	19.8	0.8800	0.8652	gi|635091808	116 kDa U5 small nuclear ribonucleoprotein component

15.7	15.1	15.5	14.5	15.6	16.4	1.2771	0.8652	gi|635066796	rab-3A-interacting protein isoform X5
23.8	23.7	23.0	23.2	23.8	23.7	1.1363	0.8659	gi|635066204	prostaglandin E synthase 3 isoform X2
25.5	25.5	25.2	25.2	25.5	25.5	0.9514	0.8665	gi|635082618	protein disulfide-isomerase A6 isoform X2
21.9	20.2	20.0	20.4	20.3	21.7	1.3636	0.8666	gi|635139078	ATP synthase subunit e, mitochondrial
17.8	17.6	16.4	17.0	17.5	17.6	1.2259	0.8667	gi|635057257	myc box-dependent-interacting protein 1 isoform X16
17.0	18.6	17.6	17.1	17.8	18.6	1.3006	0.8682	gi|635091346	coiled-coil domain-containing protein 47 isoform X4
22.5	23.1	22.5	22.5	22.8	22.8	1.0935	0.8691	gi|635113900	glycine--tRNA ligase
15.8	15.2	15.0	15.2	16.2	14.9	1.2025	0.8695	gi|635127171	exocyst complex component 5 isoform X1
18.8	18.8	18.0	18.4	18.6	18.8	1.1179	0.8696	gi|635076689	HD domain-containing protein 2
20.0	20.4	19.6	19.5	20.1	20.3	0.8758	0.8702	gi|635112077	cytosolic acyl coenzyme A thioester hydrolase isoform X5
22.0	23.0	21.0	21.9	22.8	21.0	0.7355	0.8713	gi|635109561	splicing factor, proline- and glutamine-rich isoform X2
17.0	18.5	18.0	17.7	17.9	17.9	0.8377	0.8715	gi|635061841	leucine-rich repeat flightless-interacting protein 1 isoform X22
19.6	21.5	20.8	20.4	21.2	20.5	1.2557	0.8723	gi|635054561	heterogeneous nuclear ribonucleoprotein H3 isoform X4
24.1	25.0	24.4	24.2	24.8	24.6	1.1352	0.8724	gi|635093554	rho GDP-dissociation inhibitor 1 isoform X2
16.4	18.0	17.5	16.6	17.2	17.8	0.7961	0.8730	gi|635139608	procollagen-lysine,2-oxoglutarate 5-dioxygenase 3 isoform X1
18.7	18.9	18.6	18.4	19.1	18.7	0.9160	0.8738	gi|635070805	spectrin alpha chain, non-erythrocytic 1 isoform X5
19.2	18.9	18.5	18.2	19.3	18.9	0.8638	0.8744	gi|635113023	lanC-like protein 2 isoform X2
18.6	19.2	18.8	18.2	19.0	19.5	1.1643	0.8750	gi|635054084	serine/threonine-protein phosphatase 2B catalytic subunit beta isoform isoform X5
25.0	24.9	24.8	24.6	25.0	25.2	0.9352	0.8752	gi|635059587	10 kDa heat shock protein, mitochondrial isoform X4
14.0	13.8	13.5	13.5	14.0	13.9	1.0917	0.8764	gi|635111139	E3 ubiquitin-protein ligase UBR4 isoform X8
23.5	23.7	23.0	23.2	23.6	23.4	1.0985	0.8768	gi|635095703	40S ribosomal protein S10
18.0	18.5	17.4	18.1	18.3	17.8	1.1431	0.8769	gi|635087799	myosin-10 isoform X9
15.9	16.3	15.9	15.5	16.6	16.2	1.1388	0.8769	gi|635056632	short/branched chain specific acyl-CoA dehydrogenase, mitochondrial
26.5	27.2	27.1	26.9	27.0	26.7	0.9081	0.8769	gi|635051897	vimentin
20.8	21.6	21.0	21.0	21.4	21.0	1.1173	0.8774	gi|635015439	eukaryotic translation initiation factor 3 subunit F
26.3	27.0	26.9	26.7	26.8	26.8	1.0882	0.8776	gi|635083879	thioredoxin
16.8	16.2	15.9	15.8	16.4	16.8	1.1538	0.8781	gi|635048998	copine-3
20.5	20.6	19.8	20.2	20.2	20.3	0.9129	0.8789	gi|635098014	cytosolic non-specific dipeptidase
17.0	16.8	16.5	16.5	17.0	16.8	0.9302	0.8792	gi|635126095	importin-4
21.1	21.0	20.5	20.9	21.1	20.6	0.9141	0.8795	gi|67082898	ATPase6
16.5	16.5	16.0	16.1	16.4	16.7	1.0871	0.8798	gi|635104338	28S ribosomal protein S29, mitochondrial isoform X5
19.0	17.2	18.4	18.4	18.2	18.3	1.2236	0.8809	gi|635036781	ras-related protein Rab-8A
18.7	19.8	18.3	19.1	19.3	18.7	1.1847	0.8816	gi|635091642	integrin beta-3 isoform X2
23.8	24.6	24.8	24.0	24.7	24.6	1.1429	0.8831	gi|635015383	60S ribosomal protein L27a
21.0	20.9	20.4	20.6	20.8	20.8	0.9317	0.8832	gi|635119802	pyruvate dehydrogenase E1 component subunit beta, mitochondrial
20.0	19.8	18.9	19.2	20.1	19.1	0.8402	0.8834	gi|635123897	heterogeneous nuclear ribonucleoprotein A0
17.5	18.7	17.3	17.0	18.4	18.4	1.2630	0.8840	gi|635081909	microtubule-associated protein RP/EB family member 3 isoform X2
15.9	14.8	14.9	15.0	15.2	15.6	1.1491	0.8846	gi|635086860	clustered mitochondria protein homolog isoform X5
20.4	21.5	20.2	20.7	21.5	20.2	1.2179	0.8852	gi|635138151	putative pre-mRNA-splicing factor ATP-dependent RNA helicase DHX15
23.9	24.3	23.9	23.8	24.2	24.1	1.0697	0.8857	gi|635089580	clathrin heavy chain 1 isoform X2
14.4	15.3	14.8	14.9	15.7	14.2	1.1905	0.8858	gi|635067276	early endosome antigen 1

17.4	19.8	18.1	19.1	18.2	18.3	1.3227	0.8858	gi|635037967	SUMO-activating enzyme subunit 2 isoform X2
18.2	18.5	17.8	18.1	18.2	18.3	1.0840	0.8866	gi|635100241	nucleoporin SEH1 isoform X3
17.4	18.0	16.6	18.0	17.5	16.3	0.7954	0.8870	gi|635054055	protein transport protein Sec24C isoform X3
25.3	25.4	24.6	24.8	25.3	25.4	1.1082	0.8884	gi|635068640	60S acidic ribosomal protein P0
24.9	24.8	24.5	24.6	24.8	24.7	0.9577	0.8902	gi|635131967	peptidyl-prolyl cis-trans isomerase B
23.0	24.0	23.3	23.2	23.5	23.4	0.9084	0.8911	gi|635019794	proteasome subunit alpha type-7
20.4	20.3	18.8	19.7	20.1	19.9	1.1907	0.8915	gi|635066751	cleavage and polyadenylation specificity factor subunit 6 isoform X4
17.3	18.7	14.7	15.6	18.6	15.9	0.6096	0.8935	gi|635123572	septin-8 isoform X7
22.5	23.9	23.5	23.2	23.2	23.3	0.8745	0.8936	gi|635024322	60S ribosomal protein L29-like, partial
21.6	22.3	21.5	22.0	22.0	21.6	1.1005	0.8942	gi|635137664	transcription factor BTF3
15.8	15.0	14.5	14.6	16.1	14.4	0.8081	0.8946	gi|635028233	superkiller viralicidic activity 2-like 2
21.8	22.2	21.6	21.5	22.0	22.0	0.9283	0.8958	gi|635015295	importin-7 isoform X2
18.7	19.6	19.3	19.3	19.3	19.0	0.9081	0.8967	gi|635049713	eukaryotic translation initiation factor 3 subunit E isoform X1
19.2	19.1	18.6	18.7	19.2	19.0	1.0796	0.8973	gi|635094501	DNA replication licensing factor MCM3 isoform X1
21.3	22.8	22.2	21.9	22.3	22.0	0.8691	0.8975	gi|635051535	ATP synthase subunit gamma, mitochondrial isoform X2
22.9	22.5	21.8	22.3	22.5	22.5	1.1012	0.8975	gi|635146311	translocon-associated protein subunit delta
23.4	24.7	24.1	24.0	24.2	24.3	1.1242	0.8982	gi|635039265	40S ribosomal protein S19
19.4	19.3	18.5	18.9	19.5	19.0	1.1115	0.8987	gi|635045872	signal peptidase complex subunit 3
21.3	22.1	21.8	21.9	21.7	21.6	0.9252	0.8987	gi|635125595	vesicular integral-membrane protein VIP36
19.5	20.1	19.4	19.6	19.7	19.7	0.9365	0.8987	gi|635120073	succinyl-CoA ligase [GDP-forming] subunit beta, mitochondrial isoform X1
19.2	21.2	19.4	20.2	20.4	19.0	0.7884	0.8992	gi|635146664	non-POU domain-containing octamer-binding protein
18.8	18.8	18.3	18.1	18.3	19.7	1.1800	0.9001	gi|635070587	argininosuccinate synthase isoform X2
22.9	24.4	24.6	23.0	24.7	24.4	1.2428	0.9019	gi|635096864	histone H3.1-like
20.7	20.9	19.9	20.2	20.8	20.7	1.1142	0.9031	gi|635023804	pyridoxal kinase isoform X2
23.3	23.8	23.3	23.3	23.6	23.4	0.9445	0.9054	gi|635027385	T-complex protein 1 subunit epsilon isoform X1
16.1	16.2	15.8	14.8	17.0	16.1	0.8269	0.9056	gi|635080131	nucleolysin TIA-1 isoform p40 isoform X3
26.4	26.6	26.0	26.0	26.7	26.2	0.9287	0.9056	gi|635080675	ubiquitin-40S ribosomal protein S27a
21.2	21.3	20.6	20.6	21.3	21.3	1.1015	0.9063	gi|635124018	matrin-3 isoform X2
19.5	20.2	18.9	19.4	19.7	19.3	0.8906	0.9067	gi|635071961	polypyrimidine tract-binding protein 3 isoform X12
21.5	21.6	20.9	20.7	21.7	21.5	0.8973	0.9085	gi|635074604	serine palmitoyltransferase 1
19.2	19.1	18.9	18.8	19.4	19.1	1.0648	0.9087	gi|635102037	eukaryotic translation initiation factor 3 subunit D
19.5	19.0	19.0	19.1	19.5	18.9	0.9310	0.9092	gi|635144515	SH3 domain-binding glutamic acid-rich-like protein
16.3	16.9	16.0	16.2	17.1	15.8	0.8813	0.9101	gi|635026577	KDEL motif-containing protein 1 isoform X4
17.7	18.3	17.2	17.3	18.1	17.9	1.1193	0.9102	gi|635072000	proteasome-associated protein ECM29 homolog isoform X2
21.3	21.9	21.0	21.1	21.5	21.4	0.9222	0.9103	gi|635035577	tubulin beta-4A chain
23.6	23.8	22.8	23.8	23.1	23.4	1.1079	0.9105	gi|635128370	activator of 90 kDa heat shock protein ATPase homolog 1
22.8	23.6	22.9	22.4	23.3	23.5	0.8898	0.9107	gi|635012126	barrier-to-autointegration factor
18.3	19.5	18.8	19.3	18.2	19.0	0.8737	0.9107	gi|332268712	MHC class IA antigen
17.6	17.1	16.2	16.3	17.2	17.7	1.1682	0.9113	gi|635018414	porphobilinogen deaminase isoform X2
20.9	22.0	21.2	21.3	21.9	21.0	1.1172	0.9121	gi|635108082	protein mago nashi homolog
22.6	22.7	22.2	22.3	22.6	22.4	0.9504	0.9121	gi|635092873	puromycin-sensitive aminopeptidase isoform X1

14.4	14.8	13.9	13.6	15.0	14.2	0.8786	0.9131	gi|635031106	UPF0505 protein C16orf62 homolog isoform X2
19.8	19.5	18.2	20.1	20.0	17.7	1.2789	0.9140	gi|635095493	serine/arginine-rich splicing factor 3
22.3	22.8	22.4	22.4	22.7	22.4	0.9538	0.9149	gi|635127756	alpha-actinin-1 isoform X2
20.0	20.0	19.5	19.5	19.9	20.1	1.0678	0.9163	gi|635043189	septin-11 isoform X4
17.9	16.4	18.9	19.0	17.1	17.4	1.2746	0.9165	gi|635050135	protein FAM49B isoform X1
19.7	20.1	18.9	19.0	20.0	19.8	1.1243	0.9174	gi|635012486	splicing factor 1 isoform X9
21.7	21.5	21.8	21.9	21.6	21.5	1.0330	0.9191	gi|635022786	ATP synthase subunit O, mitochondrial
17.0	17.6	16.5	16.5	17.5	17.0	0.8965	0.9201	gi|635067235	plasma membrane calcium-transporting ATPase 1 isoform X3
21.2	22.2	21.6	21.4	22.0	21.7	1.0851	0.9202	gi|635012887	neutral alpha-glucosidase AB
24.0	24.3	23.6	23.6	24.3	23.9	0.9360	0.9223	gi|635087218	complement component 1 Q subcomponent-binding protein, mitochondrial
16.0	16.0	15.3	15.5	16.0	16.0	1.0704	0.9224	gi|635061311	E3 ubiquitin-protein ligase TRIP12 isoform X9
18.7	18.7	17.9	18.2	18.6	18.6	1.0719	0.9227	gi|635137527	OCIA domain-containing protein 2 isoform X1
18.9	19.1	17.6	17.9	19.2	18.7	1.1549	0.9230	gi|635133178	translin-associated protein X
14.6	15.1	13.8	14.7	16.0	12.5	0.7749	0.9240	gi|635018987	signal recognition particle receptor subunit alpha isoform X2
18.2	18.1	17.4	17.2	18.2	18.4	1.1078	0.9249	gi|635102724	tubulin--tyrosine ligase-like protein 12 isoform X2
15.9	16.2	15.3	15.0	16.4	15.9	0.8950	0.9264	gi|635100081	serine/threonine-protein phosphatase 4 regulatory subunit 1 isoform X4
17.7	18.7	17.9	16.9	18.8	18.8	1.1699	0.9264	gi|635133277	mitochondrial import receptor subunit TOM20 homolog isoform X2
20.0	20.3	18.2	19.4	19.7	19.6	1.1614	0.9265	gi|635043422	COP9 signalosome complex subunit 4 isoform X2
21.0	21.6	21.2	21.2	21.3	21.3	1.0409	0.9268	gi|635126983	26S protease regulatory subunit 10B
19.8	19.5	19.0	18.7	19.7	19.8	0.9144	0.9283	gi|635066866	ras-related protein Rab-21
18.0	17.5	17.1	17.6	19.1	15.6	0.7933	0.9285	gi|635020988	protein-glutamine gamma-glutamyltransferase 2
16.2	17.3	16.7	16.2	17.2	16.6	0.9118	0.9286	gi|635111146	delta-1-pyrroline-5-carboxylate dehydrogenase, mitochondrial
23.7	24.6	24.1	24.2	24.3	23.9	1.0666	0.9290	gi|635106546	60S ribosomal protein L5 isoform X2
16.0	16.0	16.0	15.9	16.0	16.1	1.0162	0.9295	gi|635040128	DNA ligase 1 isoform X3
22.1	22.6	21.6	22.0	22.5	21.9	1.0781	0.9300	gi|635104748	interleukin enhancer-binding factor 2 isoform X3
18.0	16.4	15.0	17.4	17.7	14.7	1.3128	0.9302	gi|635123206	peroxisomal multifunctional enzyme type 2
18.0	18.3	17.4	17.5	18.3	17.9	0.9272	0.9305	gi|635111721	DNA fragmentation factor subunit alpha
19.0	19.4	18.3	18.1	18.9	19.6	0.8928	0.9310	gi|635107403	plasminogen activator inhibitor 1 RNA-binding protein isoform X4
19.8	20.2	19.6	20.1	19.8	19.8	1.0465	0.9313	gi|635123916	eukaryotic peptide chain release factor subunit 1
22.3	22.8	22.0	22.2	22.3	22.5	0.9468	0.9316	gi|635020322	exportin-2
18.7	18.6	18.3	18.3	18.8	18.6	1.0415	0.9323	gi|635125953	alpha-aminoadipic semialdehyde dehydrogenase
20.5	20.9	20.2	20.4	20.6	20.6	1.0437	0.9323	gi|635130447	bifunctional glutamate/proline--tRNA ligase isoform X2
20.5	21.2	19.5	19.8	20.6	20.6	0.8892	0.9326	gi|635012628	peptidyl-prolyl cis-trans isomerase FKBP2 isoform X4
23.7	23.9	22.7	23.5	23.6	23.2	1.0869	0.9332	gi|635141695	heterogeneous nuclear ribonucleoproteins C1/C2 isoform X3
18.5	17.7	17.7	17.8	18.1	18.1	1.0622	0.9339	gi|635114655	asparagine synthetase [glutamine-hydrolyzing] isoform X2
21.3	21.5	20.6	21.0	21.2	21.3	1.0583	0.9340	gi|635013637	26S protease regulatory subunit 6A isoform X2
17.6	18.8	14.4	17.5	18.3	15.4	1.3673	0.9343	gi|635054934	bifunctional 3-phosphoadenosine 5-phosphosulfate synthase 2 isoform X3
17.0	16.8	15.7	15.8	17.3	16.6	1.1258	0.9345	gi|635123500	prolyl 4-hydroxylase subunit alpha-2 isoform X4
21.1	22.2	21.8	21.7	22.1	21.4	1.0795	0.9346	gi|635115394	26S protease regulatory subunit 7
23.7	23.5	23.2	23.3	23.5	23.6	0.9677	0.9358	gi|635065704	poly(rC)-binding protein 2 isoform X19
23.4	23.9	22.6	23.1	23.9	23.0	1.0992	0.9360	gi|635114128	heterogeneous nuclear ribonucleoproteins A2/B1 isoform X3

21.7	22.0	21.3	21.6	22.0	21.5	1.0499	0.9360	gi|635066667	cullin-associated NEDD8-dissociated protein 1
19.7	20.6	20.1	20.2	20.3	19.8	0.9454	0.9369	gi|635096078	valine--tRNA ligase isoform X2
18.6	19.5	18.4	18.3	19.4	18.6	0.9099	0.9369	gi|635126823	ADP-ribosylation factor 6
17.1	17.3	16.2	17.4	16.2	16.8	0.9068	0.9369	gi|635106332	RNA 3-terminal phosphate cyclase isoform X2
17.3	17.7	16.8	17.0	17.4	17.3	1.0551	0.9373	gi|635115063	multidrug resistance protein 1 isoform X3
23.2	23.2	22.6	22.8	23.3	22.9	1.0500	0.9383	gi|635138493	WD repeat-containing protein 1
16.7	17.4	16.4	16.5	17.0	17.1	1.0683	0.9387	gi|635101928	target of Myb protein 1 isoform X5
18.7	18.9	17.8	18.1	18.8	18.5	1.0780	0.9389	gi|635016015	ribonucleoside-diphosphate reductase large subunit isoform X1
15.9	17.6	16.7	16.8	17.3	16.2	1.1136	0.9395	gi|635092256	tubulin gamma-1 chain
17.5	18.2	17.4	17.7	17.1	18.1	0.9312	0.9397	gi|635122361	mitochondrial import receptor subunit TOM70
23.3	22.8	23.1	22.9	23.1	23.2	1.0317	0.9403	gi|635077865	T-complex protein 1 subunit alpha
22.3	22.4	21.8	22.3	22.1	22.3	1.0348	0.9411	gi|635076960	myristoylated alanine-rich C-kinase substrate isoform X1
20.3	20.5	19.9	20.3	20.3	20.1	1.0338	0.9422	gi|635111115	aflatoxin B1 aldehyde reductase member 2 isoform X1
20.4	20.9	19.1	20.2	20.8	19.5	1.1191	0.9424	gi|635037600	heterogeneous nuclear ribonucleoprotein M
21.1	21.1	20.5	21.0	21.1	20.5	0.9520	0.9434	gi|635074614	isoleucine--tRNA ligase, cytoplasmic
27.6	27.5	27.2	27.5	27.6	27.3	1.0278	0.9438	gi|635062941	glyceraldehyde-3-phosphate dehydrogenase
17.5	19.2	18.4	17.9	19.1	18.3	1.1048	0.9440	gi|635123905	catenin alpha-1 isoform X2
19.6	20.6	19.9	19.8	20.5	19.9	1.0601	0.9441	gi|635080227	glutamine--fructose-6-phosphate aminotransferase [isomerizing] 1 isoform X3
22.0	22.5	22.2	22.3	22.2	22.2	0.9772	0.9465	gi|635101816	14-3-3 protein eta isoform X2
20.6	21.1	20.3	20.2	21.1	20.7	0.9423	0.9471	gi|635123658	S-phase kinase-associated protein 1
19.1	18.9	19.3	18.8	20.1	18.5	1.0838	0.9472	gi|635114810	cyclin-dependent kinase 6
18.2	17.9	17.3	17.6	17.9	18.0	0.9542	0.9494	gi|635081775	nuclear receptor-binding protein isoform X2
20.3	20.2	19.9	20.0	20.5	19.9	1.0351	0.9511	gi|635054477	nucleolar RNA helicase 2
21.7	21.6	20.9	21.3	21.5	21.4	1.0390	0.9511	gi|635143073	ATP-dependent RNA helicase DDX3X isoform X2
24.6	23.8	24.1	24.3	23.9	24.2	0.9605	0.9514	gi|635116388	aldo-keto reductase family 1 member B10-like isoform X2
21.3	22.6	22.2	22.4	22.0	21.7	1.0689	0.9517	gi|635025887	high mobility group protein B1-like
22.4	22.4	21.7	22.1	22.2	22.1	0.9659	0.9539	gi|635031561	elongation factor Tu, mitochondrial isoform X1
22.6	22.9	22.2	22.5	22.9	22.2	0.9600	0.9539	gi|635142791	peroxiredoxin-4 isoform X3
20.9	21.9	21.2	21.4	21.1	21.4	0.9589	0.9548	gi|51863477	glutamate dehydrogenase
22.2	22.3	21.9	22.0	22.2	22.2	0.9824	0.9548	gi|635121099	dolichyl-diphosphooligosaccharide--protein glycosyltransferase subunit 1
26.3	26.6	26.7	26.3	26.5	26.8	0.9732	0.9550	gi|635057707	pyruvate kinase PKM
17.7	17.9	17.2	17.0	17.9	17.8	0.9519	0.9552	gi|635084409	GMP synthase [glutamine-hydrolyzing] isoform X2
22.1	22.7	22.6	22.5	22.4	22.6	1.0273	0.9555	gi|635148055	60S ribosomal protein L3-like
22.7	23.4	22.3	22.5	23.2	22.8	1.0536	0.9558	gi|635126093	proteasome activator complex subunit 2
20.9	20.8	19.8	20.5	20.7	20.4	1.0503	0.9562	gi|635057043	ADP-ribosylation factor-like protein 3, partial
25.5	25.5	24.8	25.2	25.4	25.2	1.0329	0.9564	gi|635074112	retinal dehydrogenase 1 isoform X2
20.8	22.1	21.3	21.4	21.8	21.0	0.9419	0.9565	gi|635036057	interleukin enhancer-binding factor 3 isoform X5
18.1	18.7	18.4	18.0	18.6	18.6	0.9648	0.9568	gi|635037644	atherin
23.6	24.0	23.4	23.6	23.7	23.7	1.0243	0.9569	gi|635038829	alpha-actinin-4 isoform X1
24.1	24.8	24.1	24.0	24.6	24.3	0.9640	0.9590	gi|635066933	nucleosome assembly protein 1-like 1 isoform X3
20.2	21.0	20.5	20.5	20.6	20.6	1.0297	0.9599	gi|635089234	AP-2 complex subunit beta isoform X2

20.2	20.5	19.9	20.2	20.4	20.0	1.0258	0.9601	gi|635129401	cytoplasmic dynein 1 heavy chain 1
23.3	24.1	23.8	23.5	24.0	23.7	0.9680	0.9614	gi|635034515	60S ribosomal protein L13
18.2	18.9	18.3	17.9	18.9	18.6	0.9571	0.9620	gi|635062207	vigilin
23.1	23.3	23.4	23.5	23.1	23.1	0.9798	0.9623	gi|635073382	40S ribosomal protein S6
20.8	23.3	20.8	22.5	22.7	19.9	1.1500	0.9625	gi|635022469	peptidyl-prolyl cis-trans isomerase FKBP1A isoform X2
24.3	22.6	22.4	22.6	23.3	23.6	1.0782	0.9639	gi|635136851	actin, alpha cardiac muscle 1
20.0	21.1	20.7	20.8	21.2	19.8	1.0561	0.9657	gi|635122334	protein TFG isoform X4
15.0	15.4	14.6	14.2	15.9	14.8	0.9430	0.9658	gi|635122941	peroxisomal NADH pyrophosphatase NUDT12
20.7	20.9	20.3	20.4	20.9	20.6	1.0251	0.9674	gi|635138678	grpE protein homolog 1, mitochondrial
19.3	19.6	18.8	19.0	19.2	19.5	1.0262	0.9675	gi|635019776	proteasomal ubiquitin receptor ADRM1 isoform X2
18.8	18.6	18.1	18.4	18.4	18.6	0.9791	0.9676	gi|635139445	NADPH--cytochrome P450 reductase isoform X4
21.0	21.0	19.9	20.2	21.3	20.3	0.9523	0.9676	gi|635030736	calcium-regulated heat stable protein 1 isoform X2
21.5	22.1	21.6	21.6	21.8	21.8	0.9827	0.9685	gi|635133457	fumarate hydratase, mitochondrial
18.6	19.6	19.4	19.5	19.0	19.1	1.0348	0.9698	gi|635085309	signal recognition particle receptor subunit beta
15.1	14.9	14.6	14.6	15.0	15.1	0.9807	0.9700	gi|635137065	tight junction protein ZO-1 isoform X7
22.2	22.8	22.2	22.1	22.4	22.7	0.9747	0.9700	gi|635069556	proliferation-associated protein 2G4
21.7	22.9	22.6	22.4	22.7	22.0	0.9641	0.9705	gi|635080709	reticulon-4 isoform X4
16.6	15.6	14.7	13.9	16.5	16.3	0.9131	0.9707	gi|635125506	FAS-associated factor 2 isoform X2
13.8	14.2	13.5	12.9	14.6	14.1	1.0517	0.9707	gi|635103147	plexin-B2
23.3	23.9	23.3	23.4	23.7	23.4	0.9813	0.9710	gi|635021208	proliferating cell nuclear antigen
17.0	16.8	16.3	16.3	17.0	16.8	0.9759	0.9711	gi|635027240	lysophosphatidylcholine acyltransferase 1
17.1	17.1	16.4	16.4	17.6	16.7	1.0370	0.9717	gi|635078596	beta-centractin
24.2	24.8	24.5	24.3	24.7	24.5	0.9833	0.9734	gi|635087547	eukaryotic initiation factor 4A-I
23.9	24.0	23.5	23.9	23.9	23.7	1.0130	0.9741	gi|635092880	importin subunit beta-1
16.6	19.1	18.9	18.1	18.2	18.3	1.0642	0.9750	gi|635039935	serine/threonine-protein phosphatase 5
23.8	24.2	23.5	23.9	23.9	23.8	1.0163	0.9751	gi|635060054	elongation factor 1-beta
18.6	18.2	17.4	17.9	18.2	18.0	0.9729	0.9752	gi|635100771	catechol O-methyltransferase isoform X2
24.5	24.9	24.1	24.5	24.5	24.5	1.0175	0.9755	gi|635037961	glucose-6-phosphate isomerase
21.1	21.0	20.4	20.6	21.2	20.8	1.0202	0.9759	gi|635147935	rab GDP dissociation inhibitor alpha
12.5	15.1	13.3	12.9	15.0	13.1	1.0793	0.9760	gi|635039688	cleft lip and palate transmembrane protein 1 isoform X2
19.5	20.5	19.6	19.6	20.3	19.7	0.9734	0.9768	gi|635027056	programmed cell death protein 6 isoform X2
16.9	17.2	17.0	17.0	18.9	15.2	1.0762	0.9772	gi|635116602	zinc finger CCCH-type antiviral protein 1-like
15.8	18.2	15.4	15.4	16.8	17.2	0.9312	0.9778	gi|635057488	ATP-dependent RNA helicase DDX18
19.5	20.1	19.1	19.4	19.8	19.7	1.0199	0.9784	gi|635059693	basic leucine zipper and W2 domain-containing protein 1 isoform X2
18.6	17.6	16.6	17.5	18.3	17.2	1.0450	0.9786	gi|635097408	eukaryotic translation elongation factor 1 epsilon-1 isoform X3
18.0	18.5	17.7	17.7	18.1	18.3	0.9807	0.9787	gi|635093852	ATP-dependent RNA helicase DDX42
17.9	19.3	17.9	18.2	18.2	18.6	0.9693	0.9789	gi|635140133	ER lumen protein retaining receptor 2 isoform X2
17.4	19.8	19.6	18.1	19.8	19.1	1.0617	0.9789	gi|635056636	mitotic checkpoint protein BUB3
18.3	19.7	19.0	18.9	19.3	18.7	1.0293	0.9792	gi|635136357	synaptosomal-associated protein 23
22.9	23.0	22.8	22.7	23.0	23.1	1.0091	0.9798	gi|635079932	T-complex protein 1 subunit eta isoform X1
20.4	19.3	20.4	20.3	19.2	20.6	1.0371	0.9800	gi|635139606	mitochondrial fission 1 protein isoform X2

22.2	22.4	21.4	21.7	22.4	21.9	0.9790	0.9800	gi|635067742	thioredoxin reductase 1, cytoplasmic isoform X6
18.0	18.6	18.2	18.6	18.1	18.1	0.9855	0.9804	gi|635011814	coronin-1B
21.2	21.1	20.7	20.9	21.1	21.0	1.0086	0.9807	gi|635058831	obg-like ATPase 1 isoform X2
19.9	19.9	19.2	19.4	19.7	20.1	1.0186	0.9812	gi|635067489	phosphate carrier protein, mitochondrial
19.7	19.1	20.3	19.5	19.8	19.8	0.9796	0.9815	gi|635021798	eukaryotic translation initiation factor 2 subunit 2 isoform X2
18.7	18.7	17.5	18.2	18.2	18.5	1.0210	0.9830	gi|635030156	phosphoglycolate phosphatase
22.0	22.5	21.4	21.8	22.1	22.0	0.9837	0.9833	gi|635087281	thioredoxin domain-containing protein 17
20.4	20.6	19.3	19.9	20.4	20.0	1.0216	0.9835	gi|635101885	tRNA-splicing ligase RtcB homolog
17.9	18.0	17.3	17.1	18.2	18.0	1.0213	0.9839	gi|635042254	mitochondrial import inner membrane translocase subunit TIM50
24.3	24.9	24.5	24.5	24.5	24.5	0.9918	0.9851	gi|635137874	UDP-glucose 6-dehydrogenase
25.4	25.8	25.5	25.6	25.6	25.6	0.9947	0.9851	gi|635063994	L-lactate dehydrogenase B chain
19.4	19.9	19.2	19.0	20.1	19.5	0.9833	0.9852	gi|635066350	methionine--tRNA ligase, cytoplasmic
22.3	23.6	23.0	23.0	23.3	22.7	1.0185	0.9852	gi|635021812	adenosylhomocysteinase isoform X1
22.4	22.9	22.3	22.5	22.7	22.4	0.9911	0.9858	gi|635050818	plectin isoform X26
21.1	21.0	20.2	20.2	21.5	20.6	1.0214	0.9858	gi|635114726	serum paraoxonase/arylesterase 2
22.1	22.2	21.7	21.9	22.2	21.9	0.9917	0.9860	gi|635123601	heat shock 70 kDa protein 4 isoform X1
22.9	23.4	22.7	23.1	23.1	22.9	1.0086	0.9861	gi|635146405	filamin-A isoform X2
16.5	19.2	18.8	19.0	16.3	19.1	0.9500	0.9867	gi|635093500	nuclear protein localization protein 4 homolog isoform X1
23.7	24.3	23.8	23.8	24.0	24.0	1.0078	0.9870	gi|635125789	calnexin
14.6	16.5	15.6	16.5	14.7	15.5	1.0289	0.9880	gi|635139694	serrate RNA effector molecule homolog isoform X5
19.9	20.0	19.2	19.5	19.6	20.0	1.0087	0.9904	gi|635056461	eukaryotic translation initiation factor 3 subunit A isoform X2
18.3	19.7	18.5	18.8	19.6	18.1	0.9823	0.9904	gi|635114253	transmembrane glycoprotein NMB
21.1	21.5	21.0	21.1	21.2	21.3	1.0045	0.9904	gi|635124468	leucine--tRNA ligase, cytoplasmic isoform X1
24.4	24.7	24.1	24.3	24.5	24.3	1.0051	0.9905	gi|635102017	myosin-9
16.6	16.7	15.8	16.3	16.4	16.3	0.9922	0.9910	gi|635121256	solute carrier family 41 member 3 isoform X2
22.3	22.2	21.3	21.9	22.0	21.9	0.9921	0.9914	gi|635019580	elongation factor 1-alpha 2
20.4	20.4	19.8	20.0	20.4	20.3	1.0056	0.9918	gi|635064503	dynamin-1-like protein isoform X8
17.7	18.1	17.3	17.6	17.9	17.7	0.9935	0.9919	gi|635146494	H/ACA ribonucleoprotein complex subunit 4
13.7	15.2	13.7	13.2	14.3	15.0	0.9829	0.9923	gi|635080906	DNA mismatch repair protein Msh2
16.9	17.6	17.0	16.5	17.7	17.4	1.0094	0.9926	gi|635078797	phosducin-like protein 3
19.7	19.4	19.0	19.2	19.6	19.4	0.9948	0.9927	gi|635046858	V-type proton ATPase subunit B, brain isoform
19.7	20.1	18.9	19.2	19.8	19.6	0.9938	0.9949	gi|635067027	protein phosphatase 1 regulatory subunit 12A isoform X5
21.1	21.4	20.9	20.9	21.5	21.0	1.0037	0.9952	gi|635018402	hypoxia up-regulated protein 1 isoform X2
18.9	19.4	19.0	18.6	19.7	19.1	1.0047	0.9956	gi|635045445	peptidyl-prolyl cis-trans isomerase D isoform X3
28.0	28.3	28.2	28.0	28.2	28.2	1.0012	0.9964	gi|635113315	peptidyl-prolyl cis-trans isomerase A isoform X1
20.9	19.7	18.7	20.2	20.3	18.8	1.0082	0.9967	gi|635069584	60S ribosomal protein L6
23.8	23.9	23.5	23.6	23.6	23.9	0.9988	0.9973	gi|635077830	ezrin
20.0	20.0	19.7	19.6	20.1	20.0	1.0015	0.9974	gi|635086679	glyoxalase domain-containing protein 4
20.8	20.7	20.5	20.5	20.9	20.6	1.0012	0.9976	gi|635138280	cytosol aminopeptidase
18.8	19.3	18.3	18.3	19.1	19.0	0.9974	0.9978	gi|635059233	integrin alpha-V isoform X3
24.6	25.7	25.1	25.0	25.5	24.9	0.9978	0.9980	gi|635124782	40S ribosomal protein S14 isoform X2
